# Niche space of corals along the Florida reef tract

**DOI:** 10.1371/journal.pone.0231104

**Published:** 2020-04-07

**Authors:** Robert van Woesik, Lynnette M. Roth, Elizabeth J. Brown, Kelly R. McCaffrey, Jacob R. Roth

**Affiliations:** 1 Institute for Global Ecology, Florida Institute of Technology, Melbourne, Florida, United States of America; 2 Independent Researcher, Merritt Island, Florida, United States of America; Department of Agriculture, Water and the Environment, AUSTRALIA

## Abstract

Over the last three decades corals have declined precipitously in the Florida Keys. Their population decline has prompted restoration effort. Yet, little effort has been invested in understanding the contemporary niche spaces of coral species, which could assist in prioritizing conservation habitats. We sought to predict the probability of occurrence of 23 coral species, including the critically endangered *Acropora cervicornis*, using observations at 985 sites from 2011–2015. We ran boosted regression trees to evaluate the relationship between the presence of these corals and eight potential environmental predictors: (i) bathymetry (m), (ii) mean of daily sea surface temperature (SST) (°C), (iii) variance of SST (°C), (iv) range of SST (°C), (v) chlorophyll-*a* concentration (mg m^3^), (vi) turbidity (m^-1^), (vii) wave energy (kJ m^-2^), and (viii) distance from coast (km). The Marquesas and the lower and upper Florida Keys were predicted to support the most suitable habitats for the 23 coral species examined. *A*. *cervicornis* had one of the smallest areas of suitable habitat, which was limited to the lower and upper Florida Keys, the Dry Tortugas, and nearshore Broward-Miami reefs. The best environmental predictors of site occupancy of *A*. *cervicornis* were SST range (4–5°C) and turbidity (*K*_*490*_ between 0.15–0.25 m^-1^). Historically *A*. *cervicornis* was reported in clear oligotrophic waters, although the present results find the coral species surviving in nearshore turbid conditions. Nearshore, turbid reefs may shade corals during high-temperature events, and therefore nearshore reefs in south Florida may become important refuges for corals as the ocean temperatures continue to increase.

## 1. Introduction

Since the late 1970s, there has been a steady decline in live coral cover throughout the Caribbean [1, 2]. This decline has included unprecedented mortality of two of the Caribbean’s most historically important reef-building coral species, *A*. *cervicornis* and *Acropora palmata* [1, 3]. Most of the acroporid mortality in the Caribbean was caused by disease and thermal-stress events [4–6]. In 2006 this decline prompted the listing of both acroporids as ‘threatened’ under the U.S. Endangered Species Act [7], and in 2008 they were listed as ‘critically endangered’ on the International Union for Conservation of Nature Red List. Decades after the initial mortality events, coral populations along the Florida reef tract continue to decline [8–10]. It is therefore critical to quantify the contemporary niche space of coral species as marine diseases become more extensive and destructive [11], and as thermal-stress events become more frequent and intensive [12].

Yet niche space has long been an enigma. Niche theory evolved from Elton’s [13] definition of a species' place in the biological environment and its relationship to food and predators. Later Gause [14] emphasized the role of competitive interactions among species, and Hutchinson [15] delineated a species’ environmentally-driven fundamental niche from its competitive realized niche. MacArthur and May [16] made the implicit assumption that two species could not coexist indefinitely on the same limiting resource, although Roughgarden [17] subsequently advanced niche theory to include low inter-species interactions in high diversity localities. Similarly, Scheffe and van Nes [18] argued that multiple species can coexist in a given niche, which agrees with Hubbell’s [19] assumption of neutrality. However, for coral reefs, such multi-species neutrality may be only relevant in equatorial, benign habitats [20], with higher latitude coral reefs being more susceptible to major physio-chemical drivers such as temperature and light. Superimposed on such niche concepts are background levels of disturbances from hurricanes, thermal-stress events [21], and disease outbreaks [11].

Most contemporary niche models predict the fundamental niche of a species [22, 15], although some niche models include competitive interactions [23] and mechanistic processes [24]. Past niche models along the Florida reef tract suggested that *A*. *cervicornis* colonies were most common on shallow reefs where temperature fluctuations were moderate [25, 26]. Recently, D’Antonio et al. [27] showed that *A*. *cervicornis* colonies were found close to shallow reef ridges, where topography was high. Yet wave energy, irradiance, and water quality also influence the physiology and survival of corals [28–33].

This study predicts the contemporary niche space of 23 coral species, including *A*. *cervicornis*, along the Florida reef tract. Niche space was quantified regionally using niche models [22], which associate the geographic distribution of species with broad-scale environmental conditions. Niche models combine the geographical space of a species with their theoretical-environmental space. The theoretical-environmental space is used to predict the niche of the species, and then that space is projected back onto geographic space, for which environmental conditions are known [34]. The objective of this study was to highlight geographical localities that are most suitable for the survival of 23 coral species along the Florida reef tract. Along with identifying habitats and regions that should be prioritized for conservation purposes, this work could also help guide restoration efforts, highlighting where the outplanting of nursery-reared coral colonies may have the best chance of survival.

## 2. Methods

### Study area and biological data

**Fig 1:**
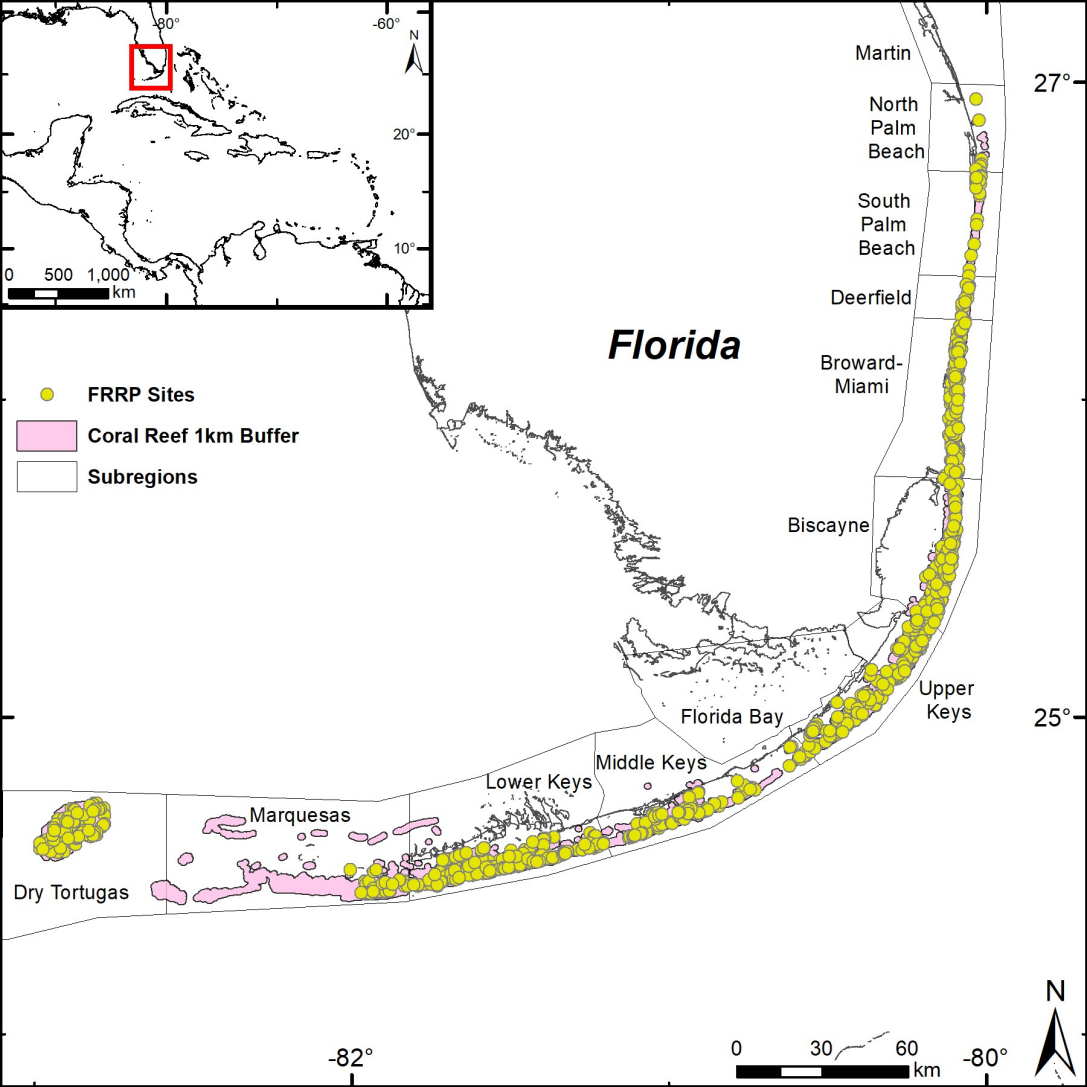
Distribution of 985 study sites used in the niche model along the Florida reef tract from 2011–2015, where FRRP is the Florida Reef Resiliency Project (43 sites were removed prior to use in model). The coral reef layer is a 1km buffer taken from the Florida Fish and Wildlife Conservation Commission-Fish and Wildlife Research Institute’s Unified Florida Reef Tract spatial layer. Basemap from Esri, DigitalGlobe, GeoEye, i-cubed, United States Department of Agriculture Farm Service Agency, United States Geological Survey, Aerials Express (AEX), Getmapping, Aerogrid, French National Mapping Agency (IGN), Instituto Geográfico Português (IGP), swisstopo, and the GIS User Community.

The Florida reef tract runs parallel to the Florida mainland and along the Florida Keys in the southwest (Fig 1). Data on the presence and absence of *A*. *cervicornis* and 31 other species [i.e., *A*. *palmata*, *Agaricia fragilis*, *Agaricia lamarcki*, *Colpophyllia natans*, *Dichocoenia stokesii*, *Diploria labyrinthiformis*, *Eusmilia fastigiata*, *Favia fragum*, *Isophyllia sinuosa*, *Madracis decactis*, *Meandrina meandrites*, *Millepora alcicornis*, *Millepora complanata*, *Montastraea cavernosa*, *Mussa angulosa*, *Mycetophyllia* sp., *Oculina diffusa*, *Orbicella annularis*, *Orbicella faveolata*, *Orbicella franksi*, *Porites astreoides*, *Porites divaricata*, *Porites furcata*, *Porites porites*, *Pseudodiploria clivosa*, *Pseudodiploria strigosa*, *Siderastrea radians*, *Siderastrea siderea*, *Solenastrea bournoni*, *Stephanocoenia intersepta*, *Undaria (Agaricia) agaricites*] were examined along the Florida reef tract using the Florida Reef Resiliency Project (FRRP) monitoring dataset from 2011–2015. Note that the models only predicted niche spaces for 23 of the 32 coral species because nine of the species were reported in less than 10% of the sites, making predictions uncertain. The FRRP data were collected at 1028 sites using 10 m x 1 m (10 m^2^) belt transect surveys (Fig 1). The niche models were trained on a random subset (80%) of the sites and were tested against the remaining 20% of the sites (Table A in S1 File).

#### Environmental data

Eight environmental predictors, which have been previously shown to influence coral physiology and survival [35–37], were initially examined for incorporation in the niche model: (i) bathymetry (m), (ii) mean of daily sea surface temperature (SST) (°C), (iii) variance of SST(°C), (iv) range of SST(°C), (v) chlorophyll-*a* concentration (mg m^3^), (vi) turbidity (m^-1^), (vii) wave energy (kJ m^-2^), and (viii) distance from coast (km) (Table 1). A 1-km resolution depth (m) raster file was developed by Ames [38], which was a combination of satellite data and *in-situ* field measurements. Daily measurements of SST (°C) and chlorophyll-*a* concentration (mg m^-3^), collated as raster data, with a 0.25 km resolution, were obtained from the University of Southern Florida’s Optical Oceanography laboratory from 2011–2015 (https://optics.marine.usf.edu/). The average SST (°C) was calculated as the mean of daily SST from 2011–2015. The variance of SST (°C), a measurement of thermal variability, was calculated as the variance of daily SST from 2011–2015. The range of SST was calculated by subtracting the minimum temperature from the maximum temperature at every pixel. The average daily chlorophyll-*a* concentration was calculated as the mean chlorophyll-*a* concentration from 2011–2015. Turbidity was quantified by the diffuse light attenuation coefficient *K* at 490 nm (m^-1^) from NOAA CoastWatch, averaged daily from 2013–2015. Although pre-2013 *K*_*490*_ imagery exists, it does not include the region of interest and so pre-2013 data were not included in the analysis. Wave energy was calculated using inputs of fetch (i.e., the distance of open ocean over which winds travel unobstructed) calculated using the ‘*fetchR*’ package in R [39], and daily wind speed and wind direction raster data were obtained from Remote Sensing Solutions [40] from 2011–2015. Wave energy was calculated using equations in Chollett and Mumby [41] adapted from [42], where each cell’s fetch was evaluated in the dominant wind direction. If fetch was less than 38 km, then the seas were considered ‘fetch-limited’, whereas if the fetch was greater than 38 km then the seas were considered ‘fully developed.’ A complete explanation of the wave energy calculations is available in the online S1 File. Distance from coast (km) was calculated at the 1-km resolution using the distance function in the ‘*raster*’ package [43] and the coastline polygon from the high-resolution map in the package ‘*RWorldMap*’ [44] in R [45]. For spatial consistency, the final input raster files were resampled to a 1-km resolution and masked to a 1km buffer of the Fish and Wildlife Conservation Unified Florida Reef Tract Map (http://geodata.myfwc.com/datasets/unified-florida-reef-tract-map).

**Table 1:** Environmental data used to produce predictor variables in the niche model. The source resolutions are presented in parentheses. The fifth column indicates whether the variable was used in the niche model (I = included, E = excluded).

Variable	Unit	Source	Period	Niche Model
Bathymetry	m	NOAA (~100 m)	2016	I
Mean daily SST	°C	USF Optical Oceanography Laboratory (250 m)	2011–2015	I
Variance of daily SST	°C	USF Optical Oceanography Laboratory (250 m)	2011–2015	E
Range SST	°C	USF Optical Oceanography Laboratory (250 m)	2011–2015	I
Chlorophyll-*a* concentration	mg m^3^	USF Optical Oceanography Laboratory (250 m)	2011–2015	I
Turbidity (*K*_*490*_)	m^-1^	K_490_ NOAA CoastWatch (250 m)	2013–2015	I
Modeled mean daily wave energy	kJ m^-2^	*fetchR* raster (1 km) Remote Sensing Systems CCMP Wind Vector Analysis Product (0.25°)	2011–2015	I
Distance from Coast	km	*Rworldmap*	2018	I

SST indicates sea surface temperature. Where FWC is the Florida Fish and Wildlife Commission, USF is the University of South Florida, NOAA is the National Oceanic and Atmospheric Administration, and CCMP is Cross-Calibrated Multi-Platform. Note that f*etchR* [39] and *RWorldmap* [44] are packages in R [45].

#### Coral niche model

A niche model was developed initially for 32 coral species along the Florida reef tract at 1028 sites from 2011–2015. However, 9 coral species were found in less than 10% of the sites making those models unstable, therefore the results are not included here in the 23 coral species niche model (Table 2). In addition, because *A*. *cervicornis* is listed as ‘critically endangered’ and is of special interest in this study, an exception to the 10% rule was made for this species. *A*. *palmata* is also of special interest but was only recorded in < 0.5% of the sites (Table 2), which made modeling problematic.

**Table 2:** *In-situ* presence of coral species at sites (%); the modeled area under the receiver operating curve (AUC), which is a diagnostic for model performance; and the percent suitable habitat area (%) predicted by the niche model along the Florida reef tract using data from 985 sites from 2011–2015.

Species Name	Presence at Sites (%)	AUC	Suitable Habitat Area (%)
***Siderastrea radians***	47.67	0.66	80.5
***Siderastrea siderea***	89.4	0.75	75.57
***Stephanocoenia intersepta***	72.28	0.62	65.79
***Solenastrea bournoni***	30.06	0.63	61.54
***Millepora alcicornis***	88.52	0.75	58.34
***Porites astreoides***	80.93	0.68	58.09
***Pseudodiploria strigosa***	30.06	0.64	57.97
***Orbicella faveolata***	30.35	0.62	56.45
***Dichocoenia stokesii***	59.53	0.69	55.48
***Montastraea cavernosa***	64.49	0.69	55.45
***Porites porites***	54.96	0.70	54.86
***Undaria (Agaricia) agaricites***	48.15	0.71	51.36
***Diploria labyrinthiformis***	19.84	0.61	49.88
***Eusmilia fastigiata***	16.44	0.59	48.73
***Pseudodiploria clivosa***	16.05	0.56	46.77
***Colpophyllia natans***	26.65	0.70	46.33
***Porites furcata***	12.55	0.65	46.17
***Porites divaricata***	15.08	0.57	44.7
***Orbicella franksi***	12.26	0.74	36.34
***Mycetophyllia* sp.**	10.31	0.83	32.65
Acropora cervicornis**	7.78	0.69	24.2
***Meandrina meandrites***	31.52	0.57	24.12
***Madracis decactis***	11.28	0.65	22.87
Agaricia fragilis*	1.75	-	-
Agaricia lamarcki*	8.46	-	-
Favia fragum*	4.28	-	-
Isophyllia sinuosa*	0.88	-	-
Millepora complanate*	2.72	-	-
Orbicella annularis*	8.95	-	-
Mussa angulosa*	3.5	-	-
Oculina diffusa*	2.04	-	-
Acropora palmata*	0.39	-	-

Where

* indicates coral species that were not present at >10% of the survey sites and were therefore excluded from the model.

** indicates an exception for the 10% rule, because the critically endangered *A*. *cervicornis* was a species of special interest in this study. The dashes indicate that the coral species were found in less than 10% of the sites, therefore the results are not included.

We used boosted regression trees (BRTs) [46] to fit the presence and absence of the coral species data, at 1028 sites, to seven of the eight potential environmental predictors (Table 1, Table A in S1 File). Variance of SST was excluded as a potential environmental predictor because there was a strong positive correlation between variance of SST and range of SST (0.71, Fig A in S1 File). Data for each environmental factor were then extracted for each site. Any sites that did not have values for all 7 environmental factors were removed. Of the 1028 total sites, 43 were removed: 22 sites had no SST data, 20 sites had no turbidity data, and 7 sites had no wave energy data (6 of which also had no SST data). In total, 985 sites were used in the analysis (Fig 1).

We used *k*-fold partitioning to randomly divide the data into five sections. We used data from four of those sections (i.e., 80% of the data) to train the model and data from one of the sections (i.e., 20%) to test the model. We used a machine-learning algorithm in the form of BRTs to evaluate the relationship between the presence of each species and potential environmental predictors. BRTs fit data by recursively adding ‘trees’ (n-branching nodes) at each iteration—bagged trees take a new bootstrap sample from the training data and choose the next tree that minimizes the ‘loss’ function. We set the bag fraction to 0.8 to introduce some stochasticity into the niche model, which indicates that 80% of the training data were used to fit each individual tree. Additionally, we weighted all sites to generate an equal weight of presences and absences [47]. We built the model using the ‘gbm’ R package [46] and code adapted from [48]. The niche model was set to a tree complexity dependent on number of environmental factors being tested, a learning rate of 0.0015, and an initial condition of 30 trees.

A 1-km buffer of the coral-reef polygons, found within Florida Fish and Wildlife Conservation Commission-Fish and Wildlife Research Institute’s Unified Florida Reef Tract spatial layer, was used to clip the geographical extent of the model results (Fig 1). The amount of suitable habitat was then calculated as the combined area of cells (km^2^), above a 50% threshold value. The stepwise iteration process of the BRTs cross-validates at each iteration using data that were not in the immediate bootstrapped training set. The model was run multiple times using different environmental combinations, based on their performance in previous runs, to optimize the model (i.e., the best area under the receiver operating curve) and remove unpredictive environmental variables. The model was then tested for accuracy against the remaining 20% of the dataset, to produce diagnostics of model performance which included constructing confusion matrices (Tables B and C in S4 File). Confusion matrices were computed using the ‘caret’ package [49] in R [45].

We were also interested in the effects of disturbances on the niche space of *Acropora cervicornis*. Several disturbances occurred either side of our field-sampling window (2011–2015), including a major cold-snap in 2010 [5], Hurricane Irma in 2017, and a disease outbreak (stony-coral-tissue-loss disease) in 2016. Therefore, we took a landscape-metrics approach to simulate the influence of a ‘generic’ disturbance on the predicted patch attributes of *Acropora cervicornis* along the Florida reef tract. Because *Acropora cervicornis* colonies are spread across the Florida reef tract as viable patches of subpopulations, disturbances are likely to remove viable patches from the metapopulation, increasing the average distance among the patches. To test this concept and calculate the relative distances between predicted patches of *Acropora cervicornis* subpopulations on modern reefs along the Florida reef tract, we computed the Euclidean nearest-neighbor distance among the predicted niche patches to examine the average distances between the patches using a series of probability-of-occurrence thresholds. These thresholds simulate different intensities of disturbance, with increasing values simulating increasing intensities of disturbance. All data and R code are available at https://github.com/rvanwoesik/Florida-Niche, and kmz (Google Earth) files of the predicted probability of occurrence of each coral species is available in S4 File.

### Ethics statement

The field data were collected by participants in the Florida Reef Resilience Program Disturbance Response Monitoring (DRM) https://myfwc.com/research/habitat/coral/drm/. Permission to visit the study sites was granted by the Florida Fish and Wildlife Conservation Commission and the National Oceanic and Atmospheric Administration. The field studies did not involve the collection of endangered or protected species.

## 3. Results

### Probability of occurrence

The niche model was run on 23 coral species (Table 2). The coral species *Siderastrea radians* and *S*. *siderea* were predicted to have the largest area of suitable habitat along the Florida reef tract (Table 2, Table 3). *S*. *intersepta*, *S*. *bournoni*, *M*. *alcicornis*, *P*. *astreoides*, and *P*. *strigosa* were also predicted to have large areas of suitable habitat, whereas *A*. *cervicornis* was among the species with the smallest area of suitable habitat along the Florida reef tract (Table 2, Table 3).

**Table 3:** Area of suitable habitat space (km^2^) for the 23 coral species in each of the 10 subregions along the Florida reef tract using data from 985 sites from 2011–2015.

Coral species	North Palm Beach	South Palm Beach	Deerfield	Broward-Miami	Biscayne	Upper Keys	Middle Keys	Lower Keys	Marquesas	Dry Tortugas	Total Area (km^2^)
*Siderastrea radians*	0	0	0	0	328	407	335	404	588	270	2332
*Siderastrea siderea*	13	10	8	97	256	347	286	385	536	233	2171
*Stephanocoenia intersepta*	25	46	17	110	130	181	255	419	494	229	1906
*Solenastrea bournoni*	1	18	11	138	240	361	301	419	277	2	1768
*Porites astreoides*	2	0	0	23	166	163	220	328	520	261	1683
*Pseudodiploria strigosa*	0	0	0	0	146	207	257	382	434	254	1680
*Millepora alcicornis*	17	44	15	67	220	276	222	344	201	270	1676
*Orbicella faveolata*	0	0	0	0	140	258	196	369	466	207	1636
*Dichocoenia stokesii*	0	0	0	1	204	362	283	405	339	0	1594
*Montastraea cavernosa*	31	49	17	96	105	81	178	356	427	253	1593
*Porites porites*	0	0	0	0	116	268	217	284	421	270	1576
*Undaria (Agaricia) agaricites*	0	0	0	0	158	206	213	235	454	222	1488
*Diploria labyrinthiformis*	0	0	0	0	118	220	200	297	369	241	1445
*Eusmilia fastigiata*	0	0	0	0	110	172	225	324	367	202	1400
*Pseudodiploria clivosa*	0	4	4	17	198	264	193	285	246	144	1355
*Porites furcata*	0	0	0	0	210	208	245	160	312	203	1338
*Colpophyllia natans*	0	0	0	0	48	108	187	337	425	226	1331
*Porites divaricata*	0	0	0	0	188	265	306	346	110	80	1295
*Orbicella franksi*	0	0	0	0	105	147	35	54	456	256	1053
*Mycetophyllia* sp.	0	5	5	4	2	6	100	226	367	237	952
*Acropora cervicornis*	0	9	8	91	49	112	22	195	59	156	701
*Meandrina meandrites*	30	52	19	76	27	18	63	123	122	163	693
*Madracis decactis*	29	47	16	44	2	8	2	33	298	188	667
**Model extent (km^2^)**	33	84	43	203	349	433	372	522	588	270	2897
**Average**	7.54	15.33	6.79	40.29	150.63	211.58	204.71	301.33	369.83	201.54	
**Rank**	9	8	10	7	6	3	4	2	1	5	

The coral species are ranked according to their total area of suitable habitat space (km^2^)—depicted in the right-hand column of the table. The subregions are ranked in the last row of the table according to total area (km^2^) of predicted probability of occurrence.

**Fig 2:**
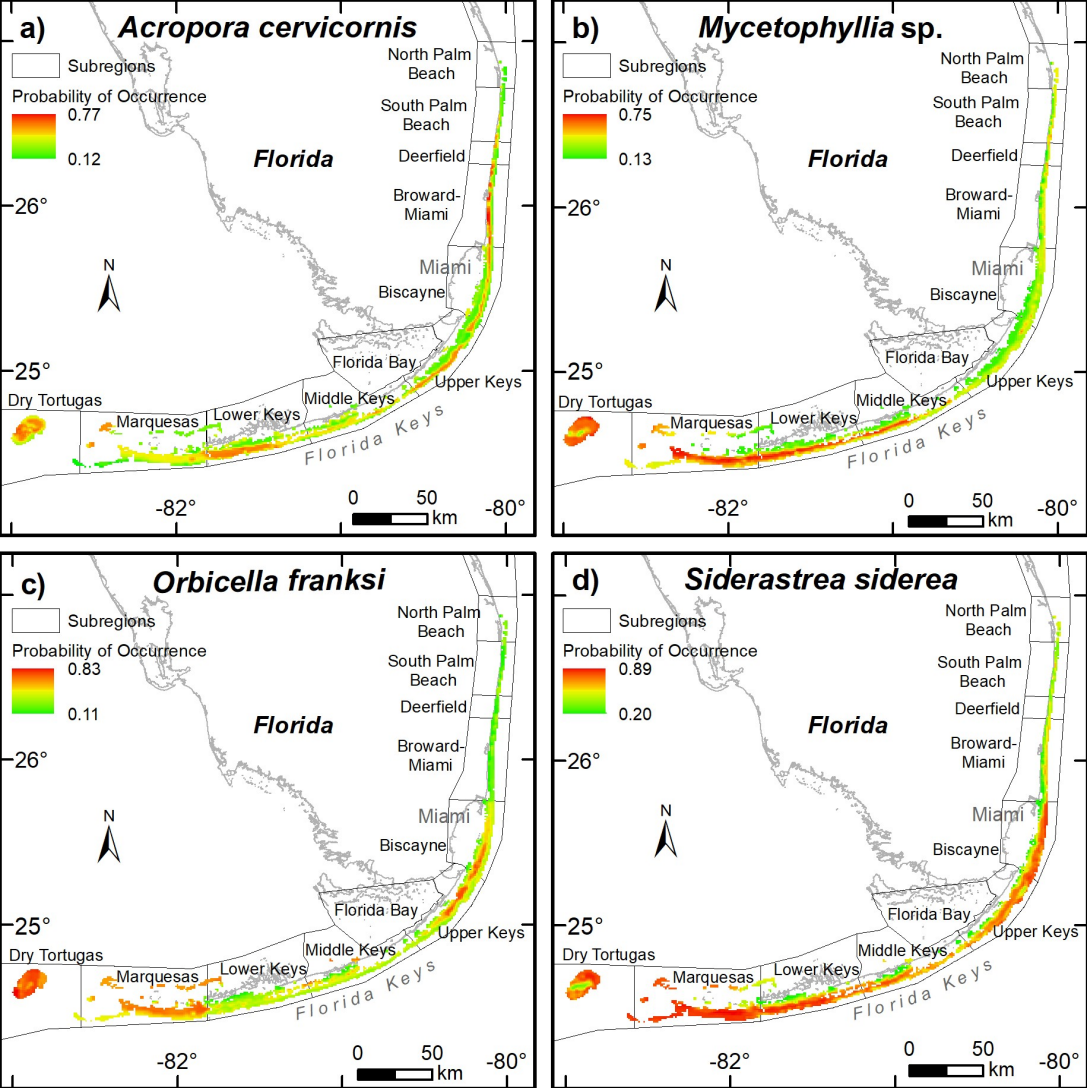
The probability of occurrence of (a) *A*. *cervicornis*, (b) *Mycetophyllia* sp., (c) *O*. *franksi*, and (d) *S*. *siderea* along the Florida reef tract based on the niche model calculated using boosted regression trees using data from 985 sites from 2011–2015.

The Marquesas, the lower Florida Keys, and the upper Florida Keys were predicted to support the most suitable habitats for the coral species examined (Table 3, Table D in S4 File). The subregions north of Deerfield were predicted to support the lowest suitable habitats for most of the coral species examined, although the more northern habitats had a high probability of occurrence for *M*. *cavernosa*, *M*. *decactis*, *M*. *meandrites*, *M*. *alcicornis*, and *S*. *intersepta* (Table 3, Table D in S4 File). For illustrative purposes (Fig 2) we focus on 4 contrasting coral species: *A*. *cervicornis*, *Mycetophyllia* sp., *O*. *franksi*, and *S*. *siderea*, although maps showing the predicted probability of occurrences of all 23 coral species are presented in the online supporting document (Figs B–X in S1–S4 Files). The geographical subregions with the highest probability of occurrence of the critically endangered species *Acropora cervicornis* included the lower Florida Keys (195 km^2^), the Dry Tortugas (156 km^2^), the upper Florida Keys (112 km^2^), and nearshore Broward-Miami reefs (Table 3, Fig 2). The probability of occurrence was lower at Deerfield to South Palm Beach, and the reefs north of South Palm Beach were predicted as unsuitable for *Acropora cervicornis* at the time of surveys (Table 3, Fig 2). *Mycetophyllia* sp. was predicted to occur from the Dry Tortugas through to the middle Florida Keys, whereas the probability of occurrence of *O*. *franksi* was more restricted, and only included the upper Florida Keys and Biscayne. *S*. *siderea* had a wide geographic extent and had a particularly high probability of occurrence from the Dry Tortugas through to Miami (Fig 2, Table 3).

### Environmental predictors

**Fig 3:**
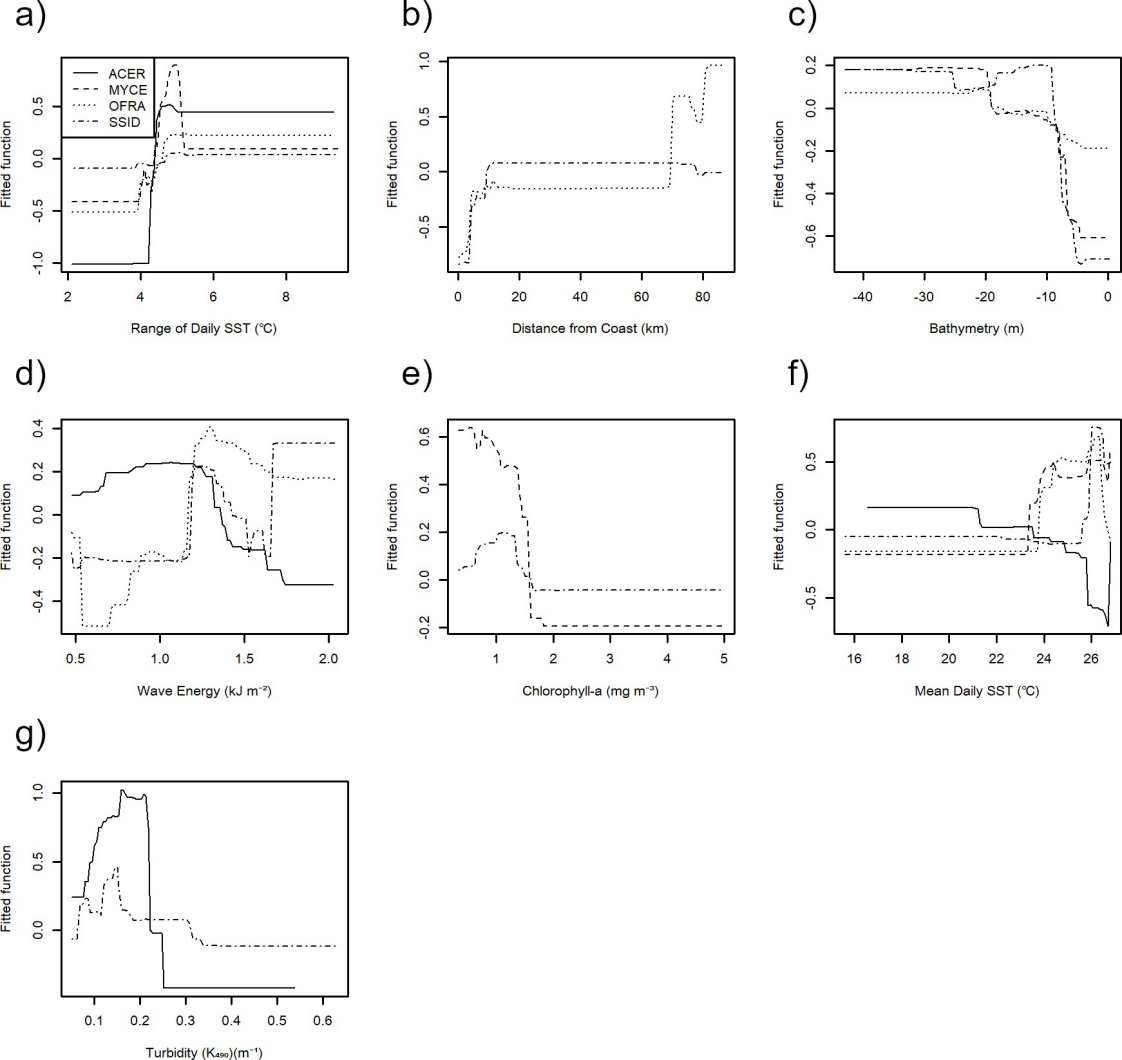
a-g) Partial dependency plots for the 7 predictor variables for the 4 coral species *A*. *cervicornis*, *Mycetophyllia* sp., *O*. *franksi*, and *S*. *siderea* along the Florida reef tract from 985 sites from 2011–2015 as calculated using boosted-regression tree models.

Distance from the coast, range of SST, bathymetry, and wave energy were the 4 most consistent predictors of the 23 coral species examined (Table 4). Again, for illustrative purposes, we focus on four contrasting coral species (*A*. *cervicornis*, *Mycetophyllia* sp., *O*. *franksi*, and *S*. *siderea*) (Fig 3), although the partial dependency plots that outline the best environmental predictors of the 23 coral species are presented in the online supporting document (Figs B–X in S1–S4 Files).

**Table 4:** Summary of relative influence of the 7 influential predictor variables (listed from greatest to least influence, left to right) that created the most accurate niche model for 23 coral species the values are shown as percentages (rows sum to 100%) with the primary variable highlighted in blue for each species using Florida reef tract data from 985 sites from 2011–2015.

Species Name	Dist. from Coast	Range SST	Bathymetry	Wave Energy	Chl-a conc	Mean SST	Turbidity
*Undaria agaricites*	55.84	10.26	5.73	14.64	-	-	13.54
*Porites porites*	41.30	-	17.61	17.21	13.77	10.11	-
*Porites astreoides*	36.47	-	26.62	10.16	-	6.57	20.18
*Eusmilia fastigiata*	35.91	8.94	16.17	24.69	14.28	-	-
*Millepora alcicornis*	33.59	5.65	5.04	17.50	8.72	24.81	4.69
*Orbicella franksi*	31.54	14.35	7.45	21.73	-	24.93	-
*Diploria labyrinthiformis*	26.73	24.18	15.13	22.35	-	-	11.60
*Siderastrea siderea*	19.34	3.51	17.14	18.48	5.23	17.26	19.04
*Mycetophyllia* sp.	-	43.94	20.59	-	17.74	17.74	
*Acropora cervicornis*	-	35.19	-	11.93	-	21.35	31.54
*Colpophyllia natans*	17.95	31.04	17.23	4.62	5.29	12.15	11.71
*Pseudodiploria clivosa*	-	28.62	22.13	9.01	-	19.43	20.81
*Orbicella faveolata*	19.41	25.13	17.06	20.66	-	17.73	-
*Pseudodiploria strigosa*	20.06	30.54	32.26	17.13	-	-	-
*Stephanocoenia intersepta*	15.82	18.89	30.09	14.38	-	16.70	4.13
*Meandrina meandrites*	9.85	13.05	30.01	10.58	27.42	-	9.10
*Montastraea cavernosa*	11.00	19.40	26.14	19.91	9.81	13.74	-
*Siderastrea radians*	-	4.65	5.86	46.76	-	32.37	10.36
*Porites furcata*	-	12.36	21.66	45.50	-	20.48	-
*Porites divaricata*	7.90	21.57	-	35.92	-	11.68	22.93
*Madracis decactis*	-	8.86	17.25	-	54.47	-	19.42
*Solenastrea bournoni*	18.29	21.73	8.40	18.26	33.32	-	-
*Dichocoenia stokesii*	-	-	26.21	8.46	18.81	46.52	-

The 7 predictor variables include: distance from coast (km), range of SST (°C), bathymetry (m), wave energy (kJ m^-2^), chlorophyll-*a* concentration (mg m^3^), mean of daily sea surface temperature (SST) (°C), and turbidity (m^-1^). Variance of SST (°C) was excluded as a potential environmental predictor because there was a strong positive correlation between variance of SST and range of SST. Dashes indicate minimal significance of predictive variable.

The best environmental predictors of site occupancy of *A*. *cervicornis* were SST, moderate turbidity (*K*_*490*_ 0.15–0.25 m^-1^), and moderate wave energy (>0.5–1.5 kJ m^-2^) (Table 4, Fig 3). The probability of occurrence of *A*. *cervicornis* was lower where wave energy was > 1.5 kJ m^-2^ (Table 4, Fig 3). *Mycetophyllia* sp., had the highest probability of occurrence when the temperature range was between 4–6°C, the bathymetry was deeper than 5 m, the chlorophyll-a concentrations were below 1.5 mg m^3^, and the mean SST was 24 ^o^C (Table 4, Fig 3). The highest probability of occurrence for *O*. *franksi* occurred in the Dry Tortugas, > 60 km from shore, where wave energy was relatively high (1.2–1.5 kJ m^-2^), and where mean SST was around 25 ^o^C (Table 4, Fig 3). Note the flat line in Fig 3B represents a lack of sampling sites between 10 km and 70 km from shore. *S*. *siderea* had the highest probability of occurrence in habitats that had low chlorophyll-a concentrations (< 0.3 mg m^3^), mean SST around 26 ^o^C, and wave energy between 1.2–1.5 kJ m^-2^ (Table 4, Fig 3).

## 4. Discussion

In the last four decades the Florida reef tract has lost a significant proportion of coral populations, particularly the reef-building corals *Orbicella* and *Acropora* species [1, 2, 50]. The niche models predicted that 13 species of coral were likely to have favorable habitats from the Dry Tortugas to Miami. These species included: *D*. *stokesii*, *D*. *labyrinthiformis*, *E*. *fastigiata*, *M*. *alcicornis*, *O*. *faveolata*, *P*. *astreoides*, *P*. *divaricata*, *P*. *furcata*, *P*. *clivosa*, *S*. *radians*, *S*. *siderea*, *S*. *bournoni*, *S*. *intersepta* (S3 File for Google Earth kmz files). Seven coral species appeared more sensitive to the environmental variables examined, and their probability of occurrence was patchy (Table 3). These species included *A*. *cervicornis*, *C*. *natans*, *M*. *decactis*, *M*. *meandrites*, *M*. *cavernosa*, *Mycetophyllia* sp., and *O*. *franksi*. For example, the niche model showed that the lower and upper Florida Keys, the Dry Tortugas, and nearshore Broward-Miami County reefs were likely to support *A*. *cervicornis*, but the middle Florida Keys was less likely to support this species. Similarly, the reef-building coral *O*. *faveolata* was predicted to favor the habitats in the upper and lower Florida Keys rather than the middle Florida Keys (Table 3). Ginsburg and Shinn [51] first reported on the negative influence of Florida Bay on the middle Florida Keys, and recently Toth et al. [52] showed that reef accretion terminated significantly earlier in the middle Florida Keys than elsewhere, which they suggested was most likely because of negative influences from Florida Bay. It is likely that Florida Bay will continue to influence reefs in the middle Florida Keys, which may prove a disadvantage for many coral species along those reefs.

The best environmental predictors of site occupancy of the ‘critically endangered’ *A*. *cervicornis* were moderate turbidity, SST, and wave energy. Although historically *Acropora* corals are known to survive best in oligotrophic waters [53–55], the present results suggest that on modern reefs, near zero turbidity was not optimal for *A*. *cervicornis* [56]. Rather, a moderate turbidity value *K*_*490*_ of between 0.15–0.25 m^-1^ showed the highest probability of occurrence. These results agree with physiological studies, which show that reducing light by shading can effectively reduce the influence of temperature anomalies [57–59]. Indeed, thermal-stress events may be shifting the optimal niche space of corals toward more turbid habitats, for example to nearshore reefs of Broward-Miami subregion, as ocean temperatures increase [56]. The present study also suggests that moderate wave energy (>0.5–1.5 kJ m^-2^) is favorable for *A*. *cervicornis*. In support, D’Antonio et al. [27] showed that colonies of *A*. *cervicornis* were most common close to reef edges, where water-flow rates were high. Physiological experiments also show *Acropora* colonies are particularly intolerant to stagnant waters, with low rates of mass transfer [60].

Although the niche models were 87% accurate at predicting localities for restoration for *A*. *cervicornis*, and 88% accurate for *O*. *franksi* and *Mycetophyllia* sp. (Table C in S4 File), there are some caveats that need consideration. Firstly, these types of models suffer from incomplete geographic sampling and mismatches of scale between the organism and environmental covariates. For example, an observed absence of a coral in a 10 m^2^ belt transect in the field does not necessarily imply a complete absence throughout a 1-km^2^ grid cell, at which the environmental variables are considered. Therefore, an absence might not be considered a ‘true absence’ [61] and would reduce the model’s predictive capacity. Secondly, while a dominant species might occupy most of its fundamental niche space, rare species might occupy only a small proportion of their fundamental niche [62]. Dispersion limitation may further prevent the rare species from occupying all the potential niche space, and therefore predicting the probability of occupancy may be over-estimated (i.e., with high false positives).

Since niche models are known to be prevalence-dependent [63], low *in-situ* occurrences will translate to low accuracies. Indeed, the niche models had high specificity and low sensitivity (see S4 File for full specificity and sensitivity results). Specificity is an indicator of how good the model is at detecting true negatives, whereas sensitivity is an indicator of how good the model is at detecting true positives. In other words, the niche models were good at predicting habitats in which a particular species was unlikely to be present, but less accurate at predicting habitats in which a species could occur. This strong specificity and low sensitivity are expected in localities such as the Florida reef tract, where the organisms do not occupy the entire niche space because the system has undergone recent disturbances. Such disturbed environments reflect data that are unbalanced toward absences, although we did compensate for this issue by generating an equal weight of presence and absence sites [47]. The simulations that examined disturbances to the predicted patches of *Acropora cervicornis*, using a series of probability-of-occurrence thresholds, found that patch distances were on average 2–3 km (Fig Y in S4 File) on modern reefs along the Florida reef tract, and that distances between the patches are likely to increase with an increase in disturbances. These results are troublesome considering the fragile nature of the modern metapopulation of *Acropora cervicornis*. The niche models can be improved in the future by hierarchically adding spatial and temporal complexity, although we expect that the general geographic patterns will hold up. The models can also be improved by adding more local information, such as macroalgal cover, since low macroalgae coverage has been shown to increase the survival of *A*. *cervicornis* [64].

## 5. Summary and conclusions

Recent changes in environmental conditions along the Florida reef tract may have shifted, and even narrowed, the niche space of some sensitive coral species [8], and consequently information on the distribution of coral species from decades past may no longer provide information for present niche space. Previously, the most optimal purported niche space for *A*. *cervicornis* was in clear oligotrophic waters, although the present results suggest that turbid conditions are more optimal on modern reefs that frequently experience high heat stress. Therefore, the nearshore reefs along the Florida reef tract may become important refuges for corals as the ocean temperatures continue to increase.

## Supporting information

S1 File (DOCX)

S2 File (DOCX)

S3 File (DOCX)

S4 File (DOCX)

S5 File (ZIP)
