# Supplementary material for: Niche space of corals along the Florida reef tract
Source: PLoS One. 2020 Apr 7;15(4):e0231104. doi: 10.1371/journal.pone.0231104 (PMC7138326; doi:10.1371/journal.pone.0231104)
Supplement: S1 File — (DOCX) [file pone.0231104.s001.docx]

**Supplementary document for:** **Coral niche space in Florida**

Robert van Woesik^1,*^, Lynnette Roth^2^, Elizabeth J Brown^1^, Kelly R McCaffrey^1^, Jacob Roth^2^

**Equations and data to produce wave energy raster files**

Wave energy spatial distribution models were created following the methods developed by Ekebom et al. (2003) and revised by Chollett and Mumby (2012). The non-dimensional fetch (ξ) for each pixel was calculated as,

$\xi=\frac{gF}{U_{10}^{2}}$ (1),

where *F* is the previously calculated average fetch in meters, *U_10_* is the wind speed at an elevation of 10 m in ms^-1^, and *g* is the acceleration due to gravity (9.81 ms^-2^). For fetch-limited pixels, wave height (*H_mo_*) and wave period (*T_m_*) can be calculated with:

$H_{mo}=0.00082\times U_{10}^{1.1}\times F^{0.45}$ (2),

$T_{m}=0.087\times U_{10}^{1.1}\times F^{0.27}$ (3).

A pixel was classified as fetch-limited when the non-dimensional fetch (*ξ*) was equal to 38,590 m. If pixels were not fetch-limited, wave height and exposure were calculated by:

$H_{mo}=0.034\times U_{10}^{2}$ (4),

$T_{m}=0.081\times U_{10}$ (5).

The total wave energy of the system, *WE* (Joules), was calculated using:

$WE=\frac{1}{16}\rho gH_{mo}^{2}$ (6),

where *ρ* is the density of sea water (1,030 kg m^-3^). These values were calculated daily from 1987 to 2015, for all pixels over the study area at a 1 km resolution. The average daily wave energy, *E*, was found per pixel by taking the geometric mean of the daily energy estimates. Daily data files of all variables were imported into R, where all values greater than zero were used to determine the range and mean of the variables for the periods of interest.**
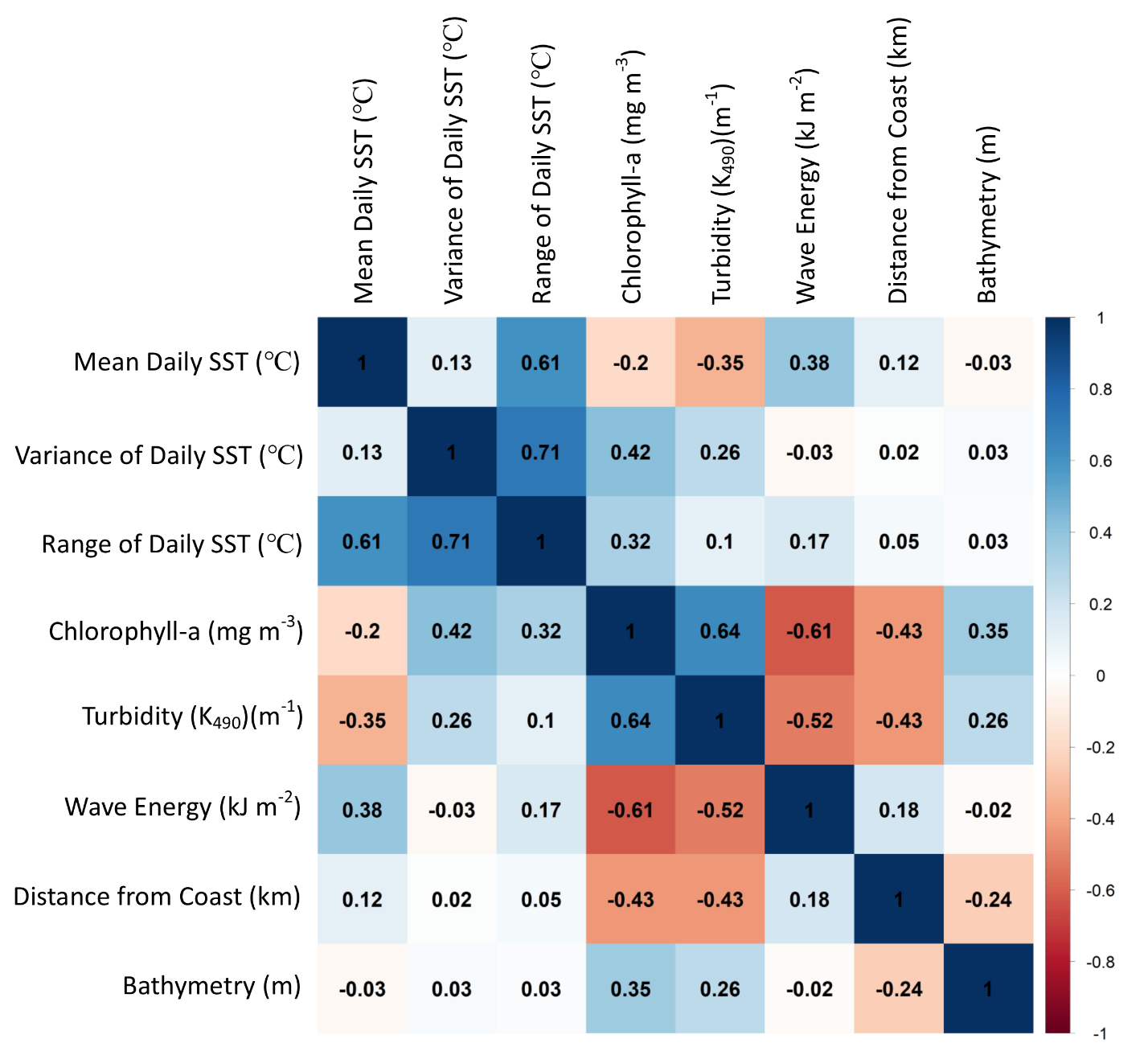
**

**Figure A***.* Correlation matrix of 8 potential environmental predictors at 985 sites along the Florida reef tract from 2011–2015, except for the turbidity data which was from 2013–2015.

**Table A.** Presence and absence sites of 23 coral species that were modeled along the Florida reef tract from 2011–2015, and the training and testing presence and absence after *k*-fold partitioning. There were a total of 1028 sites surveyed, although 43 sites were removed for the models because they missed one or more environmental variable, leaving 985 sites for the training and test data.

| Species | Total Presence | Total Absence | Training Presence | Training Absence | Test Presence | Test Absence |
| --- | --- | --- | --- | --- | --- | --- |
| *Acropora cervicornis* | 76 | 909 | 57 | 731 | 19 | 178 |
| *Undaria (Agaricia) agaricites* | 478 | 507 | 385 | 403 | 93 | 104 |
| *Colpophyllia natans* | 270 | 715 | 214 | 574 | 56 | 141 |
| *Dichocoenia stokesi* | 605 | 380 | 471 | 317 | 134 | 63 |
| *Pseudodiploria clivosa* | 155 | 830 | 128 | 660 | 27 | 170 |
| *Diploria labyrinthiformis* | 199 | 786 | 156 | 632 | 43 | 154 |
| *Pseudodiploria strigosa* | 288 | 697 | 231 | 557 | 57 | 140 |
| *Eusmilia fastigiata* | 168 | 817 | 132 | 656 | 36 | 161 |
| *Madracis decactis* | 111 | 874 | 91 | 697 | 20 | 177 |
| *Meandrina meandrites* | 317 | 668 | 253 | 535 | 64 | 133 |
| *Millepora alcicornis* | 885 | 100 | 711 | 77 | 174 | 23 |
| *Montastraea cavernosa* | 645 | 340 | 528 | 260 | 117 | 80 |
| *Orbicella faveolata* | 302 | 683 | 242 | 546 | 60 | 137 |
| *Orbicella franksi* | 124 | 861 | 96 | 692 | 28 | 169 |
| *Mycetophyllia sp.* | 106 | 879 | 81 | 707 | 25 | 172 |
| *Porites astreoides* | 806 | 179 | 638 | 150 | 168 | 29 |
| *Porites divaricata* | 152 | 833 | 117 | 671 | 35 | 162 |
| *Porites furcata* | 128 | 857 | 103 | 685 | 25 | 172 |
| *Porites porites* | 549 | 436 | 433 | 355 | 116 | 81 |
| *Siderastrea radians* | 465 | 520 | 365 | 423 | 100 | 97 |
| *Siderastrea siderea* | 888 | 97 | 716 | 72 | 172 | 25 |
| *Solenastrea bournoni* | 304 | 681 | 245 | 543 | 59 | 138 |
| *Stephanocoenia intersepta* | 728 | 257 | 579 | 209 | 149 | 48 |

**
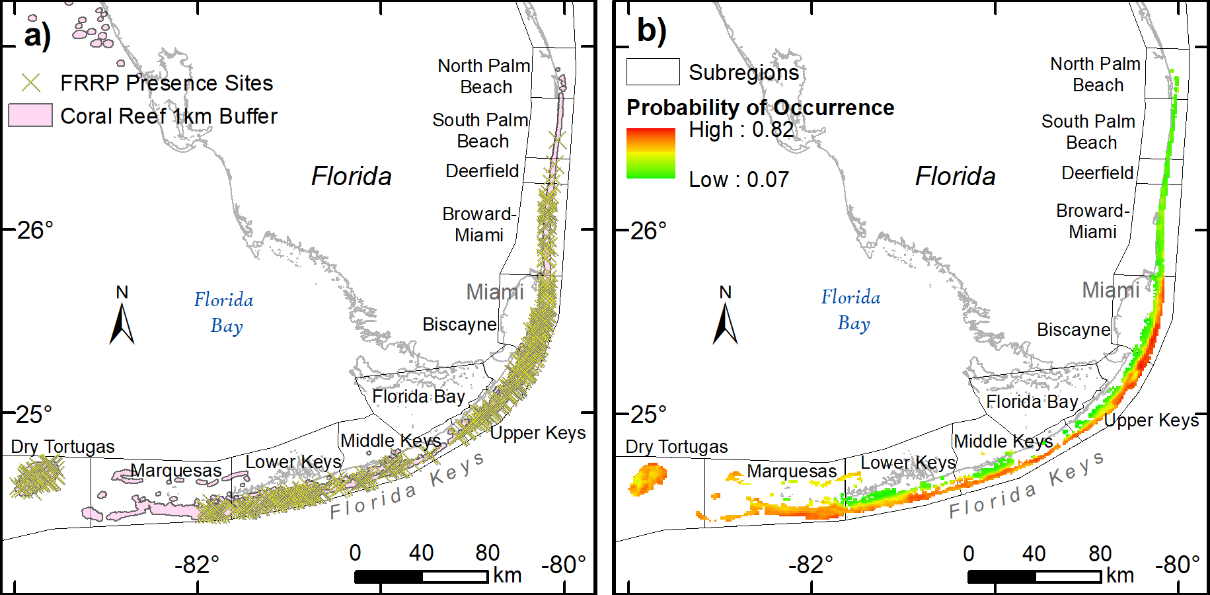
**

**
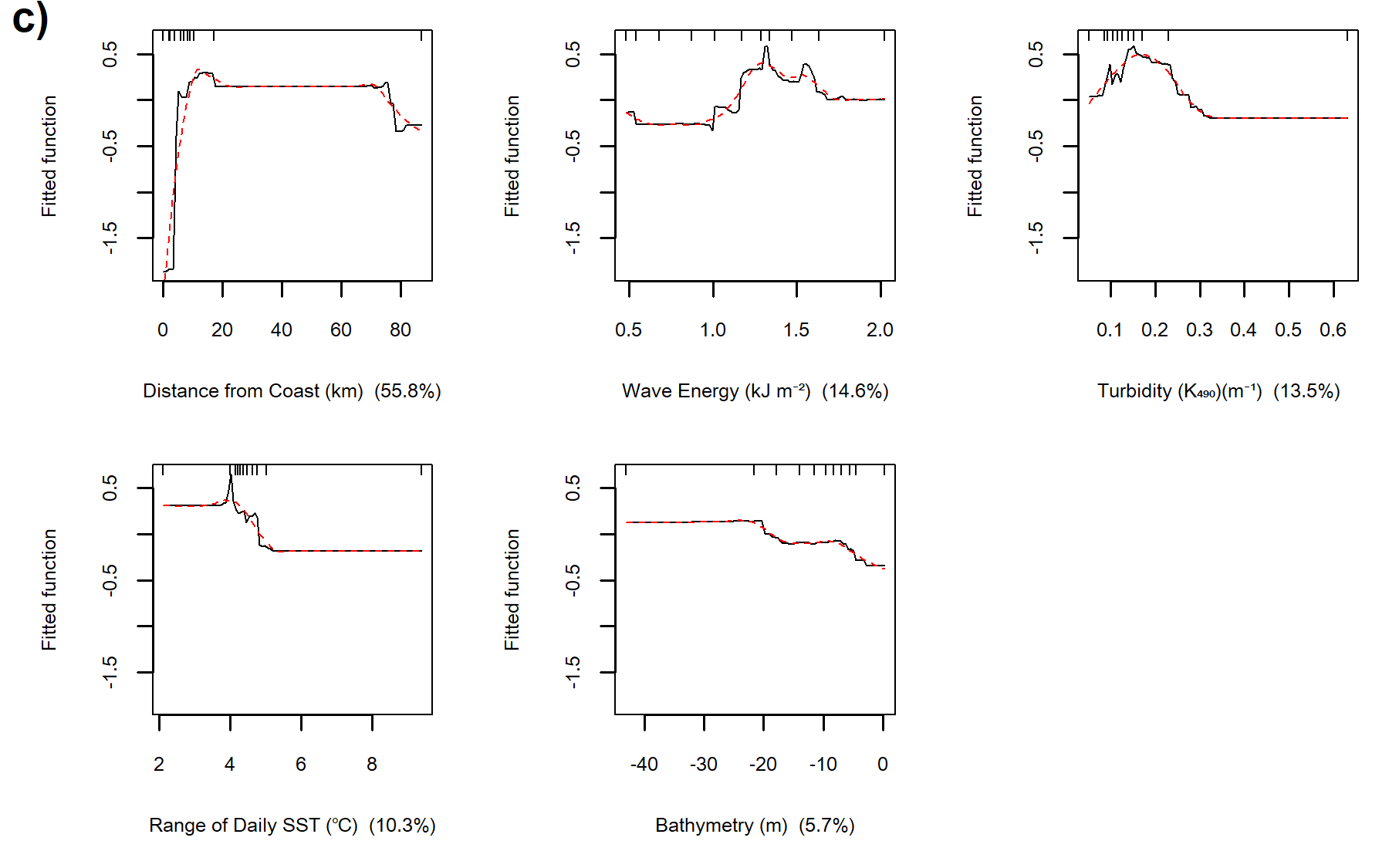
**

**Figure B.** a) Presence locations of *Undaria (Agaricia) agaricites* used to train and test the niche model along the Florida reef tract from 2011–2015. FRRP is the Florida Reef Resiliency Project (FRRP) (yellow crosses, n = 478). Absence locations are not shown. The coral reef layer is a 1 km buffer taken from the Florida Fish and Wildlife Conservation Commission Fish and Wildlife Research Institute’s Unified Florida Reef Tract spatial layer. Basemap: Esri, DigitalGlobe, GeoEye, i-cubed, USDA FSA, USGS, AEX, Getmapping, Aerogrid, IGN, IGP, swisstopo, and the GIS User Community. b) Probability of occurrence of *Undaria (Agaricia) agaricites*. Our niche model provides a probability map highlighting where these corals will experience ‘suitable’ environmental conditions for restoration. c) Fitted function plots of the suite of 5 predictor variables that created the most accurate model output for *Undaria (Agaricia) agaricites.* The height of the function above or below the “0” mark shows to what degree the suitable habitat is affected, within the range of each variable. The percentage within the parentheses shows the influence of each variable on the model.


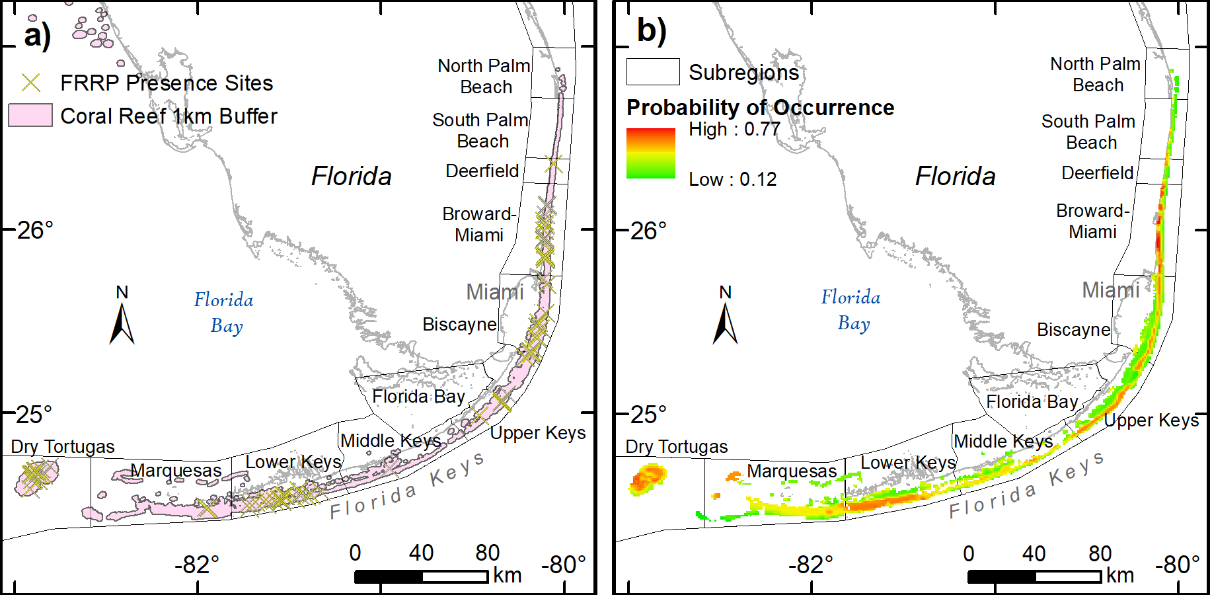


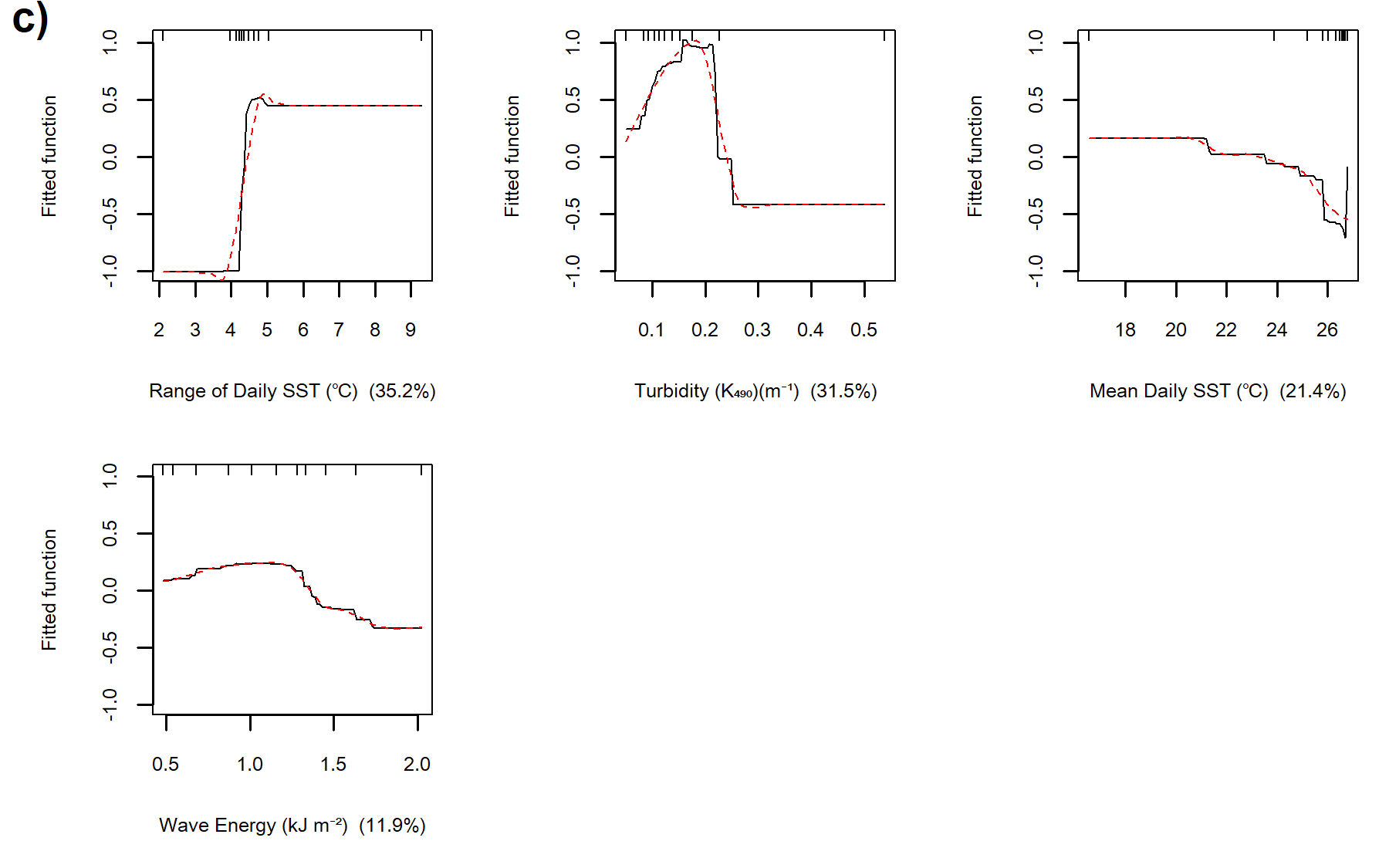


**Figure C.** a) Presence locations of *Acropora cervicornis* used to train and test the niche model along the Florida reef tract from 2011–2015. FRRP is the Florida Reef Resiliency Project (FRRP) (yellow crosses, n = 76). Absence locations are not shown. The coral reef layer is a 1 km buffer taken from the Florida Fish and Wildlife Conservation Commission Fish and Wildlife Research Institute’s Unified Florida Reef Tract spatial layer. Basemap: Esri, DigitalGlobe, GeoEye, i-cubed, USDA FSA, USGS, AEX, Getmapping, Aerogrid, IGN, IGP, swisstopo, and the GIS User Community. b) Probability of occurrence of *Acropora cervicornis*. Our niche model provides a probability map highlighting where these corals will experience ‘suitable’ environmental conditions for restoration. c) Fitted function plots of the suite of 4 predictor variables that created the most accurate model output for *Acropora cervicornis.* The height of the function above or below the “0” mark shows to what degree the suitable habitat is affected, within the range of each variable. The percentage within the parentheses shows the influence of each variable on the model.


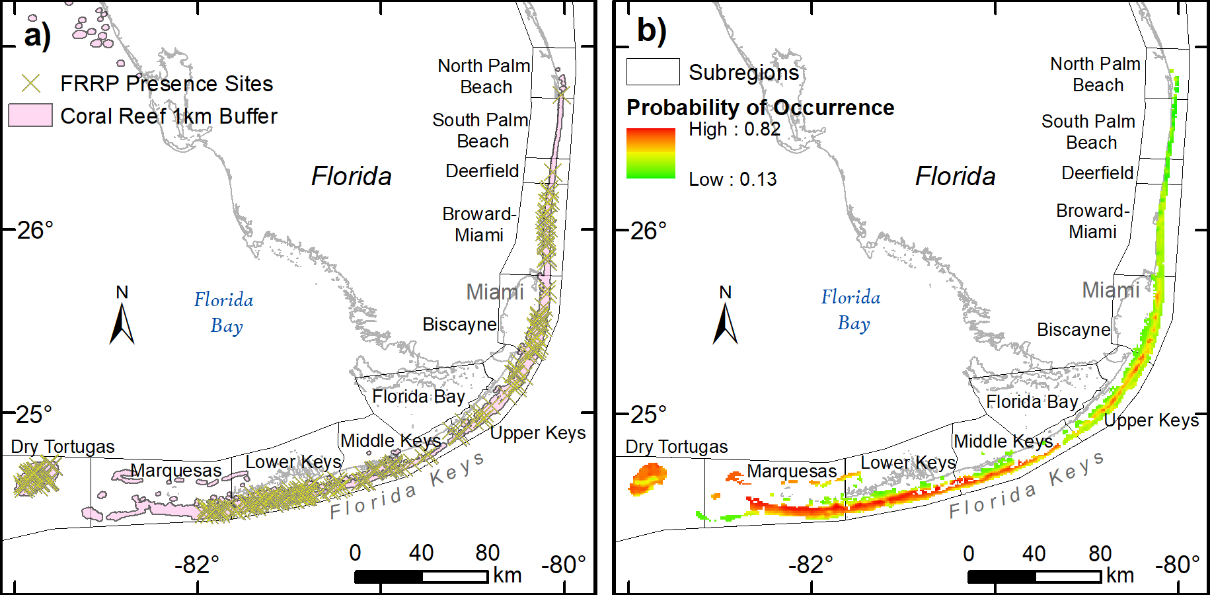

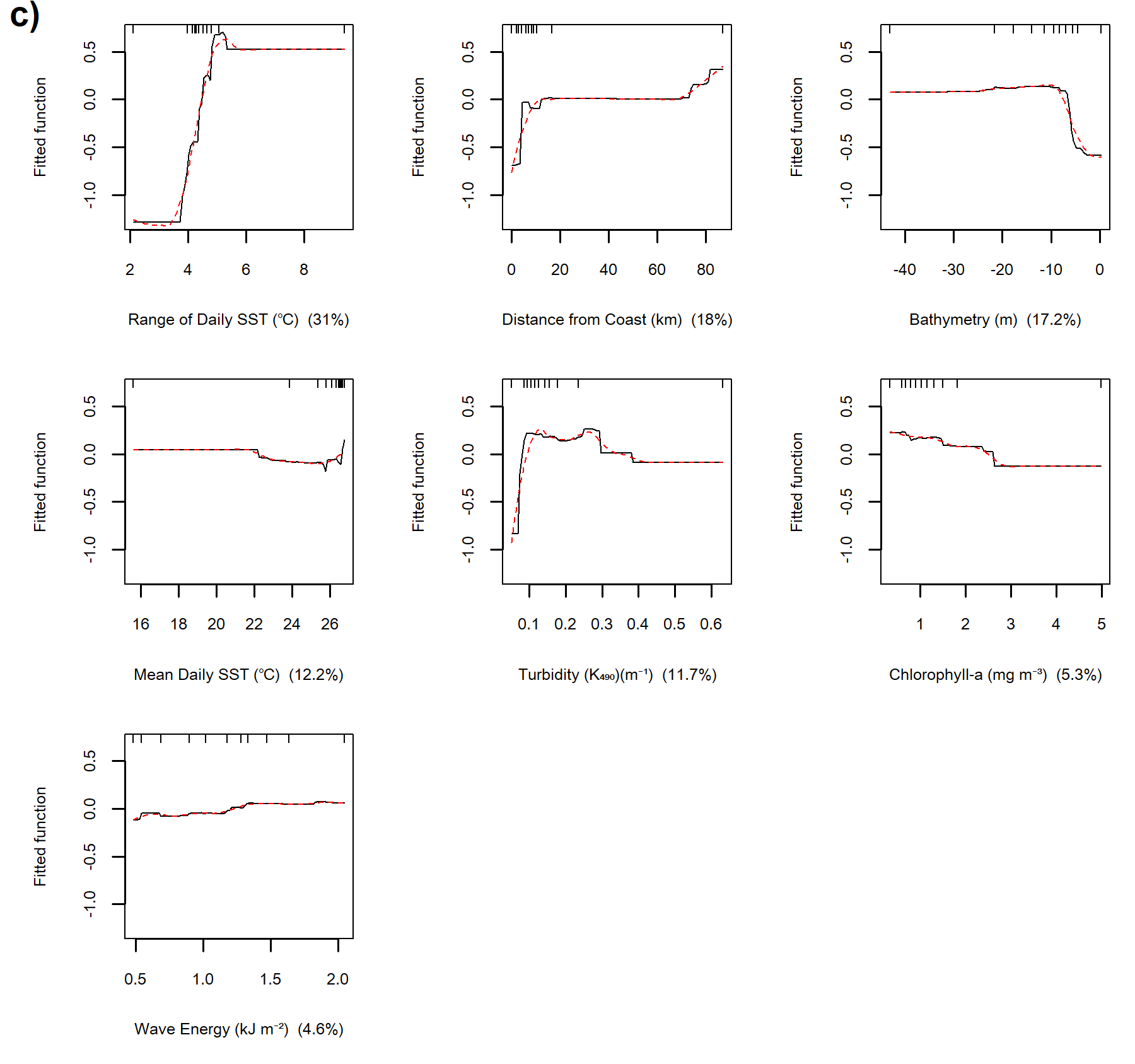


**Figure D.** a) Presence locations of *Colpophyllia natans* used to train and test the niche model along the Florida reef tract from 2011–2015. FRRP is the Florida Reef Resiliency Project (FRRP) (yellow crosses, n = 270). Absence locations are not shown. The coral reef layer is a 1 km buffer taken from the Florida Fish and Wildlife Conservation Commission Fish and Wildlife Research Institute’s Unified Florida Reef Tract spatial layer. Basemap: Esri, DigitalGlobe, GeoEye, i-cubed, USDA FSA, USGS, AEX, Getmapping, Aerogrid, IGN, IGP, swisstopo, and the GIS User Community. b) Probability of occurrence of *Colpophyllia natans*. Our niche model provides a probability map highlighting where these corals will experience ‘suitable’ environmental conditions for restoration. c) Fitted function plots of the suite of 7 predictor variables that created the most accurate model output for *Colpophyllia natans.* The height of the function above or below the “0” mark shows to what degree the suitable habitat is affected, within the range of each variable. The percentage within the parentheses shows the influence of each variable on the model.


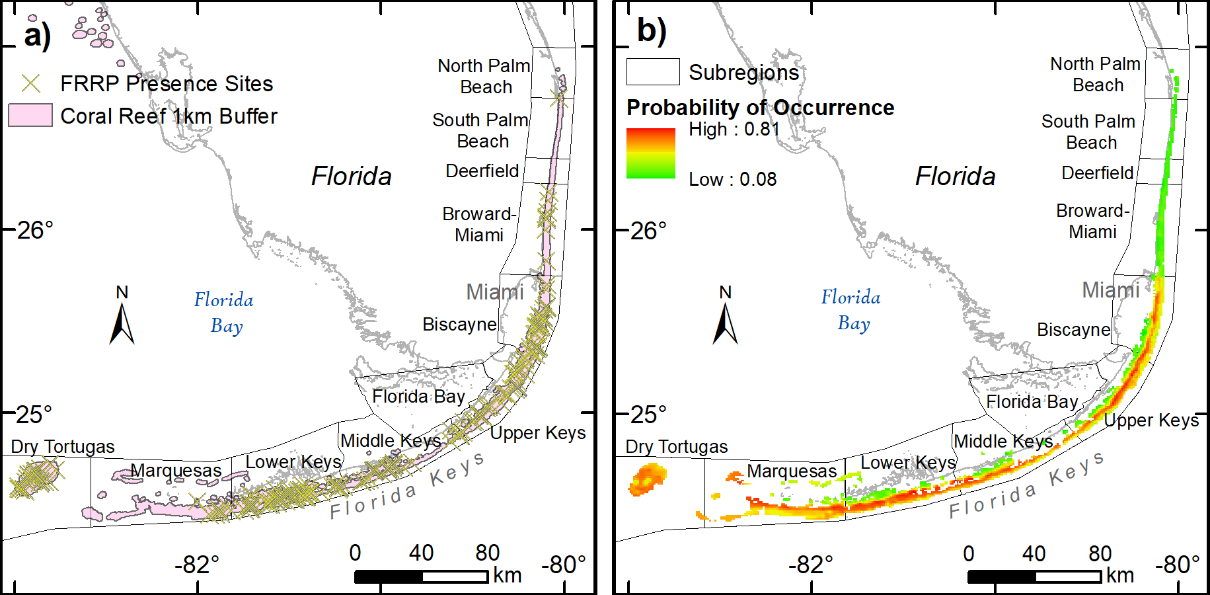


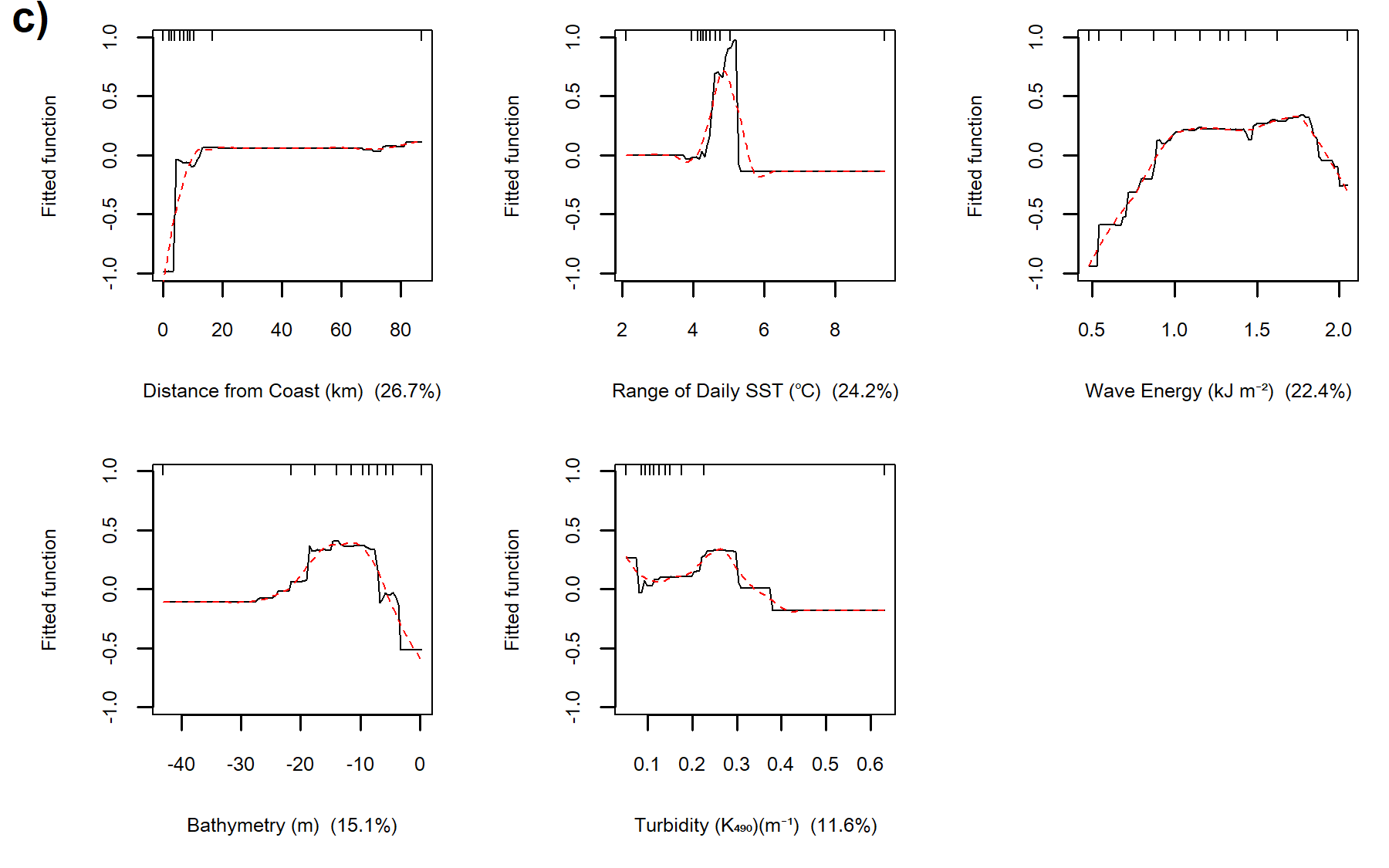


**Figure E.** a) Presence locations of *Diploria labyrinthiformis* used to train and test the niche model along the Florida reef tract from 2011–2015. FRRP is the Florida Reef Resiliency Project (FRRP) (yellow crosses, n = 199). Absence locations are not shown. The coral reef layer is a 1 km buffer taken from the Florida Fish and Wildlife Conservation Commission Fish and Wildlife Research Institute’s Unified Florida Reef Tract spatial layer. Basemap: Esri, DigitalGlobe, GeoEye, i-cubed, USDA FSA, USGS, AEX, Getmapping, Aerogrid, IGN, IGP, swisstopo, and the GIS User Community. b) Probability of occurrence of *Diploria labyrinthiformis*. Our niche model provides a probability map highlighting where these corals will experience ‘suitable’ environmental conditions for restoration. c) Fitted function plots of the suite of 5 predictor variables that created the most accurate model output for *Diploria labyrinthiformis.* The height of the function above or below the “0” mark shows to what degree the suitable habitat is affected, within the range of each variable. The percentage within the parentheses shows the influence of each variable on the model.


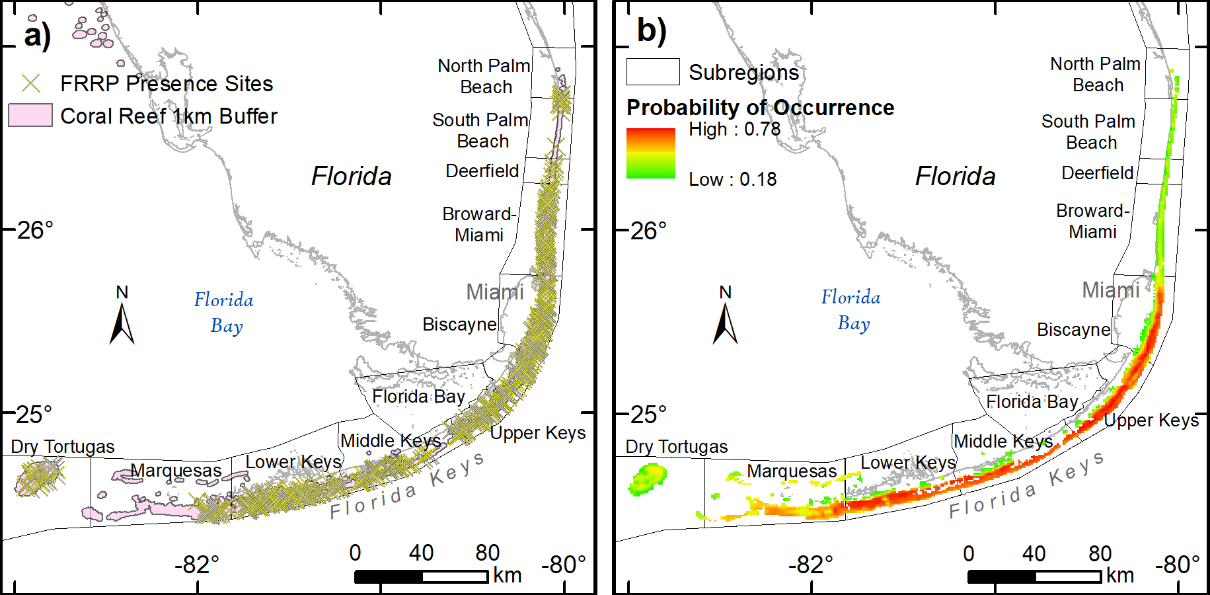


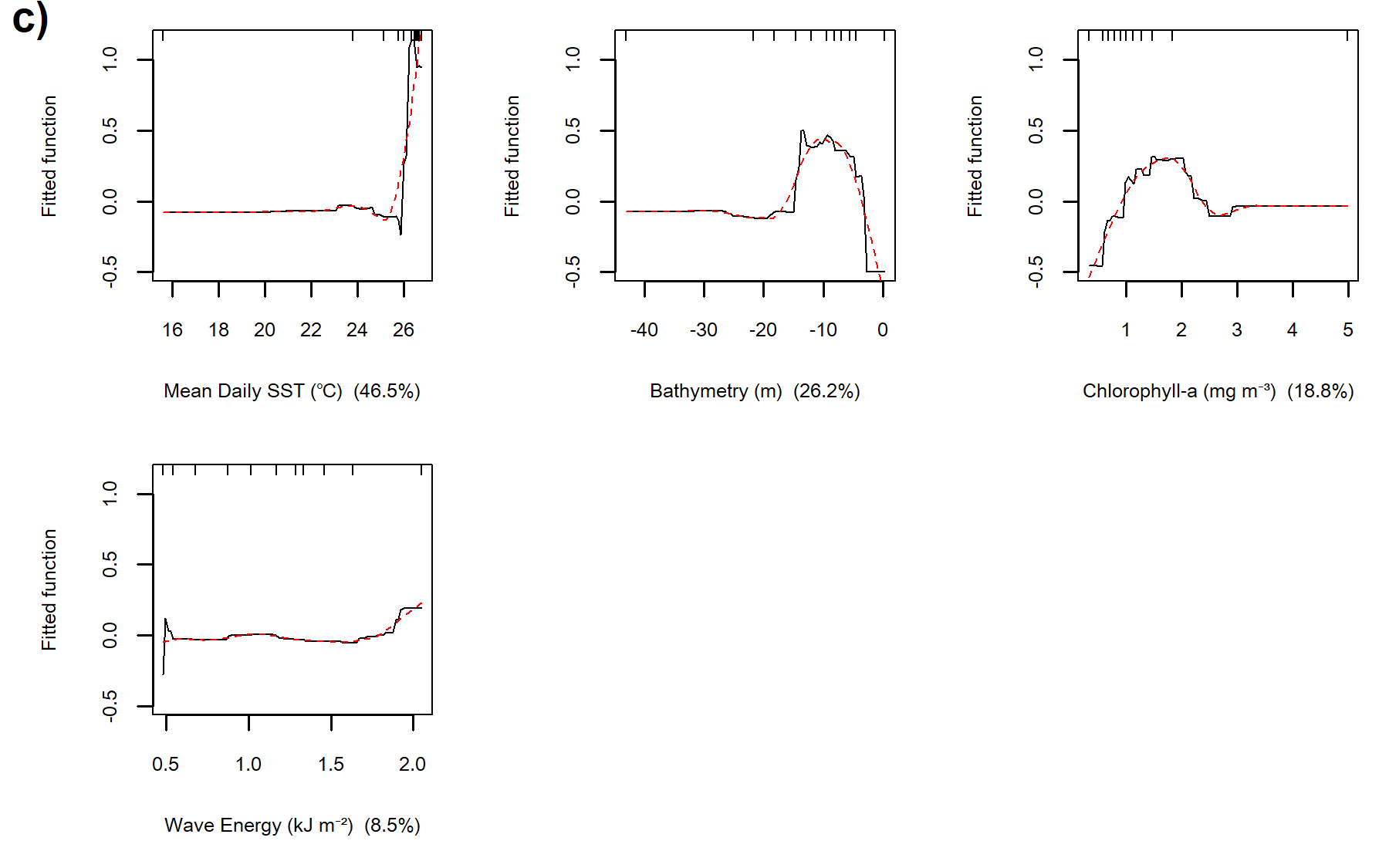


**Figure F.** a) Presence locations of *Dichocoenia stokesi* used to train and test the niche model along the Florida reef tract from 2011–2015. FRRP is the Florida Reef Resiliency Project (FRRP) (yellow crosses, n = 605). Absence locations are not shown. The coral reef layer is a 1 km buffer taken from the Florida Fish and Wildlife Conservation Commission Fish and Wildlife Research Institute’s Unified Florida Reef Tract spatial layer. Basemap: Esri, DigitalGlobe, GeoEye, i-cubed, USDA FSA, USGS, AEX, Getmapping, Aerogrid, IGN, IGP, swisstopo, and the GIS User Community. b) Probability of occurrence of *Dichocoenia stokesi*. Our niche model provides a probability map highlighting where these corals will experience ‘suitable’ environmental conditions for restoration. c) Fitted function plots of the suite of 4 predictor variables that created the most accurate model output for *Dichocoenia stokesi.* The height of the function above or below the “0” mark shows to what degree the suitable habitat is affected, within the range of each variable. The percentage within the parentheses shows the influence of each variable on the model.

**
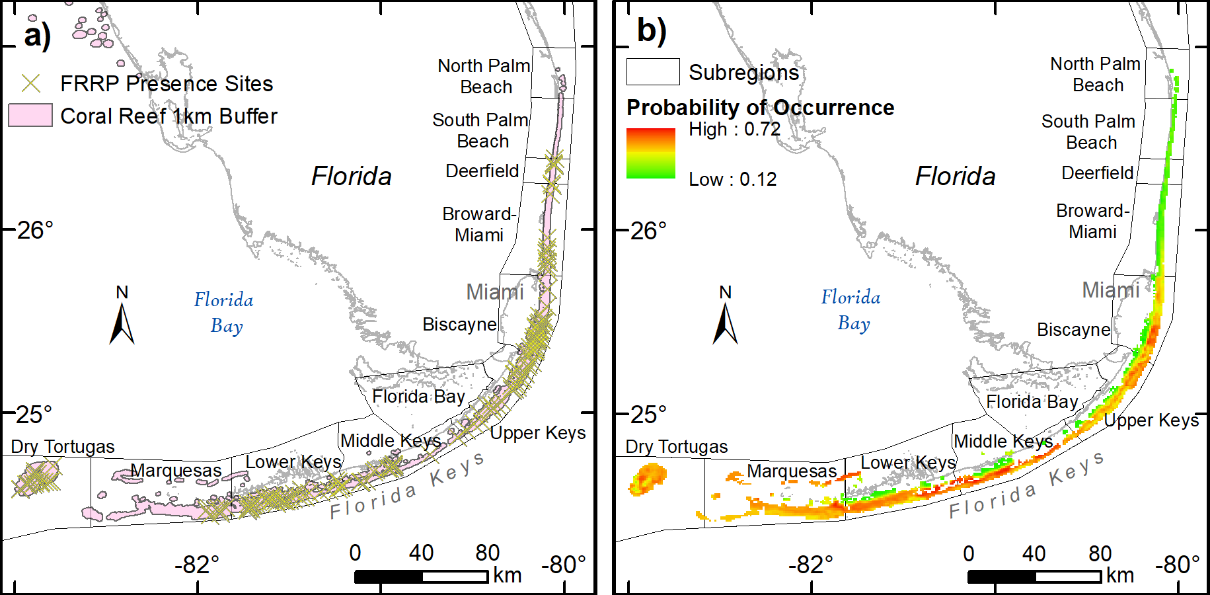
**

**
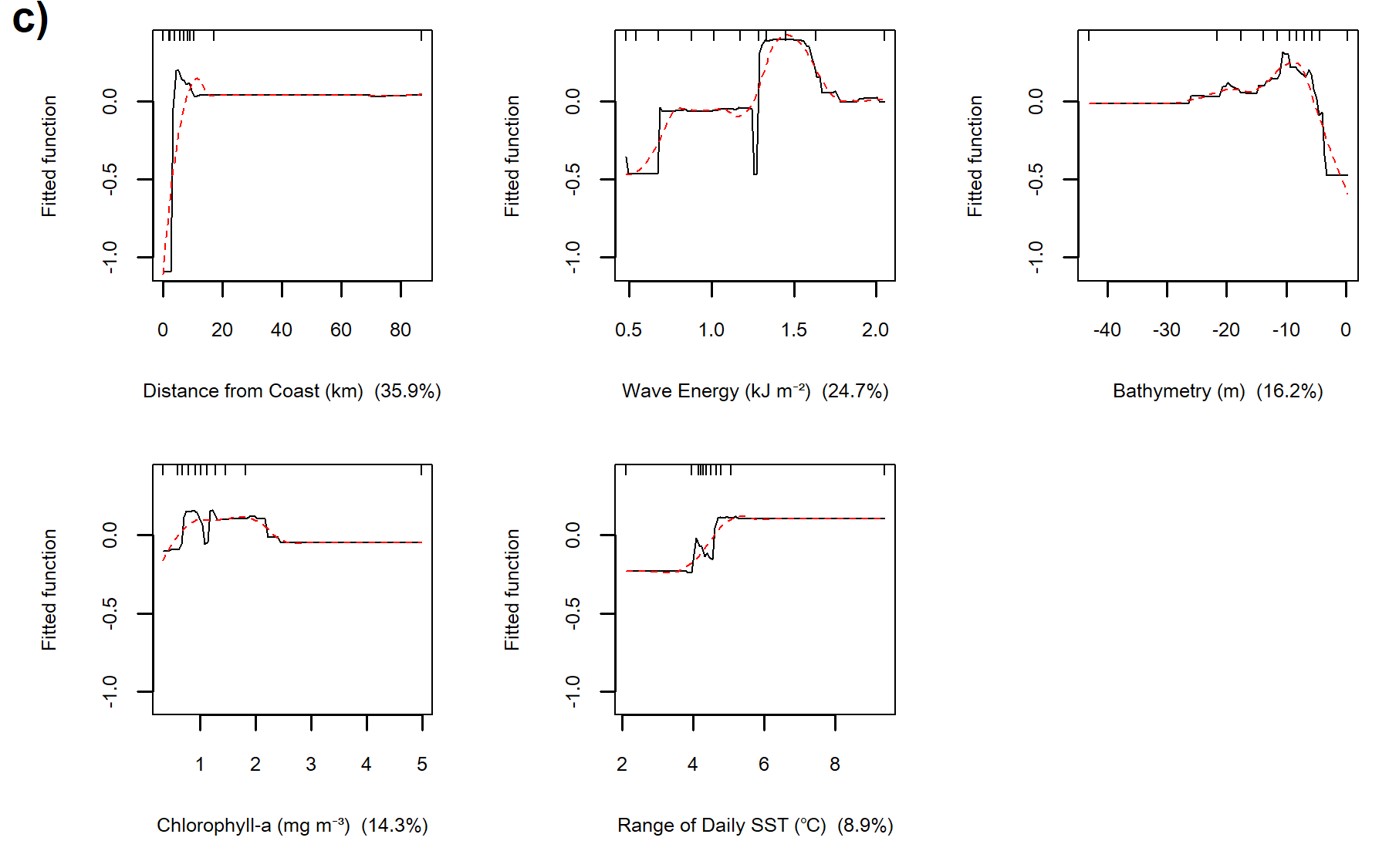
**

**Figure G.** a) Presence locations of *Eusmilia fastigiata* used to train and test the niche model along the Florida reef tract from 2011–2015. FRRP is the Florida Reef Resiliency Project (FRRP) (yellow crosses, n = 168). Absence locations are not shown. The coral reef layer is a 1 km buffer taken from the Florida Fish and Wildlife Conservation Commission Fish and Wildlife Research Institute’s Unified Florida Reef Tract spatial layer. Basemap: Esri, DigitalGlobe, GeoEye, i-cubed, USDA FSA, USGS, AEX, Getmapping, Aerogrid, IGN, IGP, swisstopo, and the GIS User Community. b) Probability of occurrence of *Eusmilia fastigiata*. Our niche model provides a probability map highlighting where these corals will experience ‘suitable’ environmental conditions for restoration. c) Fitted function plots of the suite of 5 predictor variables that created the most accurate model output for *Eusmilia fastigiata.* The height of the function above or below the “0” mark shows to what degree the suitable habitat is affected, within the range of each variable. The percentage within the parentheses shows the influence of each variable on the model.
