# Supplementary material for: Niche space of corals along the Florida reef tract
Source: PLoS One. 2020 Apr 7;15(4):e0231104. doi: 10.1371/journal.pone.0231104 (PMC7138326; doi:10.1371/journal.pone.0231104)
Supplement: S2 File — (DOCX) [file pone.0231104.s002.docx]

**
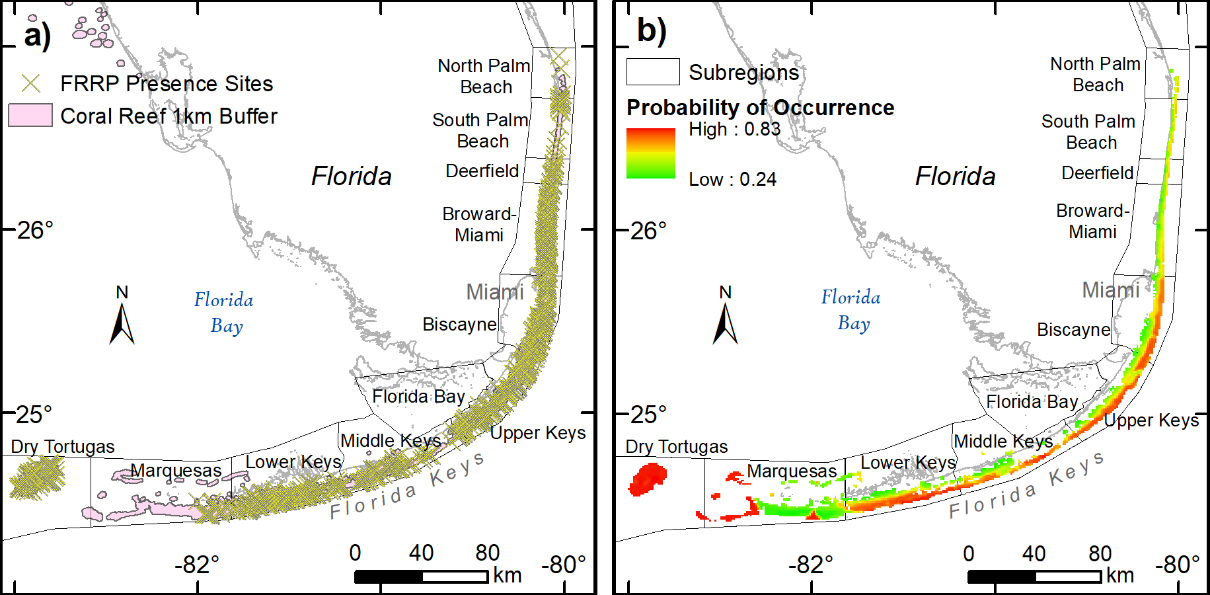
**

**
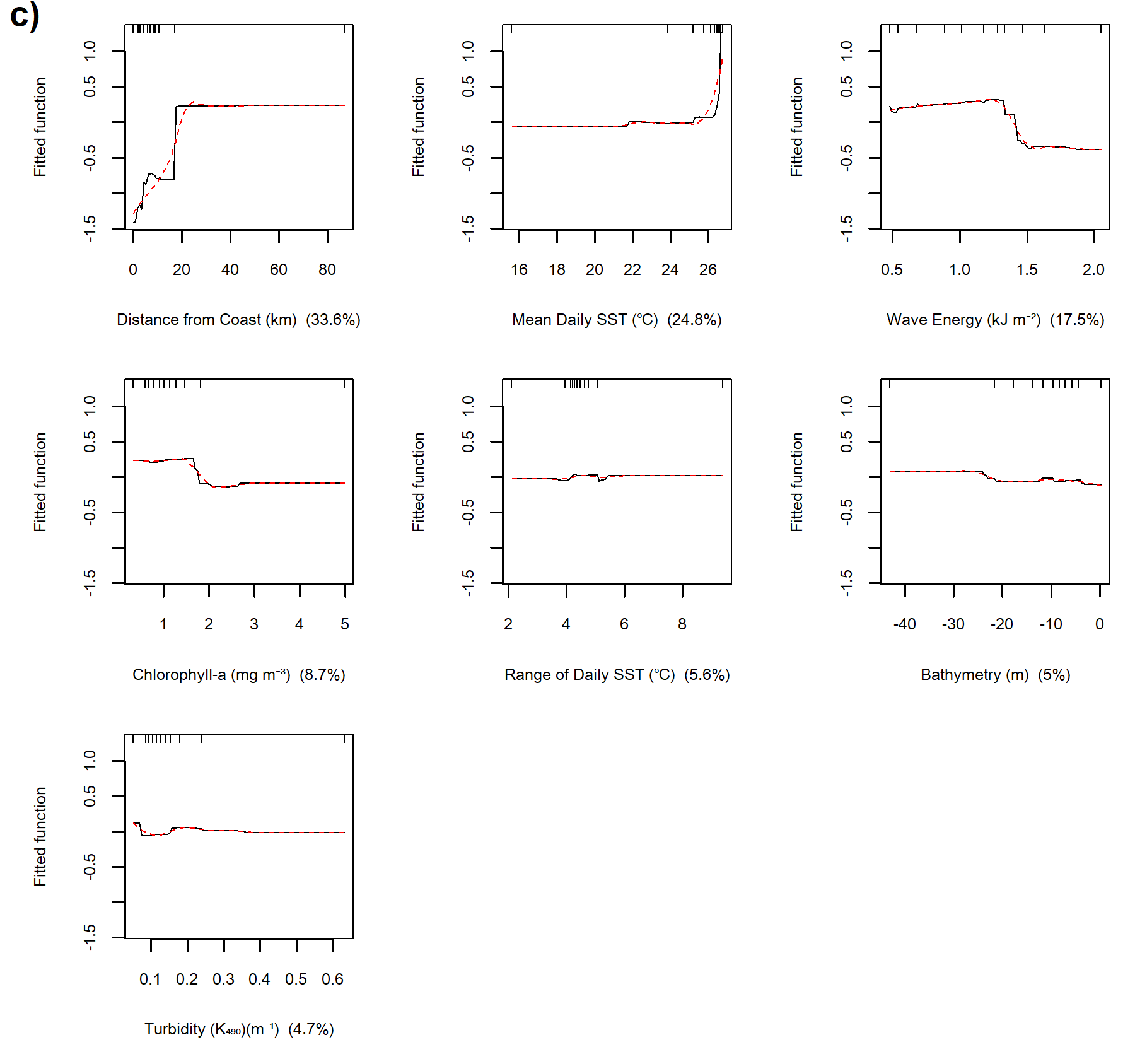
**

**Figure H.** a) Presence locations of *Millepora alcicornis* used to train and test the niche model along the Florida reef tract from 2011–2015. FRRP is the Florida Reef Resiliency Project (FRRP) (yellow crosses, n = 885). Absence locations are not shown. The coral reef layer is a 1 km buffer taken from the Florida Fish and Wildlife Conservation Commission Fish and Wildlife Research Institute’s Unified Florida Reef Tract spatial layer. Basemap: Esri, DigitalGlobe, GeoEye, i-cubed, USDA FSA, USGS, AEX, Getmapping, Aerogrid, IGN, IGP, swisstopo, and the GIS User Community. b) Probability of occurrence of *Millepora alcicornis*. Our niche model provides a probability map highlighting where these corals will experience ‘suitable’ environmental conditions for restoration. c) Fitted function plots of the suite of 7 predictor variables that created the most accurate model output for *Millepora alcicornis.* The height of the function above or below the “0” mark shows to what degree the suitable habitat is affected, within the range of each variable. The percentage within the parentheses shows the influence of each variable on the model.

**
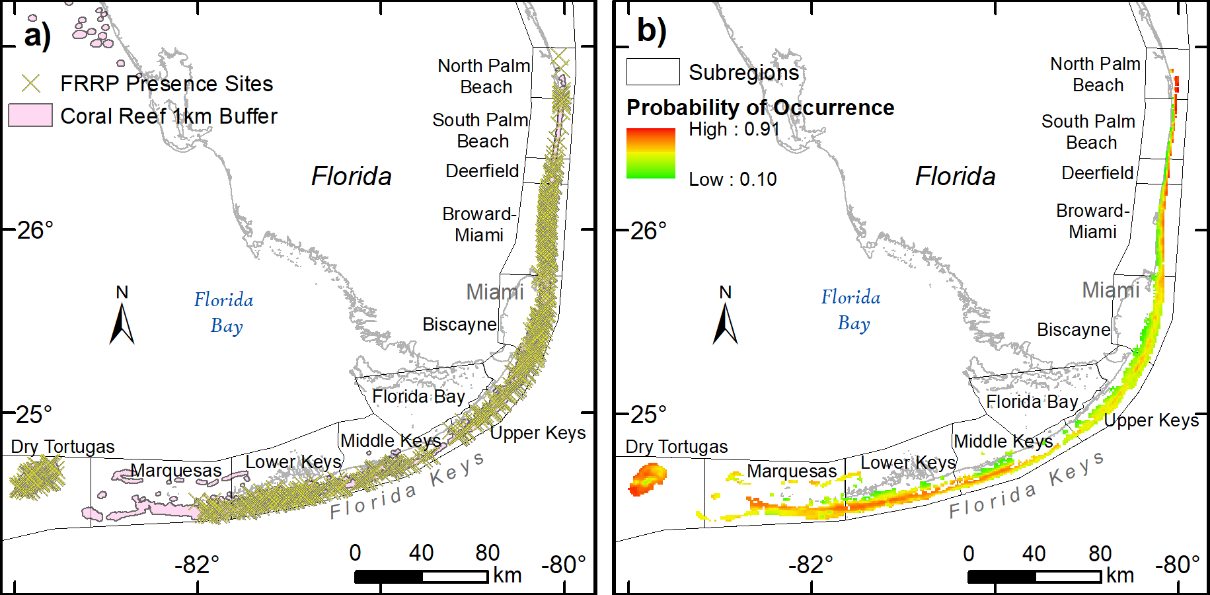
**

**
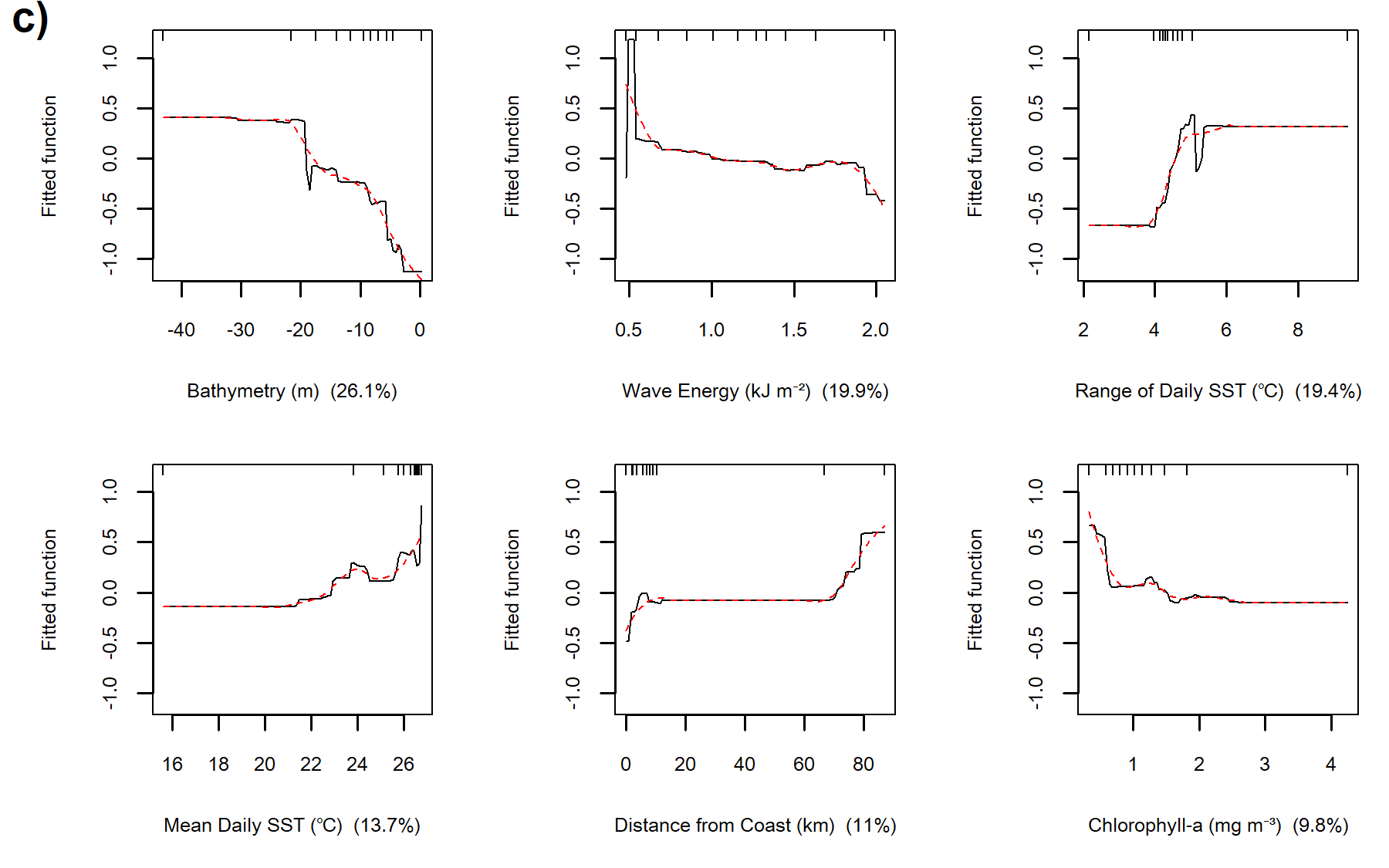
**

**Figure I.** a) Presence locations of *Montastraea cavernosa* used to train and test the niche model along the Florida reef tract from 2011–2015. FRRP is the Florida Reef Resiliency Project (FRRP) (yellow crosses, n = 645). Absence locations are not shown. The coral reef layer is a 1 km buffer taken from the Florida Fish and Wildlife Conservation Commission Fish and Wildlife Research Institute’s Unified Florida Reef Tract spatial layer. Basemap: Esri, DigitalGlobe, GeoEye, i-cubed, USDA FSA, USGS, AEX, Getmapping, Aerogrid, IGN, IGP, swisstopo, and the GIS User Community. b) Probability of occurrence of *Montastraea cavernosa*. Our niche model provides a probability map highlighting where these corals will experience ‘suitable’ environmental conditions for restoration. c) Fitted function plots of the suite of 6 predictor variables that created the most accurate model output for *Montastraea cavernosa.* The height of the function above or below the “0” mark shows to what degree the suitable habitat is affected, within the range of each variable. The percentage within the parentheses shows the influence of each variable on the model.

**
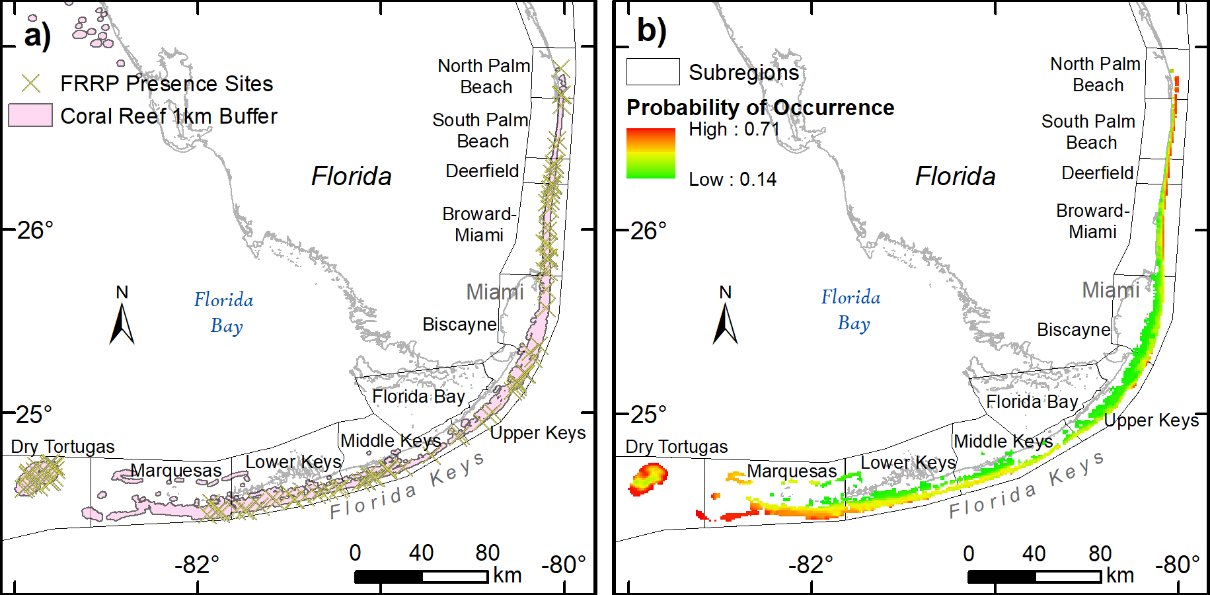
**

**
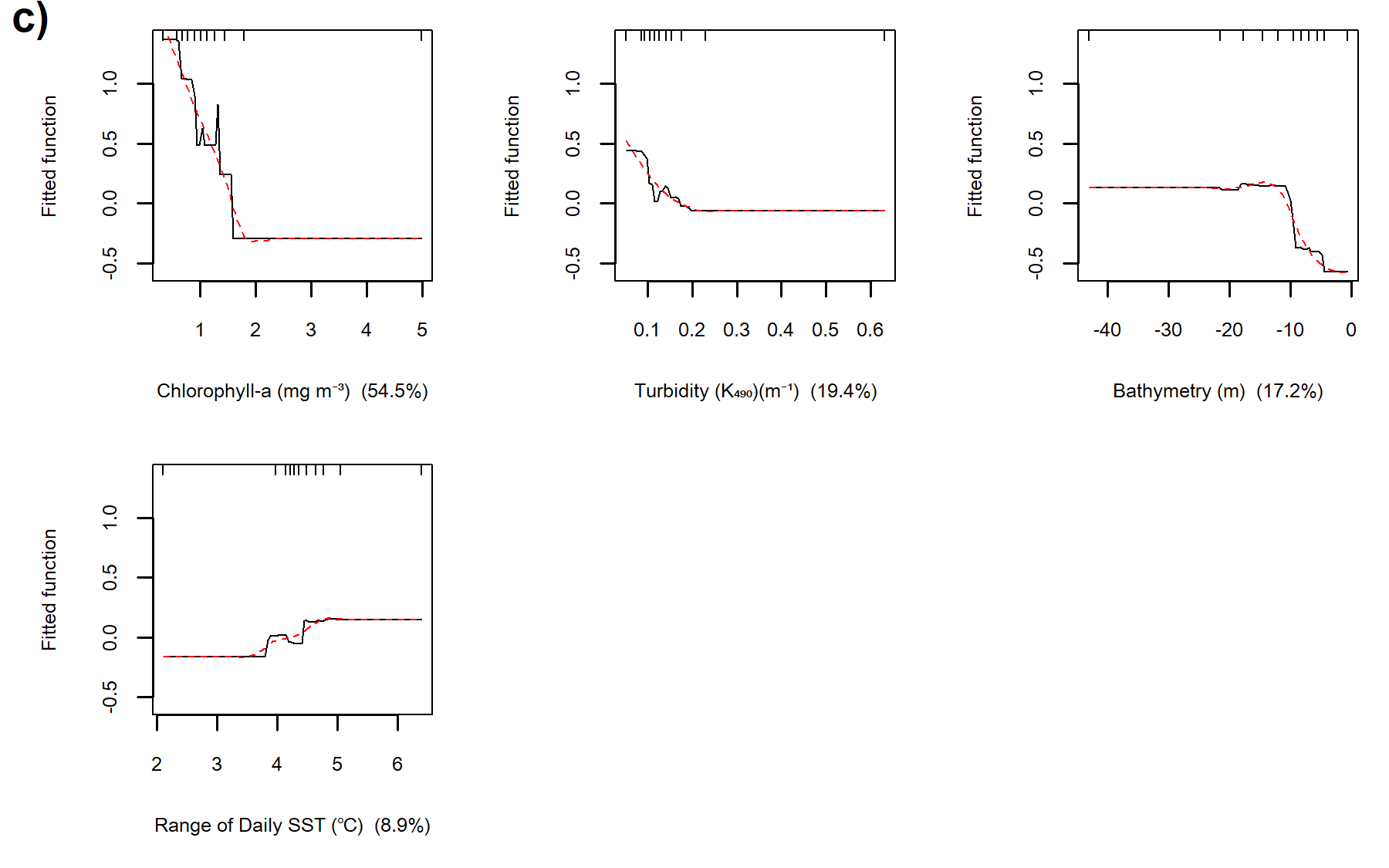
**

**Figure J.** a) Presence locations of *Madracis decactis* used to train and test the niche model along the Florida reef tract from 2011–2015. FRRP is the Florida Reef Resiliency Project (FRRP) (yellow crosses, n = 111). Absence locations are not shown. The coral reef layer is a 1 km buffer taken from the Florida Fish and Wildlife Conservation Commission Fish and Wildlife Research Institute’s Unified Florida Reef Tract spatial layer. Basemap: Esri, DigitalGlobe, GeoEye, i-cubed, USDA FSA, USGS, AEX, Getmapping, Aerogrid, IGN, IGP, swisstopo, and the GIS User Community. b) Probability of occurrence of *Madracis decactis*. Our niche model provides a probability map highlighting where these corals will experience ‘suitable’ environmental conditions for restoration. c) Fitted function plots of the suite of 4 predictor variables that created the most accurate model output for *Madracis decactis.* The height of the function above or below the “0” mark shows to what degree the suitable habitat is affected, within the range of each variable. The percentage within the parentheses shows the influence of each variable on the model.

**
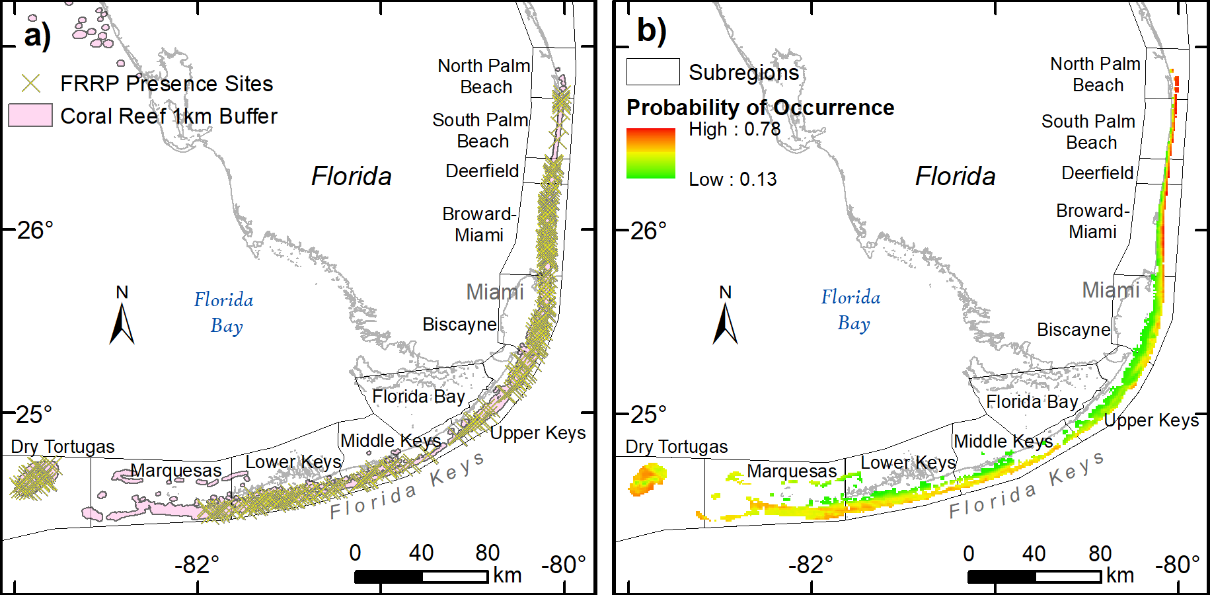
**

**
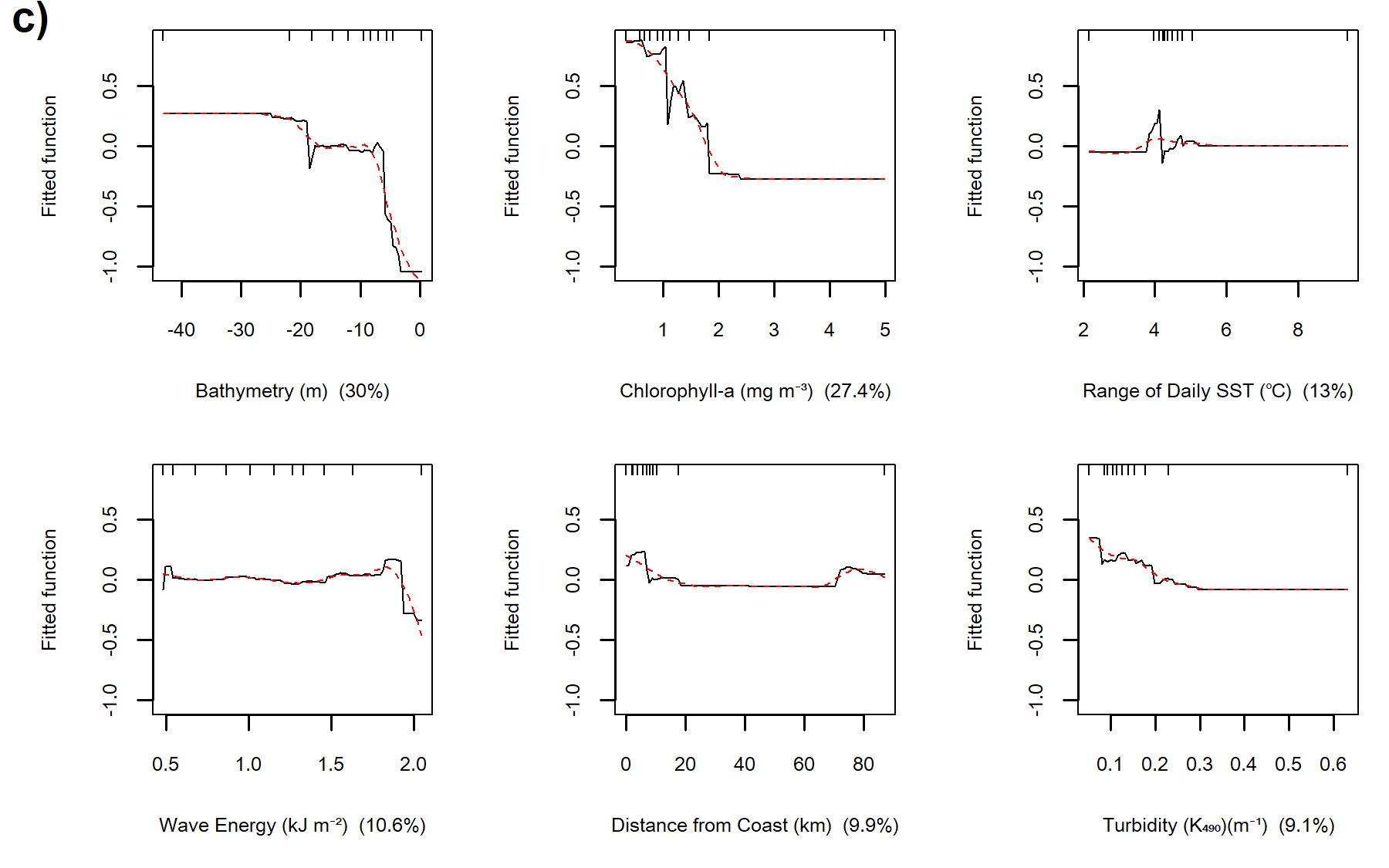
**

**Figure K.** a) Presence locations of *Meandrina meandrites* used to train and test the niche model along the Florida reef tract from 2011–2015. FRRP is the Florida Reef Resiliency Project (FRRP) (yellow crosses, n = 317). Absence locations are not shown. The coral reef layer is a 1 km buffer taken from the Florida Fish and Wildlife Conservation Commission Fish and Wildlife Research Institute’s Unified Florida Reef Tract spatial layer. Basemap: Esri, DigitalGlobe, GeoEye, i-cubed, USDA FSA, USGS, AEX, Getmapping, Aerogrid, IGN, IGP, swisstopo, and the GIS User Community. b) Probability of occurrence of *Meandrina meandrites*. Our niche model provides a probability map highlighting where these corals will experience ‘suitable’ environmental conditions for restoration. c) Fitted function plots of the suite of 6 predictor variables that created the most accurate model output for *Meandrina meandrites.* The height of the function above or below the “0” mark shows to what degree the suitable habitat is affected, within the range of each variable. The percentage within the parentheses shows the influence of each variable on the model.


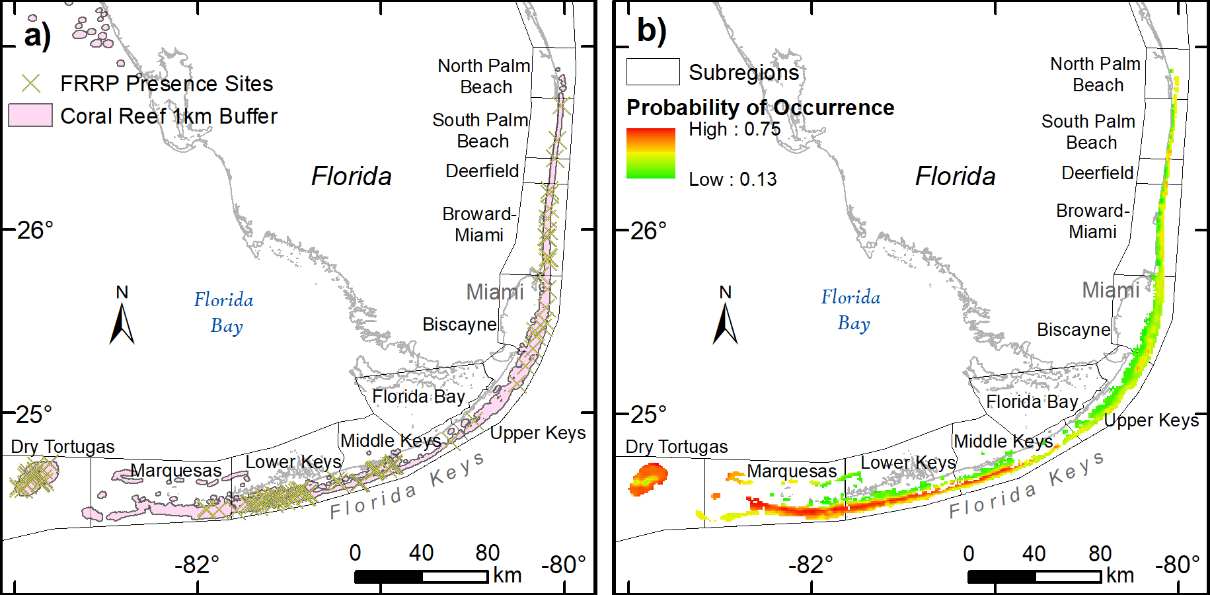


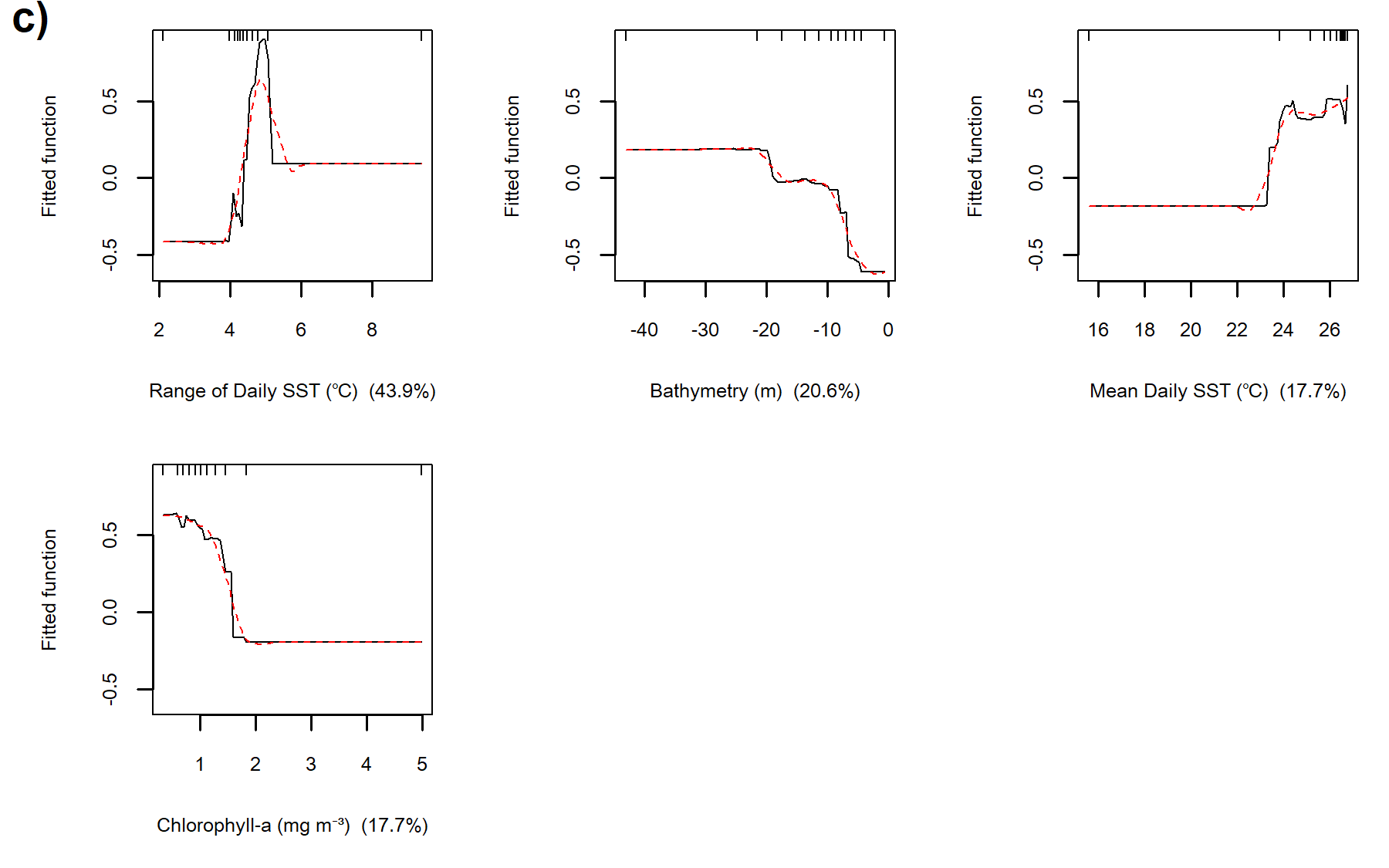


**Figure L.** a) Presence locations of *Mycetophyllia* sp. used to train and test the niche model along the Florida reef tract from 2011–2015. FRRP is the Florida Reef Resiliency Project (FRRP) (yellow crosses, n = 106). Absence locations are not shown. The coral reef layer is a 1 km buffer taken from the Florida Fish and Wildlife Conservation Commission Fish and Wildlife Research Institute’s Unified Florida Reef Tract spatial layer. Basemap: Esri, DigitalGlobe, GeoEye, i-cubed, USDA FSA, USGS, AEX, Getmapping, Aerogrid, IGN, IGP, swisstopo, and the GIS User Community. b) Probability of occurrence of *Mycetophyllia* sp. Our niche model provides a probability map highlighting where these corals will experience ‘suitable’ environmental conditions for restoration. c) Fitted function plots of the suite of 4 predictor variables that created the most accurate model output for *Mycetophyllia* sp*.* The height of the function above or below the “0” mark shows to what degree the suitable habitat is affected, within the range of each variable. The percentage within the parentheses shows the influence of each variable on the model.


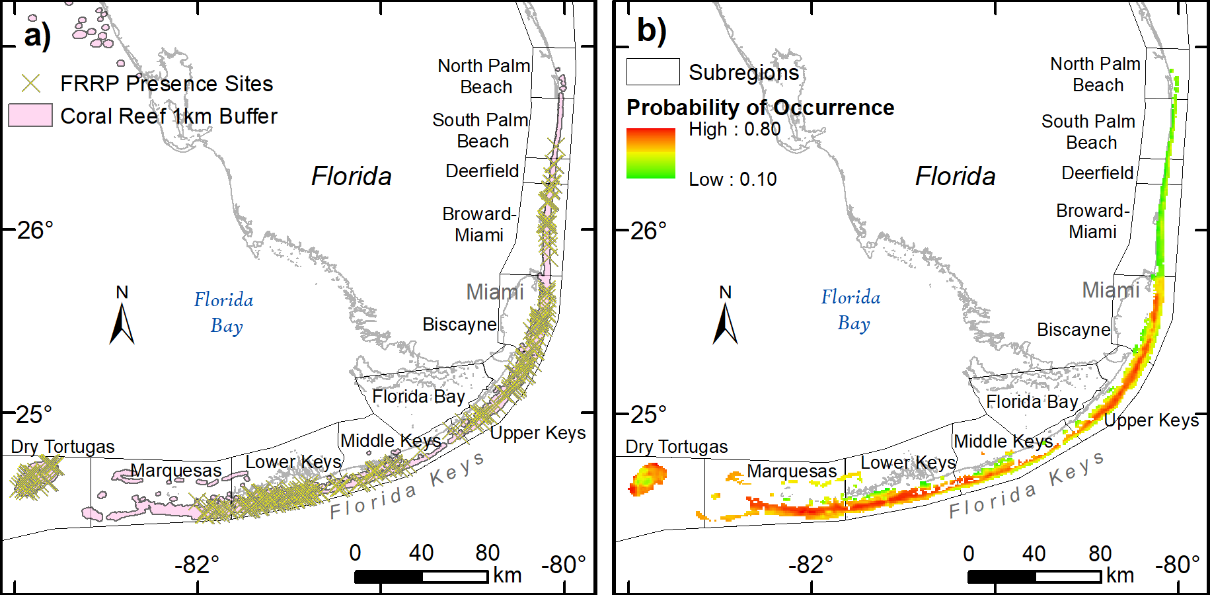


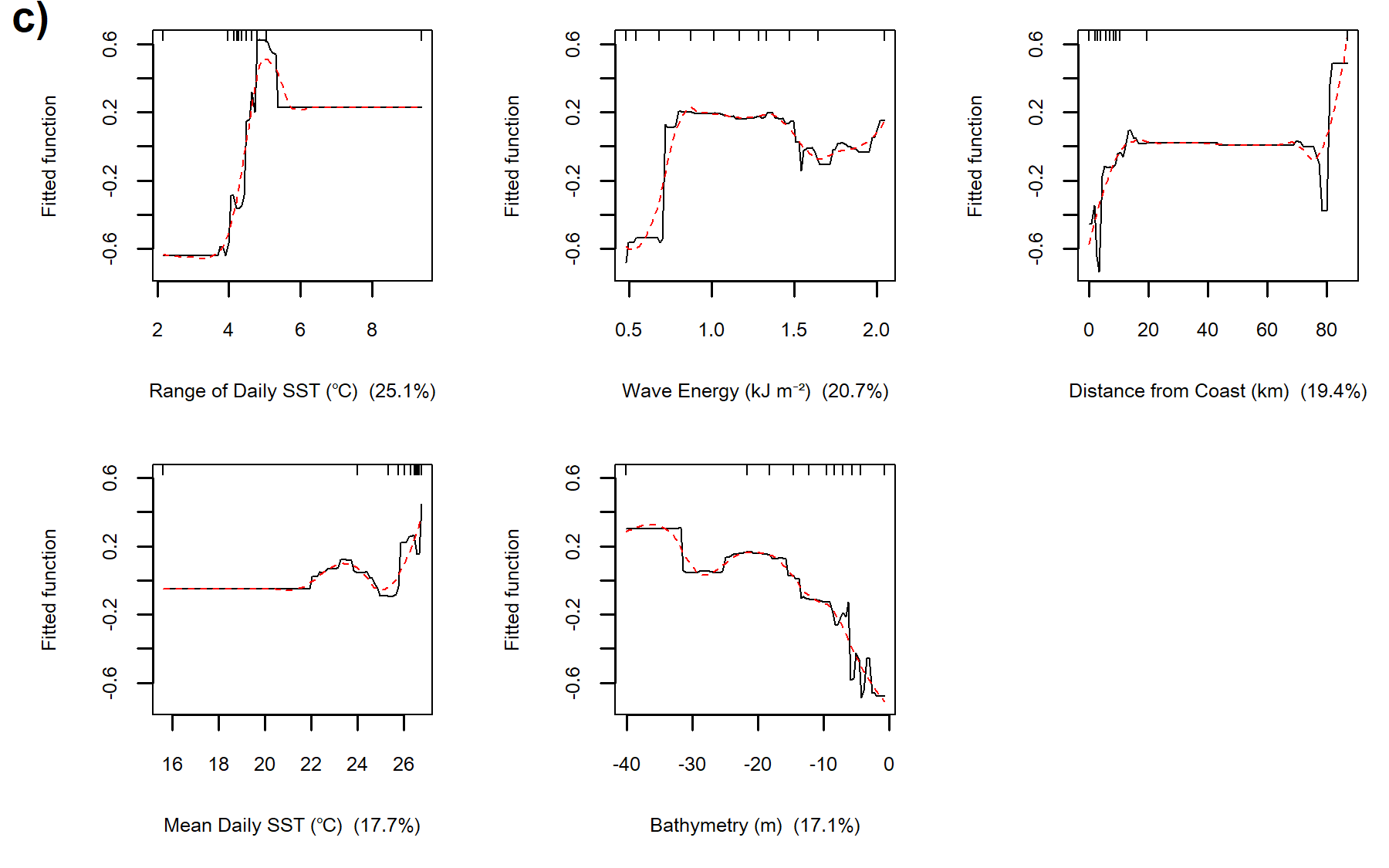


**Figure M.** a) Presence locations of *Orbicella faveolata* used to train and test the niche model along the Florida reef tract from 2011–2015. FRRP is the Florida Reef Resiliency Project (FRRP) (yellow crosses, n = 302). Absence locations are not shown. The coral reef layer is a 1 km buffer taken from the Florida Fish and Wildlife Conservation Commission Fish and Wildlife Research Institute’s Unified Florida Reef Tract spatial layer. Basemap: Esri, DigitalGlobe, GeoEye, i-cubed, USDA FSA, USGS, AEX, Getmapping, Aerogrid, IGN, IGP, swisstopo, and the GIS User Community. b) Probability of occurrence of *Orbicella faveolata*. Our niche model provides a probability map highlighting where these corals will experience ‘suitable’ environmental conditions for restoration. c) Fitted function plots of the suite of 5 predictor variables that created the most accurate model output for *Orbicella faveolata.* The height of the function above or below the “0” mark shows to what degree the suitable habitat is affected, within the range of each variable. The percentage within the parentheses shows the influence of each variable on the model.


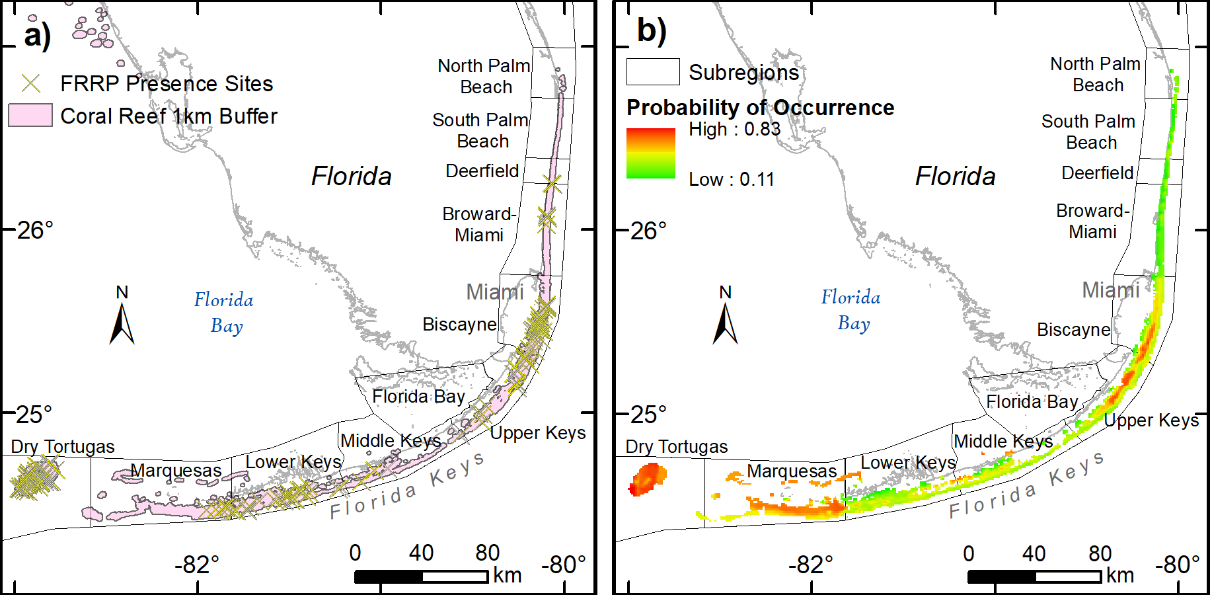


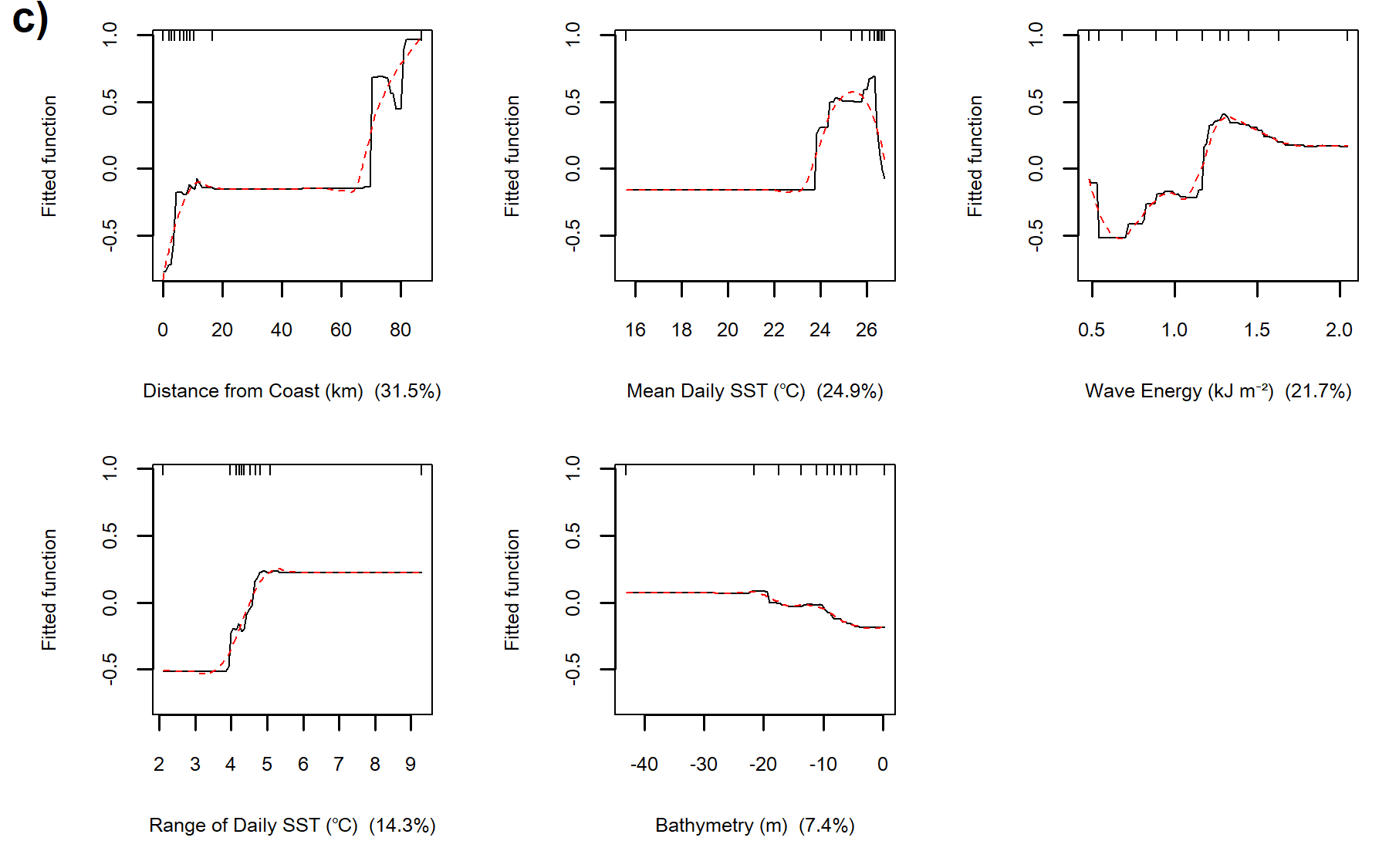


**Figure N.** a) Presence locations of *Orbicella franksi* used to train and test the niche model along the Florida reef tract from 2011–2015. FRRP is the Florida Reef Resiliency Project (FRRP) (yellow crosses, n = 124). Absence locations are not shown. The coral reef layer is a 1 km buffer taken from the Florida Fish and Wildlife Conservation Commission Fish and Wildlife Research Institute’s Unified Florida Reef Tract spatial layer. Basemap: Esri, DigitalGlobe, GeoEye, i-cubed, USDA FSA, USGS, AEX, Getmapping, Aerogrid, IGN, IGP, swisstopo, and the GIS User Community. b) Probability of occurrence of *Orbicella franksi*. Our niche model provides a probability map highlighting where these corals will experience ‘suitable’ environmental conditions for restoration. c) Fitted function plots of the suite of 5 predictor variables that created the most accurate model output for *Orbicella franksi.* The height of the function above or below the “0” mark shows to what degree the suitable habitat is affected, within the range of each variable. The percentage within the parentheses shows the influence of each variable on the model.
