# Supplementary material for: Niche space of corals along the Florida reef tract
Source: PLoS One. 2020 Apr 7;15(4):e0231104. doi: 10.1371/journal.pone.0231104 (PMC7138326; doi:10.1371/journal.pone.0231104)
Supplement: S3 File — (DOCX) [file pone.0231104.s003.docx]

**
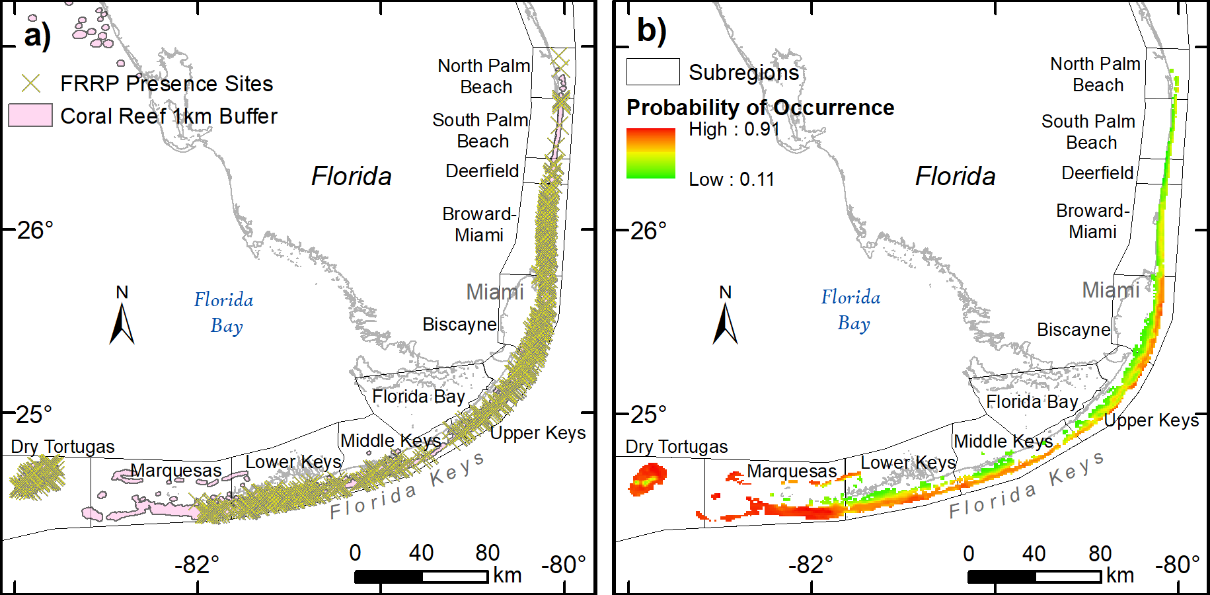
**

**
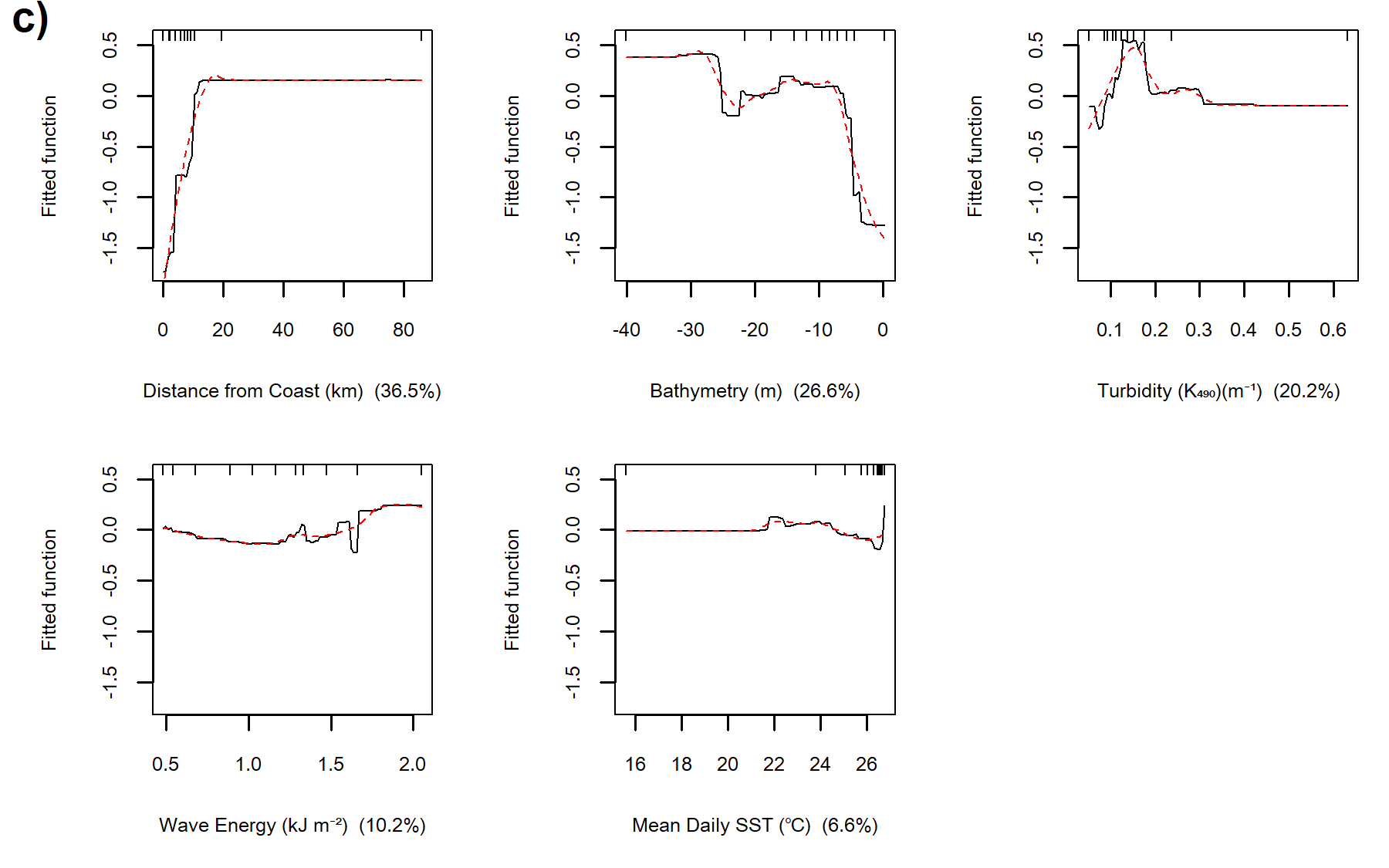
**

**Figure O.** a) Presence locations of *Porites astreoides* used to train and test the niche model along the Florida reef tract from 2011–2015. FRRP is the Florida Reef Resiliency Project (FRRP) (yellow crosses, n = 806). Absence locations are not shown. The coral reef layer is a 1 km buffer taken from the Florida Fish and Wildlife Conservation Commission Fish and Wildlife Research Institute’s Unified Florida Reef Tract spatial layer. Basemap: Esri, DigitalGlobe, GeoEye, i-cubed, USDA FSA, USGS, AEX, Getmapping, Aerogrid, IGN, IGP, swisstopo, and the GIS User Community. b) Probability of occurrence of *Porites astreoides*. Our niche model provides a probability map highlighting where these corals will experience ‘suitable’ environmental conditions for restoration. c) Fitted function plots of the suite of 5 predictor variables that created the most accurate model output for *Porites astreoides.* The height of the function above or below the “0” mark shows to what degree the suitable habitat is affected, within the range of each variable. The percentage within the parentheses shows the influence of each variable on the model.

**
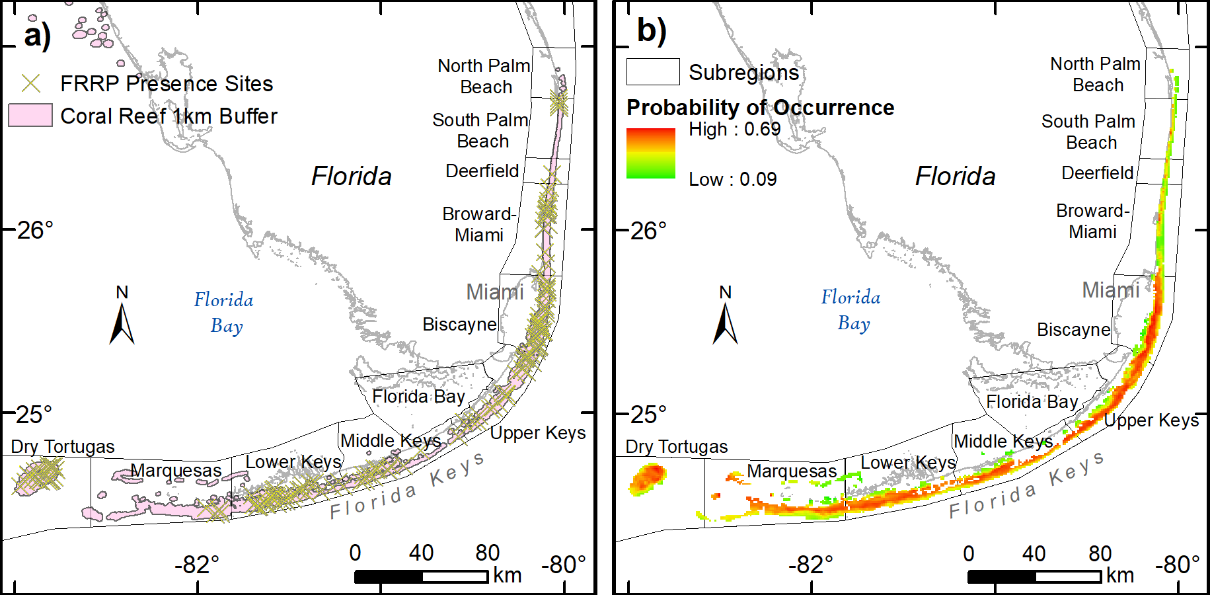
**

**
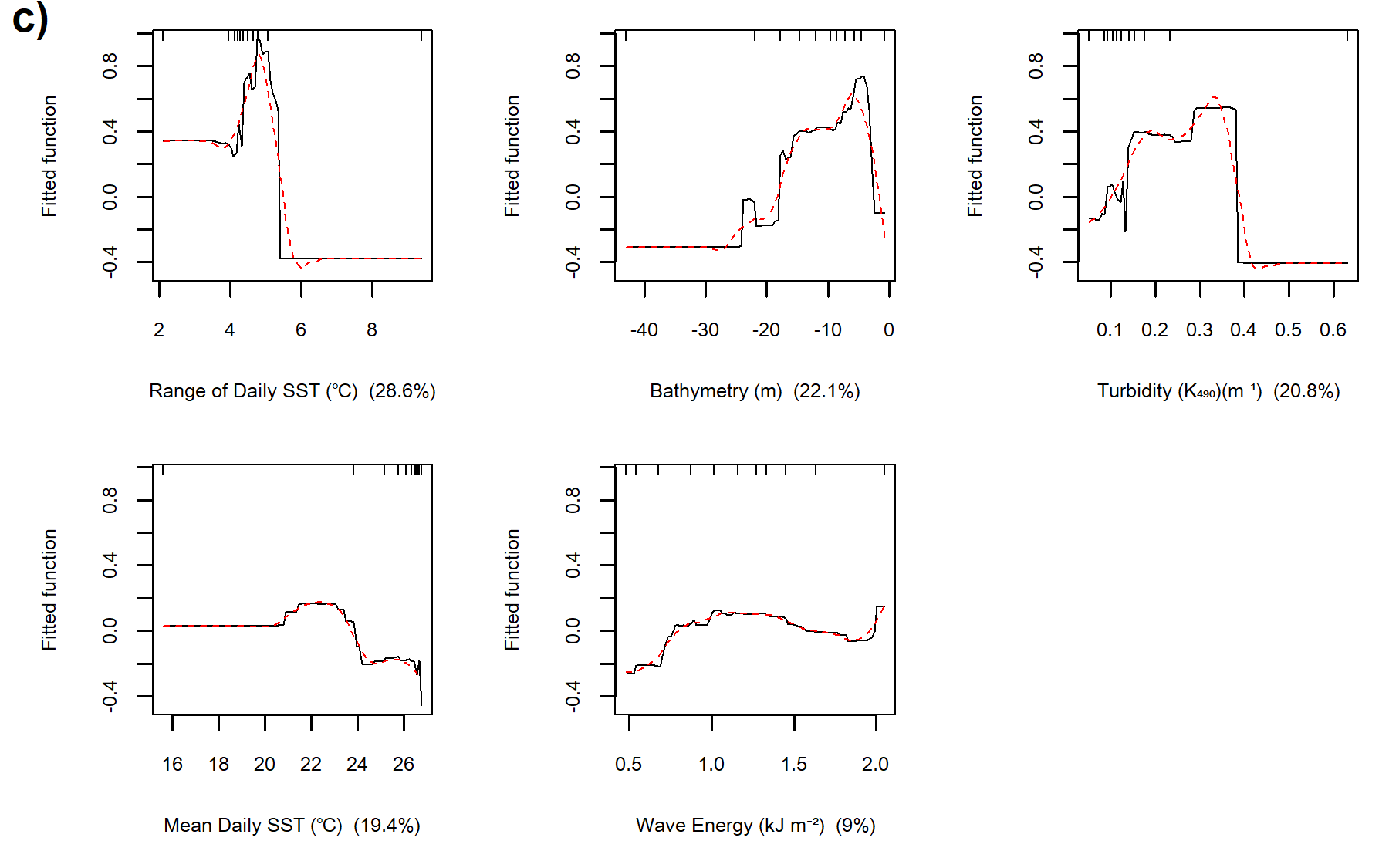
**

**Figure P.** a) Presence locations of *Pseudodiploria clivosa* used to train and test the niche model along the Florida reef tract from 2011–2015. FRRP is the Florida Reef Resiliency Project (FRRP) (yellow crosses, n = 155). Absence locations are not shown. The coral reef layer is a 1 km buffer taken from the Florida Fish and Wildlife Conservation Commission Fish and Wildlife Research Institute’s Unified Florida Reef Tract spatial layer. Basemap: Esri, DigitalGlobe, GeoEye, i-cubed, USDA FSA, USGS, AEX, Getmapping, Aerogrid, IGN, IGP, swisstopo, and the GIS User Community. b) Probability of occurrence of *Pseudodiploria clivosa*. Our niche model provides a probability map highlighting where these corals will experience ‘suitable’ environmental conditions for restoration. c) Fitted function plots of the suite of 5 predictor variables that created the most accurate model output for *Pseudodiploria clivosa.* The height of the function above or below the “0” mark shows to what degree the suitable habitat is affected, within the range of each variable. The percentage within the parentheses shows the influence of each variable on the model.


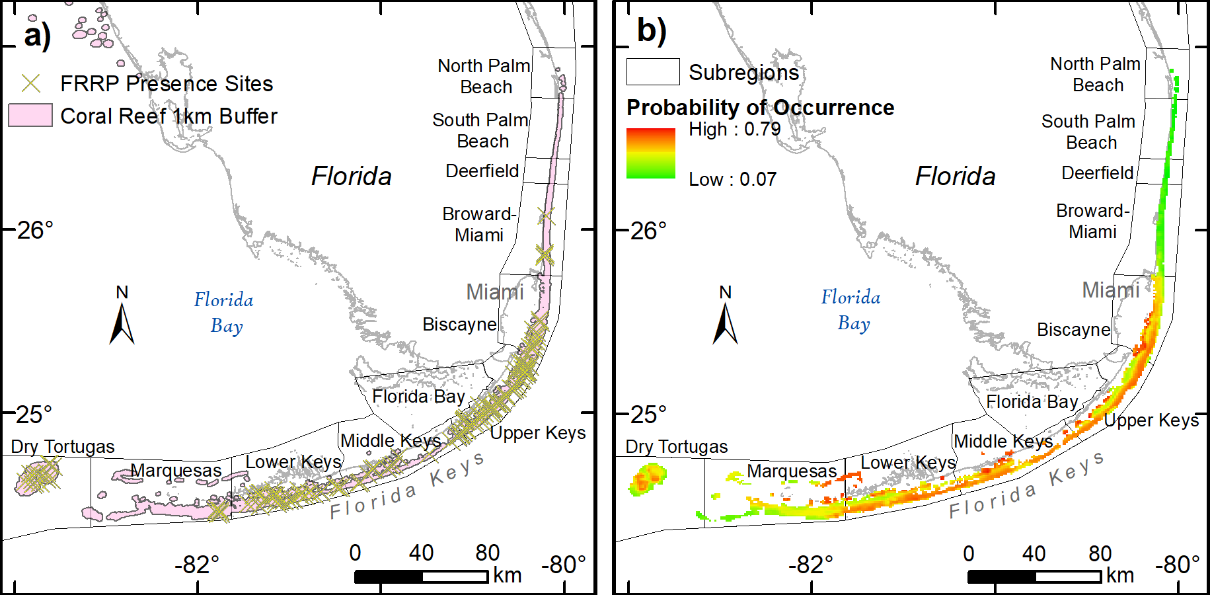


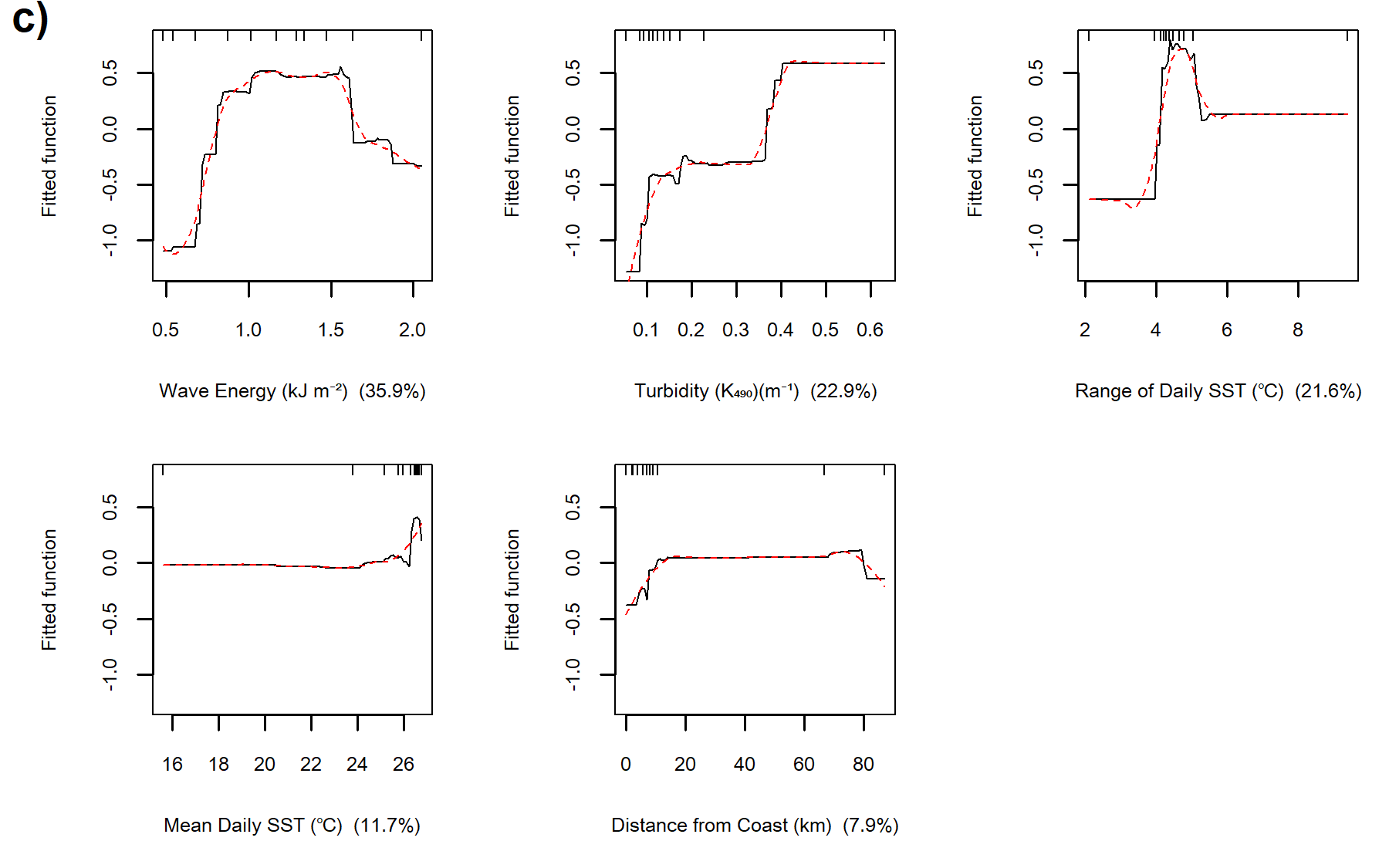


**Figure Q.** a) Presence locations of *Porites divaricata* used to train and test the niche model along the Florida reef tract from 2011–2015. FRRP is the Florida Reef Resiliency Project (FRRP) (yellow crosses, n = 152). Absence locations are not shown. The coral reef layer is a 1 km buffer taken from the Florida Fish and Wildlife Conservation Commission Fish and Wildlife Research Institute’s Unified Florida Reef Tract spatial layer. Basemap: Esri, DigitalGlobe, GeoEye, i-cubed, USDA FSA, USGS, AEX, Getmapping, Aerogrid, IGN, IGP, swisstopo, and the GIS User Community. b) Probability of occurrence of *Porites divaricata*. Our niche model provides a probability map highlighting where these corals will experience ‘suitable’ environmental conditions for restoration. c) Fitted function plots of the suite of 5 predictor variables that created the most accurate model output for *Porites divaricata.* The height of the function above or below the “0” mark shows to what degree the suitable habitat is affected, within the range of each variable. The percentage within the parentheses shows the influence of each variable on the model.

**
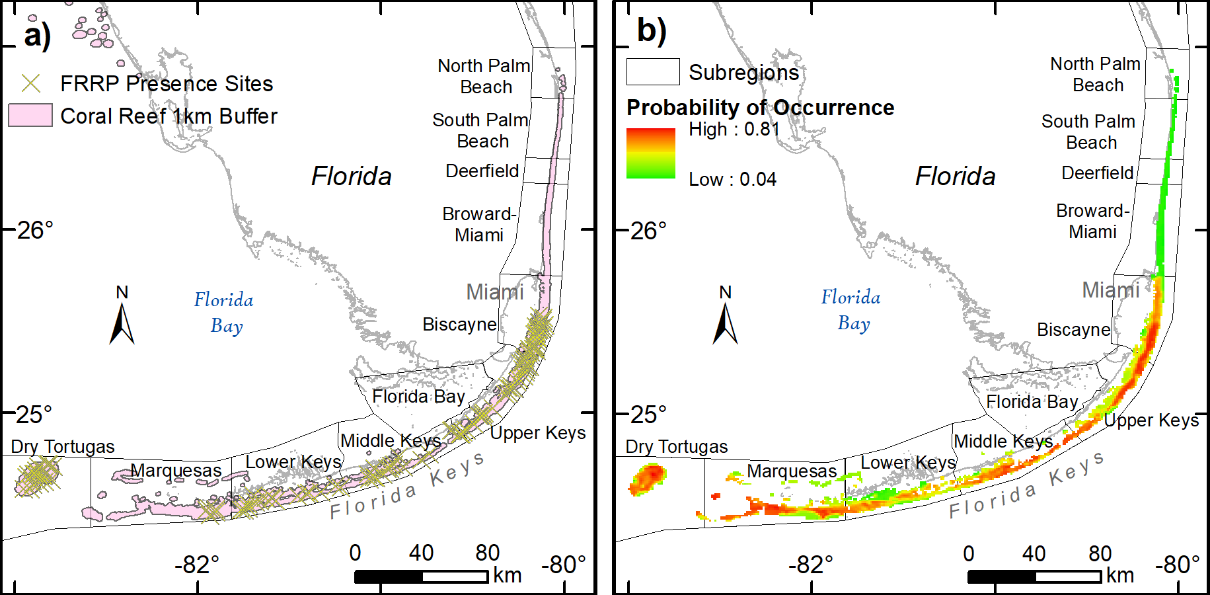
**

**
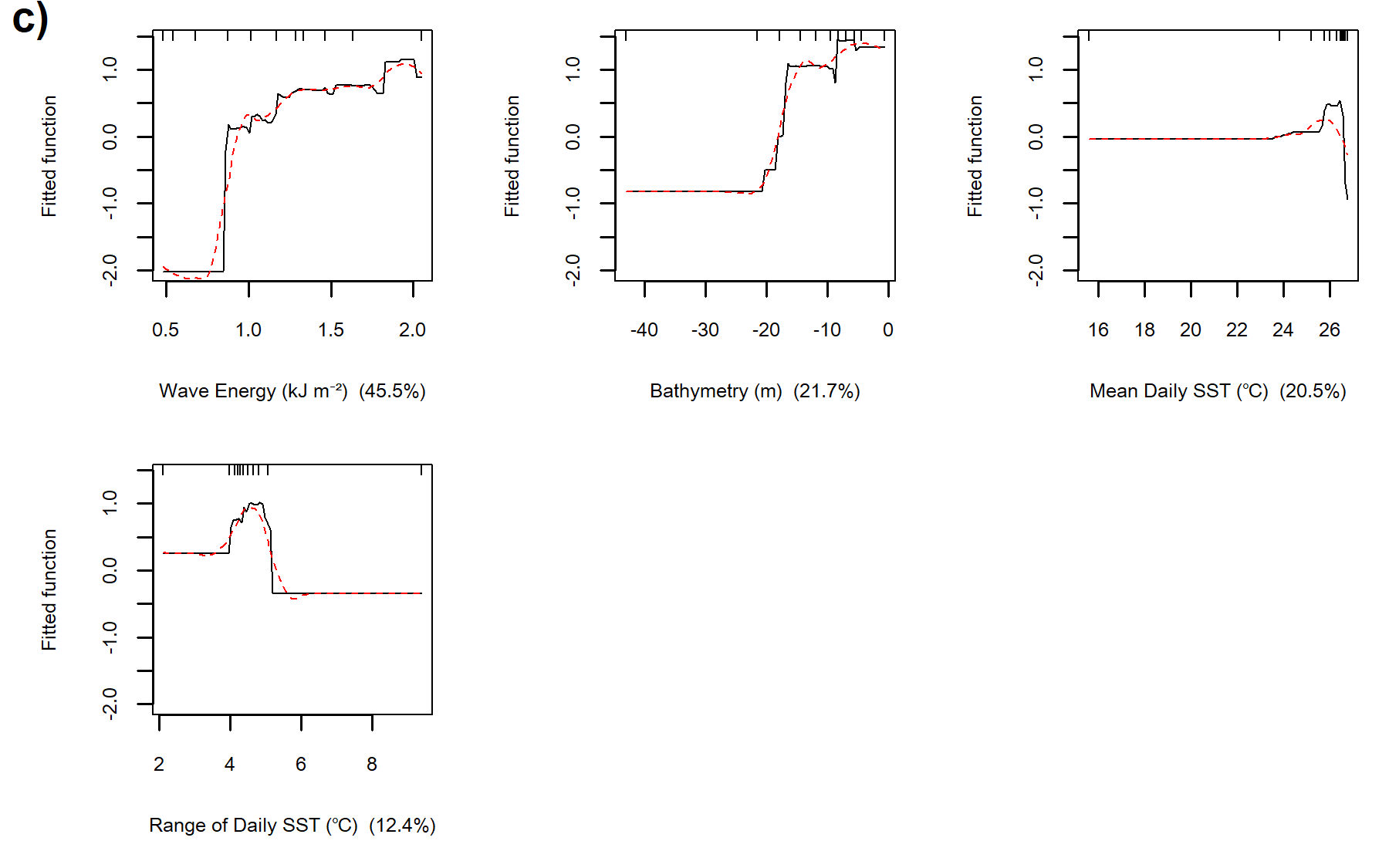
**

**Figure R.** a) Presence locations of *Porites furcata* used to train and test the niche model along the Florida reef tract from 2011–2015. FRRP is the Florida Reef Resiliency Project (FRRP) (yellow crosses, n = 128). Absence locations are not shown. The coral reef layer is a 1 km buffer taken from the Florida Fish and Wildlife Conservation Commission Fish and Wildlife Research Institute’s Unified Florida Reef Tract spatial layer. Basemap: Esri, DigitalGlobe, GeoEye, i-cubed, USDA FSA, USGS, AEX, Getmapping, Aerogrid, IGN, IGP, swisstopo, and the GIS User Community. b) Probability of occurrence of *Porites furcata*. Our niche model provides a probability map highlighting where these corals will experience ‘suitable’ environmental conditions for restoration. c) Fitted function plots of the suite of 4 predictor variables that created the most accurate model output for *Porites furcata.* The height of the function above or below the “0” mark shows to what degree the suitable habitat is affected, within the range of each variable. The percentage within the parentheses shows the influence of each variable on the model.

**
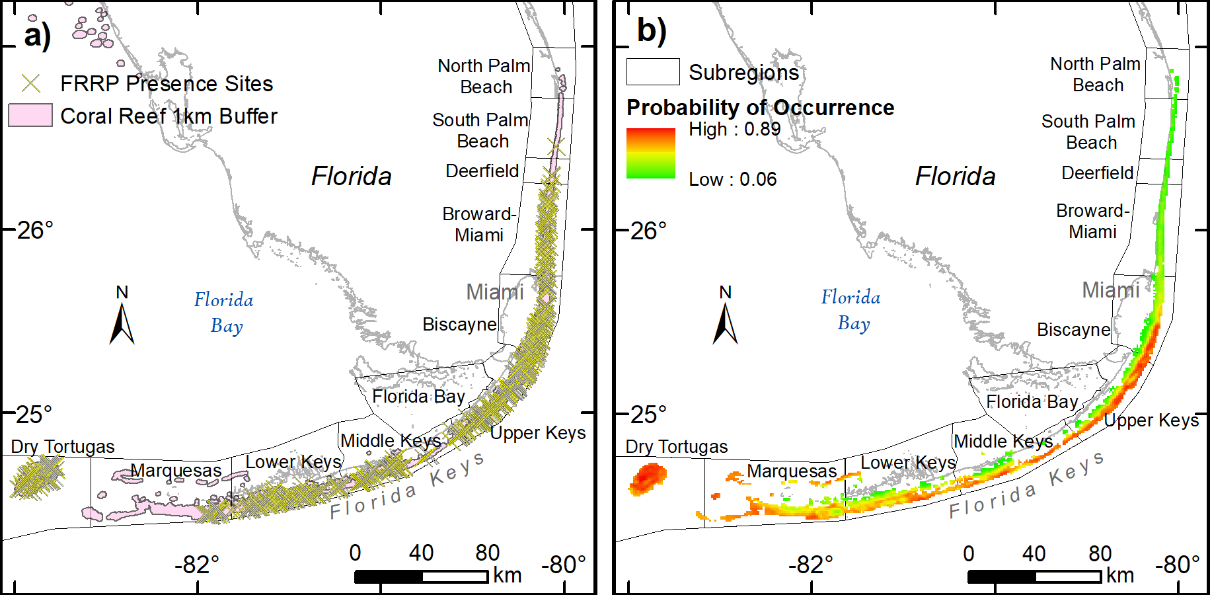
**

**
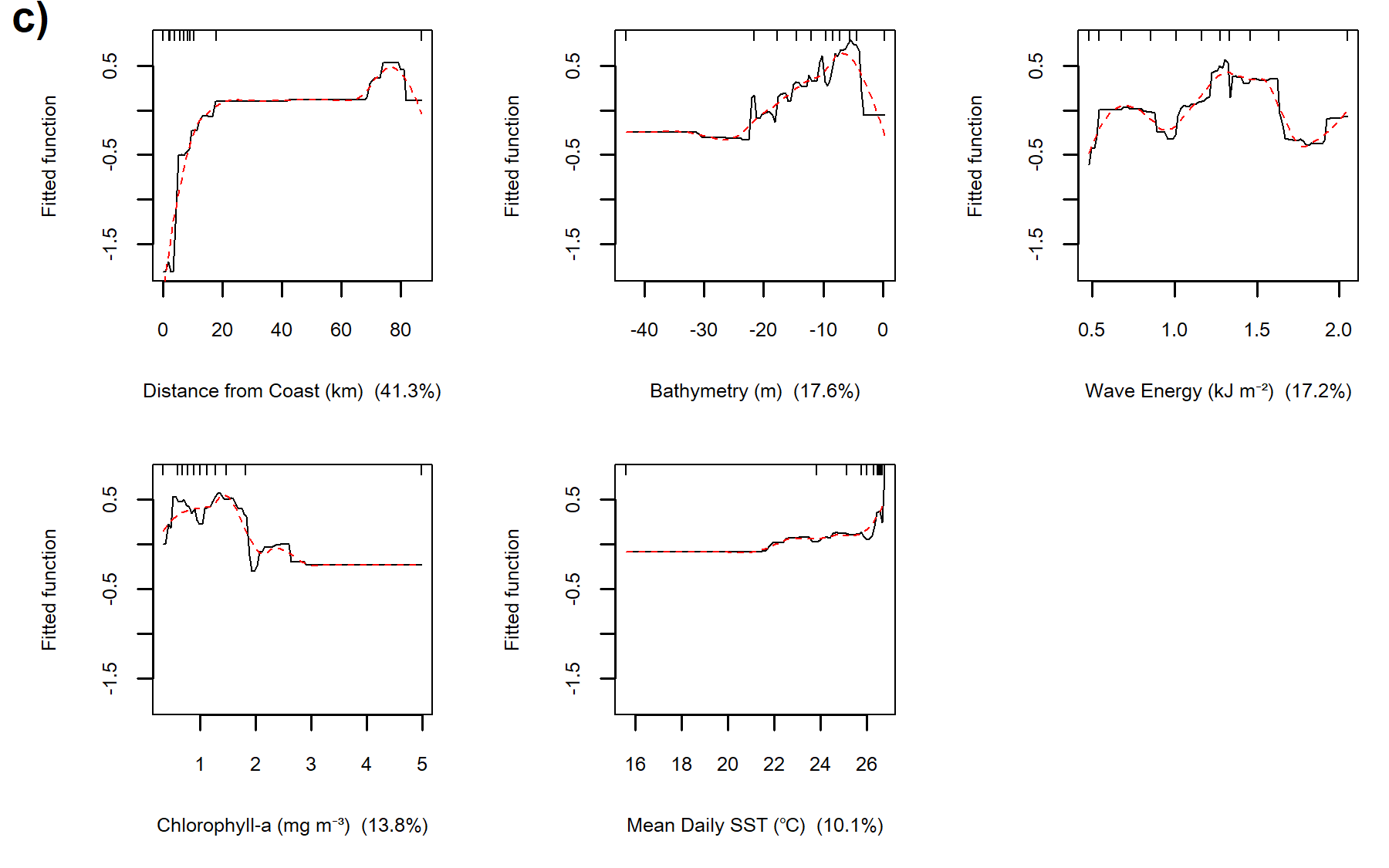
**

**Figure S.** a) Presence locations of *Porites porites* used to train and test the niche model along the Florida reef tract from 2011–2015. FRRP is the Florida Reef Resiliency Project (FRRP) (yellow crosses, n = 549). Absence locations are not shown. The coral reef layer is a 1 km buffer taken from the Florida Fish and Wildlife Conservation Commission Fish and Wildlife Research Institute’s Unified Florida Reef Tract spatial layer. Basemap: Esri, DigitalGlobe, GeoEye, i-cubed, USDA FSA, USGS, AEX, Getmapping, Aerogrid, IGN, IGP, swisstopo, and the GIS User Community. b) Probability of occurrence of *Porites porites*. Our niche model provides a probability map highlighting where these corals will experience ‘suitable’ environmental conditions for restoration. c) Fitted function plots of the suite of 5 predictor variables that created the most accurate model output for *Porites porites.* The height of the function above or below the “0” mark shows to what degree the suitable habitat is affected, within the range of each variable. The percentage within the parentheses shows the influence of each variable on the model.

**
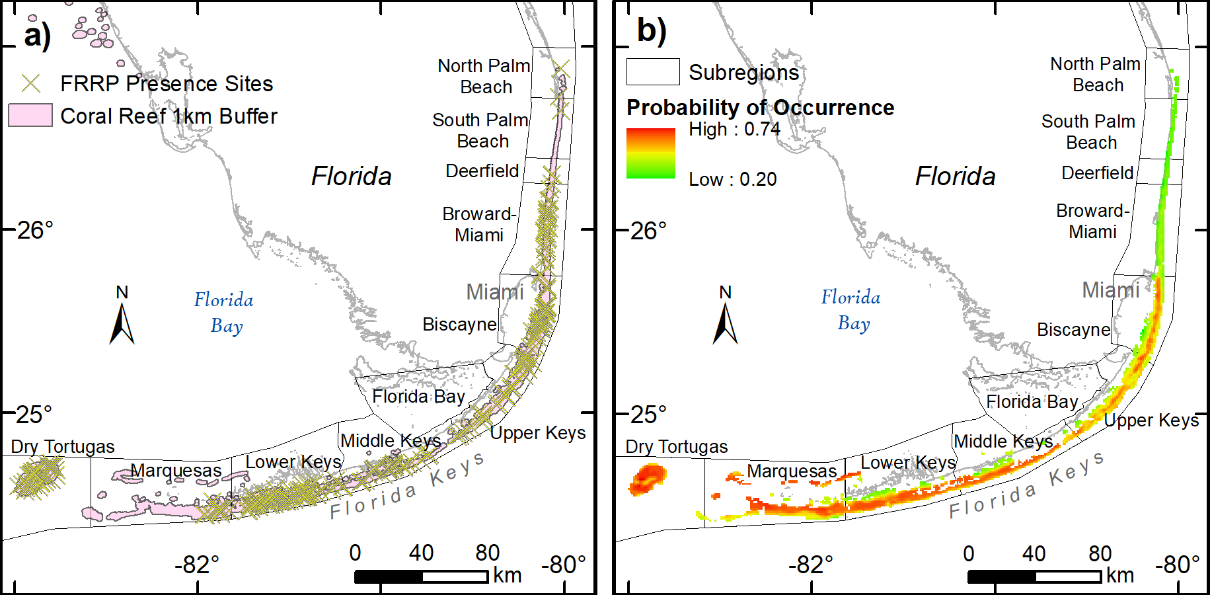
**

**
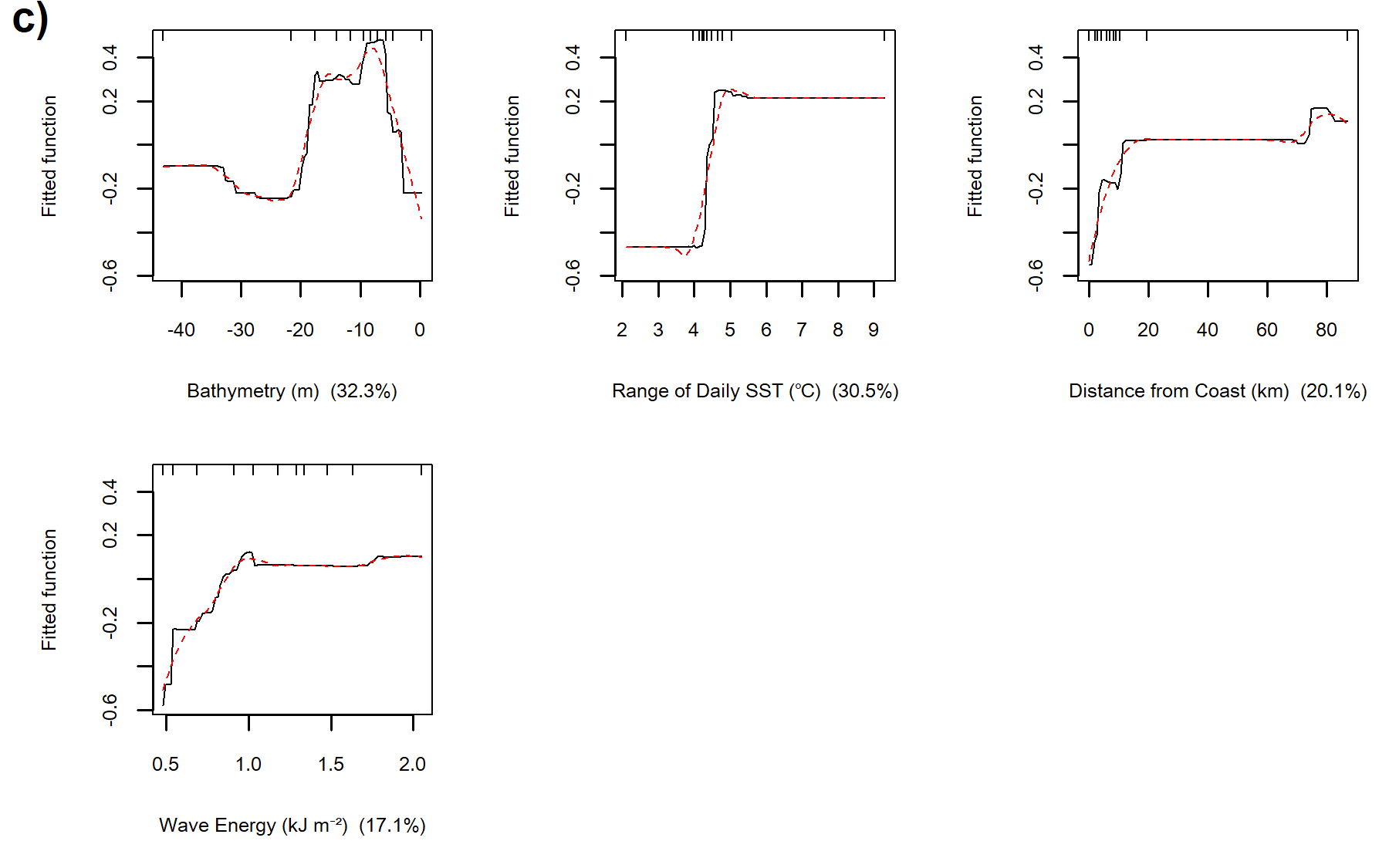
**

**Figure T.** a) Presence locations of *Pseudodiploria strigosa* used to train and test the niche model along the Florida reef tract from 2011–2015. FRRP is the Florida Reef Resiliency Project (FRRP) (yellow crosses, n = 288). Absence locations are not shown. The coral reef layer is a 1 km buffer taken from the Florida Fish and Wildlife Conservation Commission Fish and Wildlife Research Institute’s Unified Florida Reef Tract spatial layer. Basemap: Esri, DigitalGlobe, GeoEye, i-cubed, USDA FSA, USGS, AEX, Getmapping, Aerogrid, IGN, IGP, swisstopo, and the GIS User Community. b) Probability of occurrence of *Pseudodiploria strigosa*. Our niche model provides a probability map highlighting where these corals will experience ‘suitable’ environmental conditions for restoration. c) Fitted function plots of the suite of 4 predictor variables that created the most accurate model output for *Pseudodiploria strigosa.* The height of the function above or below the “0” mark shows to what degree the suitable habitat is affected, within the range of each variable. The percentage within the parentheses shows the influence of each variable on the model.
