# Supplementary material for: Niche space of corals along the Florida reef tract
Source: PLoS One. 2020 Apr 7;15(4):e0231104. doi: 10.1371/journal.pone.0231104 (PMC7138326; doi:10.1371/journal.pone.0231104)
Supplement: S4 File — (DOCX) [file pone.0231104.s004.docx]

**
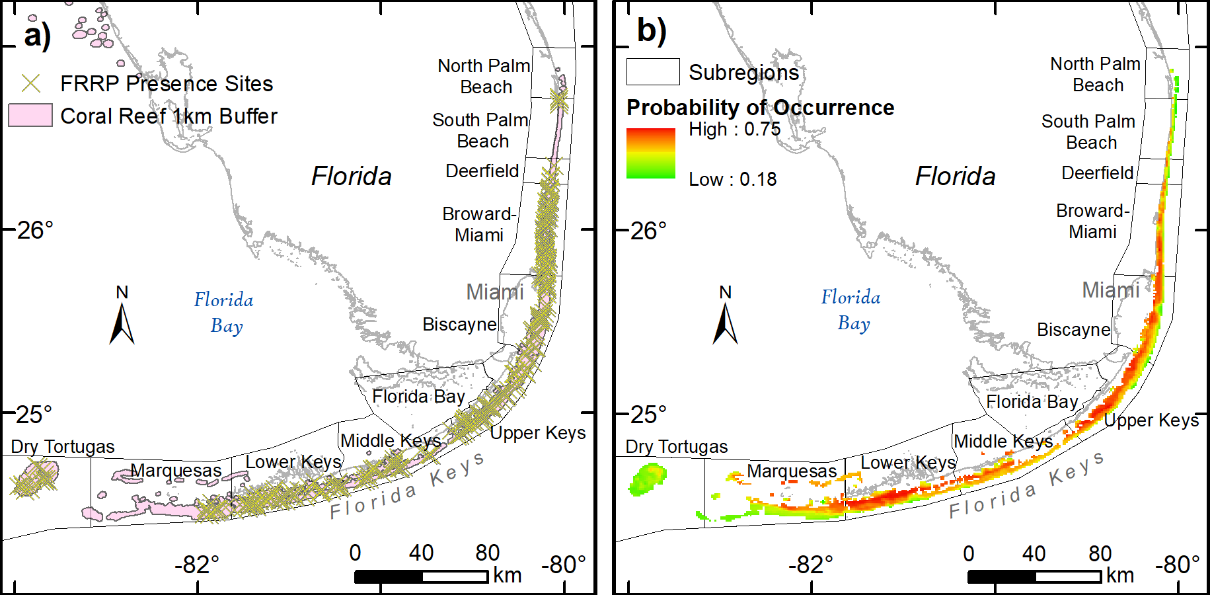
**

**
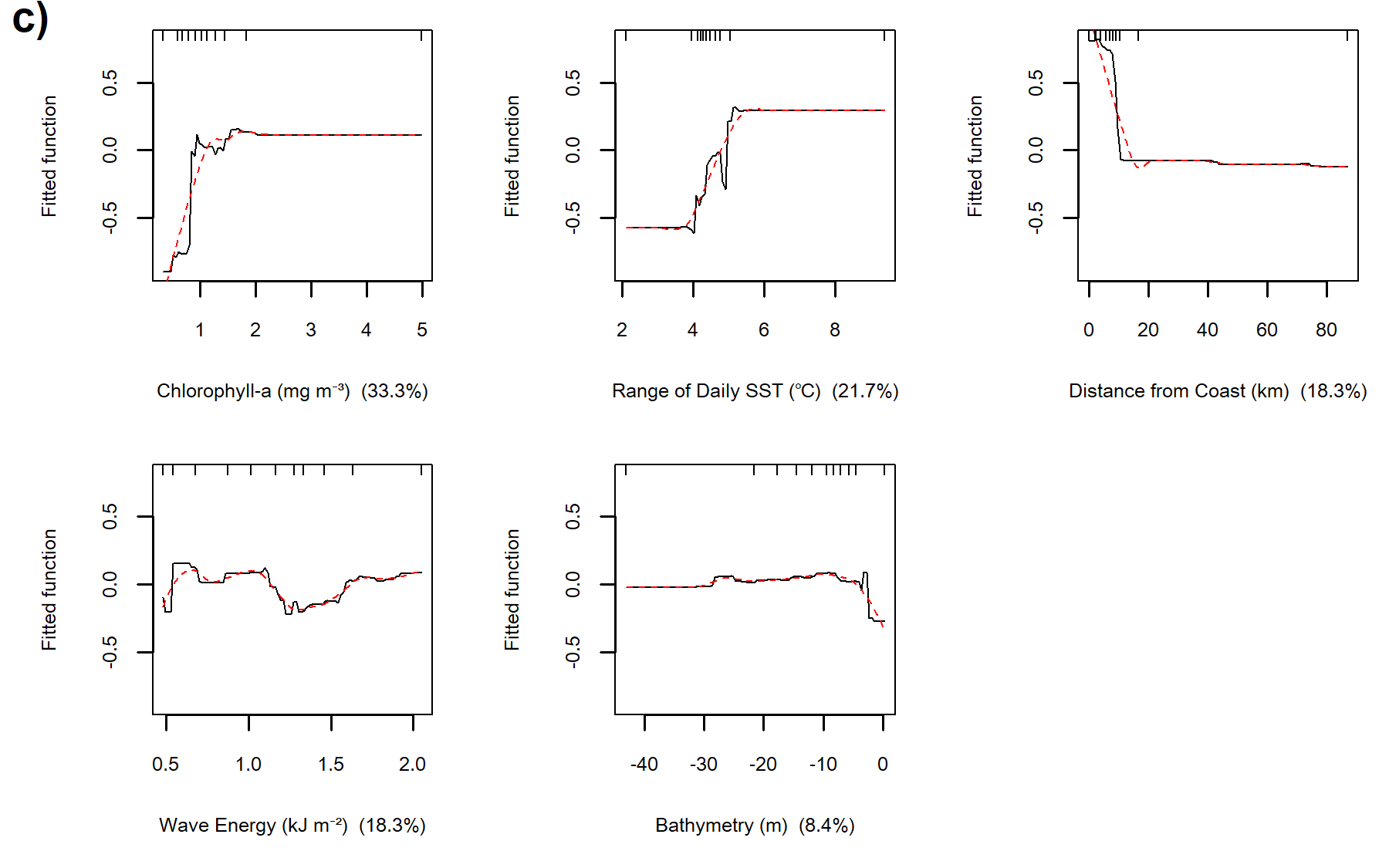
**

**Figure U.** a) Presence locations of *Solenastrea bournoni* used to train and test the niche model along the Florida reef tract from 2011–2015. FRRP is the Florida Reef Resiliency Project (FRRP) (yellow crosses, n = 304). Absence locations are not shown. The coral reef layer is a 1 km buffer taken from the Florida Fish and Wildlife Conservation Commission Fish and Wildlife Research Institute’s Unified Florida Reef Tract spatial layer. Basemap: Esri, DigitalGlobe, GeoEye, i-cubed, USDA FSA, USGS, AEX, Getmapping, Aerogrid, IGN, IGP, swisstopo, and the GIS User Community. b) Probability of occurrence of *Solenastrea bournoni*. Our niche model provides a probability map highlighting where these corals will experience ‘suitable’ environmental conditions for restoration. c) Fitted function plots of the suite of 5 predictor variables that created the most accurate model output for *Solenastrea bournoni.* The height of the function above or below the “0” mark shows to what degree the suitable habitat is affected, within the range of each variable. The percentage within the parentheses shows the influence of each variable on the model.

**
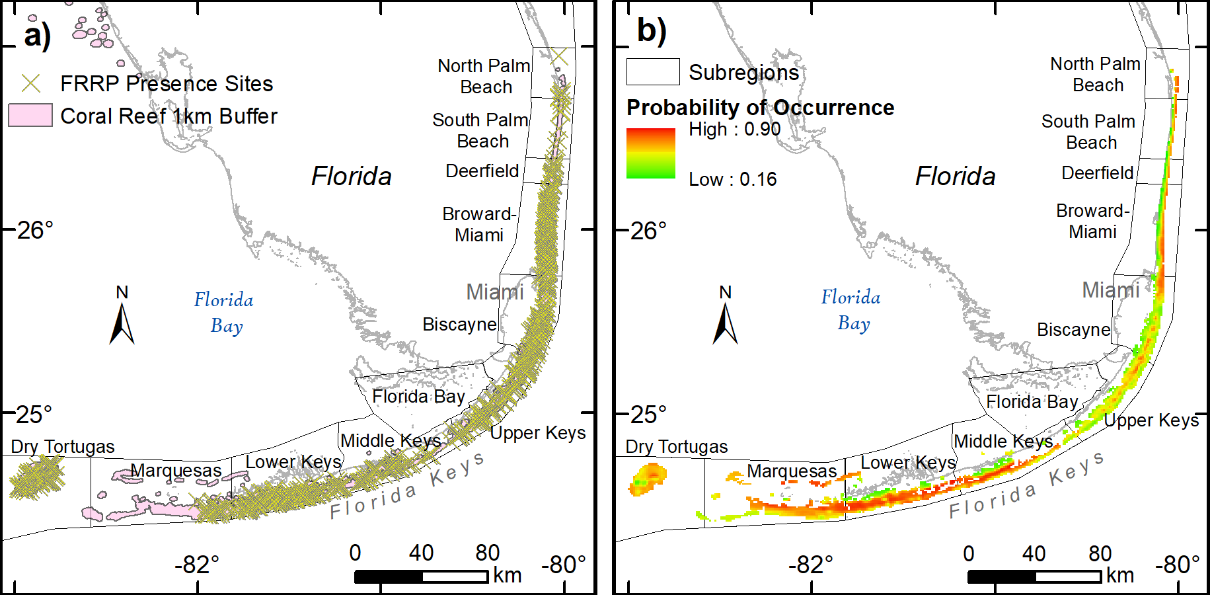
**

**
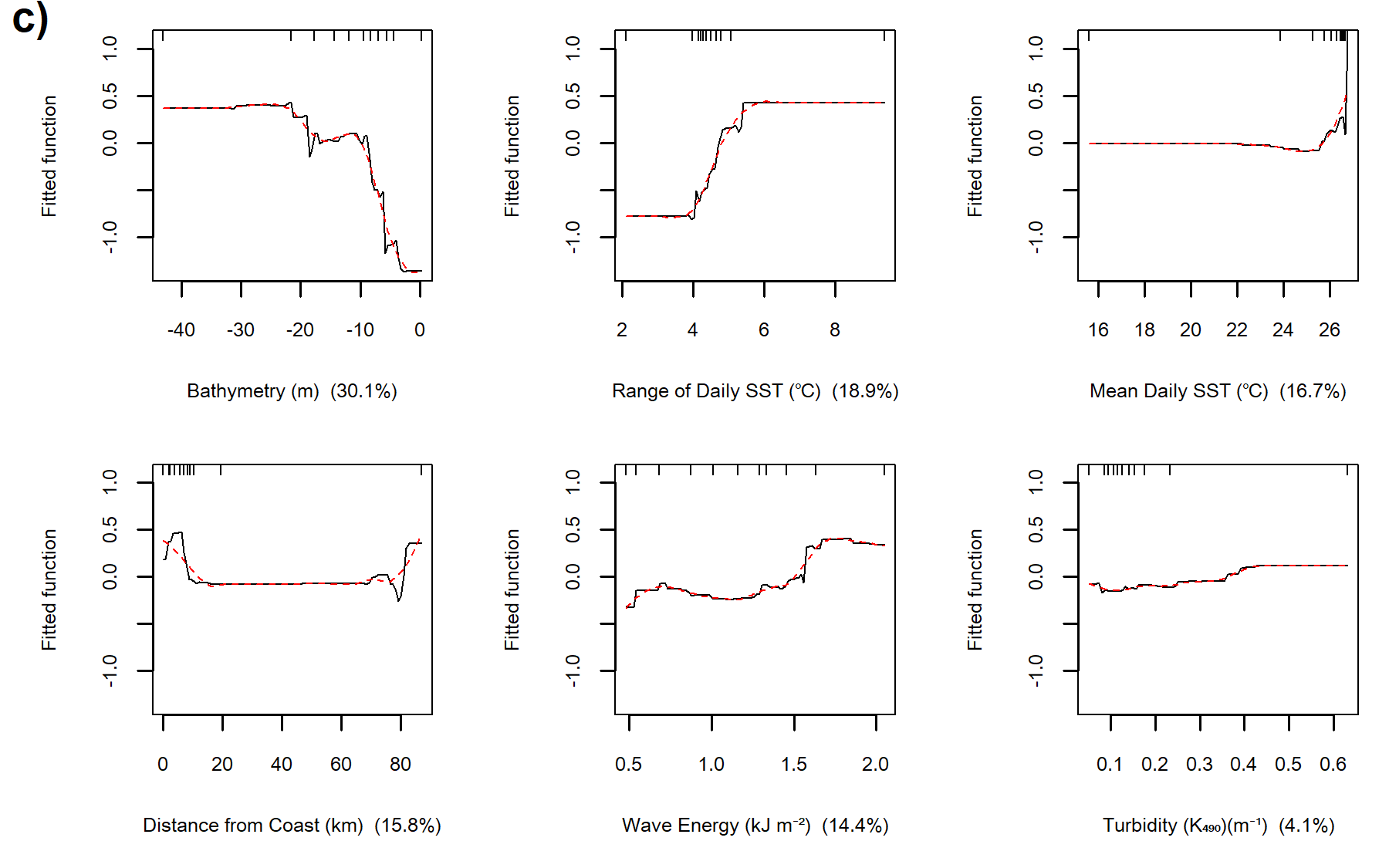
**

**Figure V.** a) Presence locations of *Stephanocoenia intersepta* used to train and test the niche model along the Florida reef tract from 2011–2015. FRRP is the Florida Reef Resiliency Project (FRRP) (yellow crosses, n = 728). Absence locations are not shown. The coral reef layer is a 1 km buffer taken from the Florida Fish and Wildlife Conservation Commission Fish and Wildlife Research Institute’s Unified Florida Reef Tract spatial layer. Basemap: Esri, DigitalGlobe, GeoEye, i-cubed, USDA FSA, USGS, AEX, Getmapping, Aerogrid, IGN, IGP, swisstopo, and the GIS User Community. b) Probability of occurrence of *Stephanocoenia intersepta*. Our niche model provides a probability map highlighting where these corals will experience ‘suitable’ environmental conditions for restoration. c) Fitted function plots of the suite of 6 predictor variables that created the most accurate model output for *Stephanocoenia intersepta.* The height of the function above or below the “0” mark shows to what degree the suitable habitat is affected, within the range of each variable. The percentage within the parentheses shows the influence of each variable on the model.


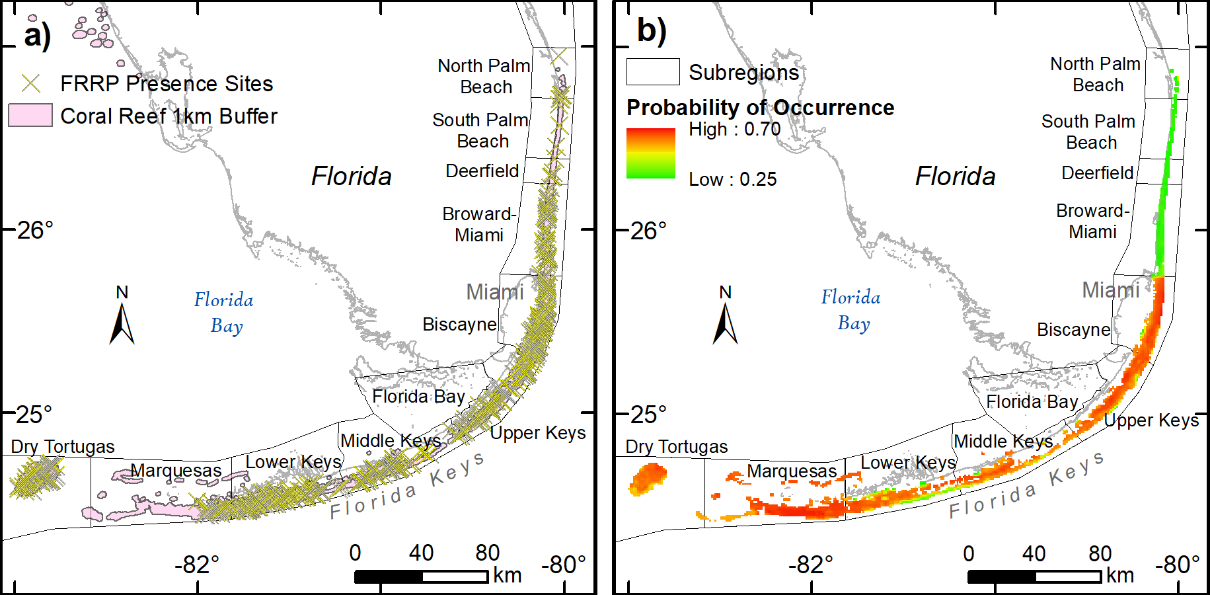


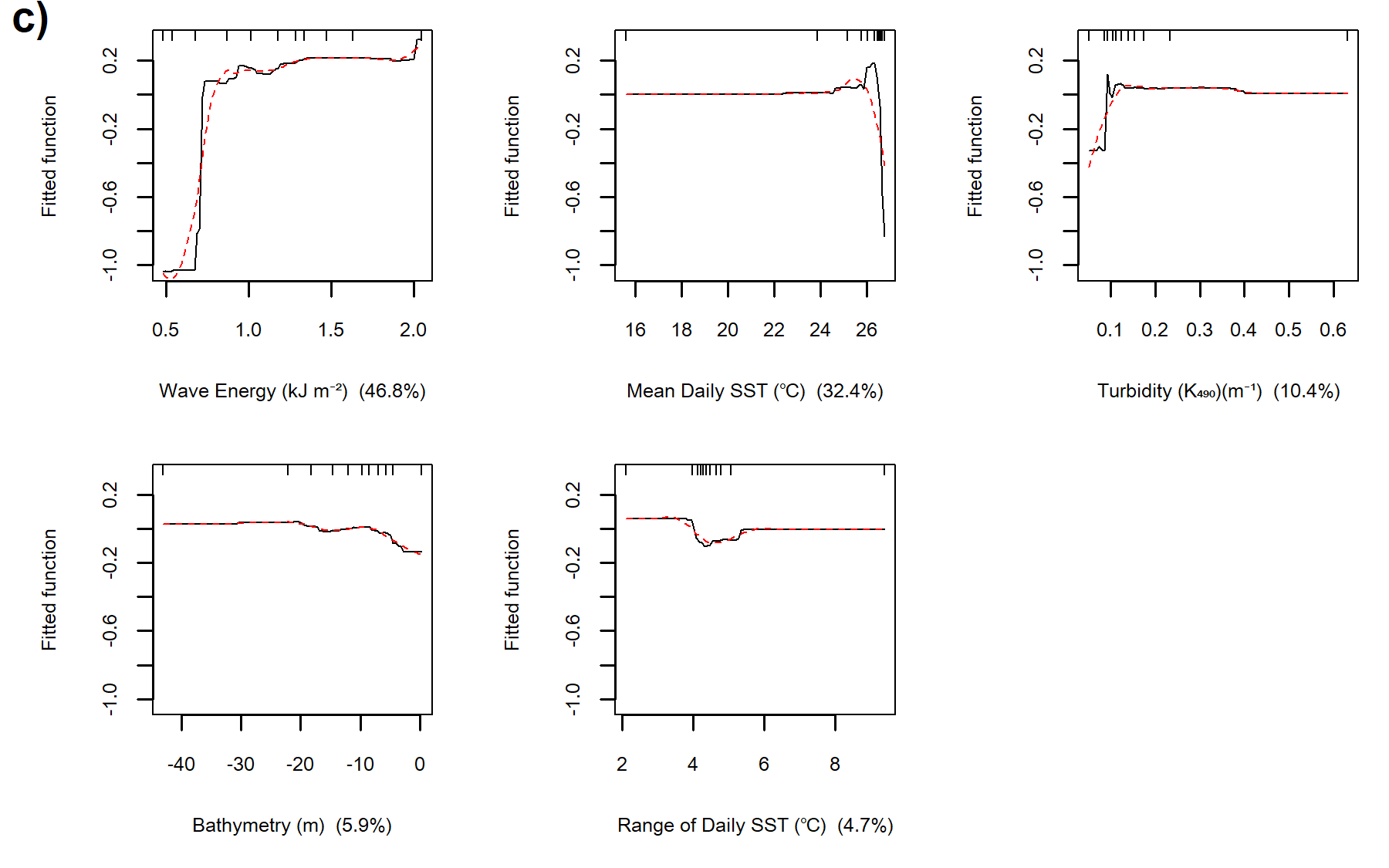


**Figure W.** a) Presence locations of *Siderastrea radians* used to train and test the niche model along the Florida reef tract from 2011–2015. FRRP is the Florida Reef Resiliency Project (FRRP) (yellow crosses, n = 465). Absence locations are not shown. The coral reef layer is a 1 km buffer taken from the Florida Fish and Wildlife Conservation Commission Fish and Wildlife Research Institute’s Unified Florida Reef Tract spatial layer. Basemap: Esri, DigitalGlobe, GeoEye, i-cubed, USDA FSA, USGS, AEX, Getmapping, Aerogrid, IGN, IGP, swisstopo, and the GIS User Community. b) Probability of occurrence of *Siderastrea radians*. Our niche model provides a probability map highlighting where these corals will experience ‘suitable’ environmental conditions for restoration. c) Fitted function plots of the suite of 5 predictor variables that created the most accurate model output for *Siderastrea radians.* The height of the function above or below the “0” mark shows to what degree the suitable habitat is affected, within the range of each variable. The percentage within the parentheses shows the influence of each variable on the model.

**
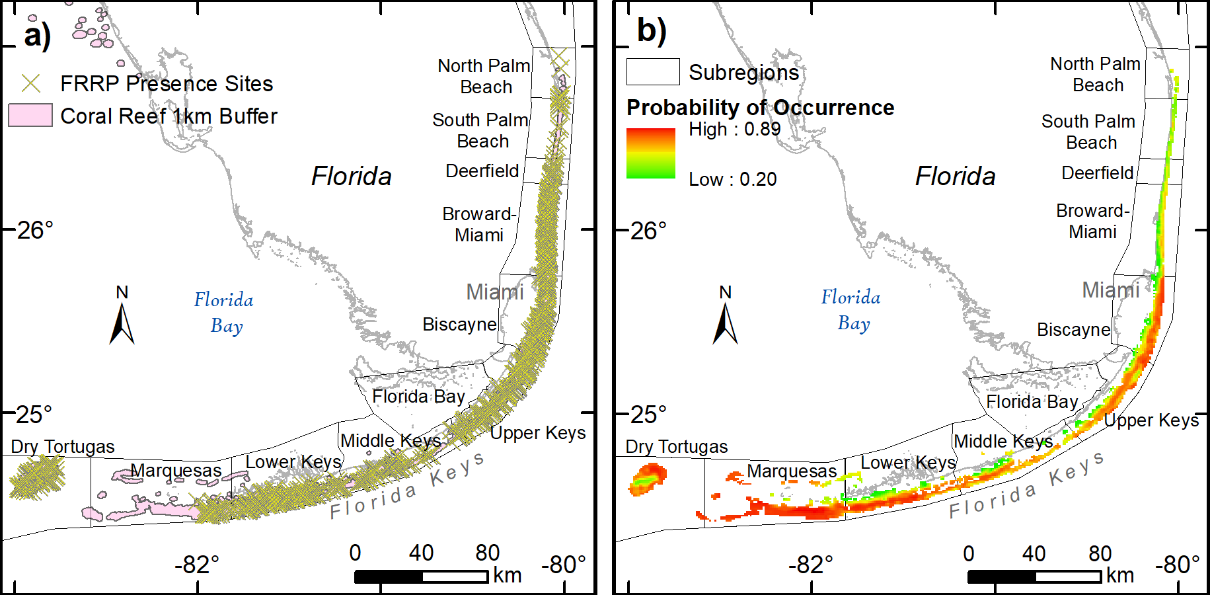
**

**
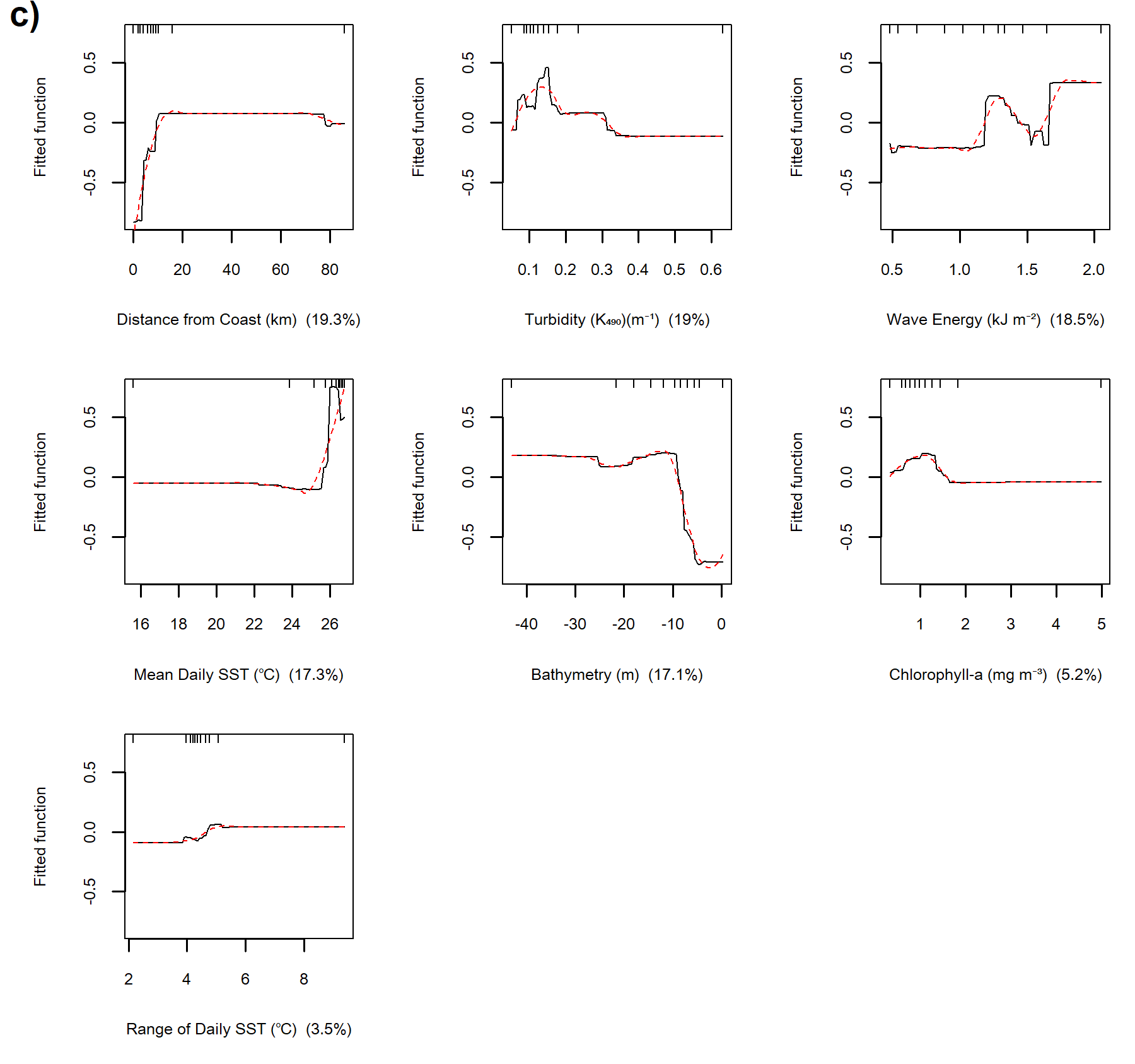
**

**Figure X**. a) Presence locations of *Siderastrea siderea* used to train and test the niche model along the Florida reef tract from 2011–2015. FRRP is the Florida Reef Resiliency Project (FRRP) (yellow crosses, n = 888). Absence locations are not shown. The coral reef layer is a 1 km buffer taken from the Florida Fish and Wildlife Conservation Commission Fish and Wildlife Research Institute’s Unified Florida Reef Tract spatial layer. Basemap: Esri, DigitalGlobe, GeoEye, i-cubed, USDA FSA, USGS, AEX, Getmapping, Aerogrid, IGN, IGP, swisstopo, and the GIS User Community. b) Probability of occurrence of *Siderastrea siderea*. Our niche model provides a probability map highlighting where these corals will experience ‘suitable’ environmental conditions for restoration. c) Fitted function plots of the suite of 7 predictor variables that created the most accurate model output for *Siderastrea siderea.* The height of the function above or below the “0” mark shows to what degree the suitable habitat is affected, within the range of each variable. The percentage within the parentheses shows the influence of each variable on the model.

**Model testing**

For each confusion matrix, the columns represent observed presence and absence points, and the rows represent predicted presence and absence points of each coral species. Accuracy is defined as the proportion of total predictions that were correct (True positives + True negatives/All Positives + All Negatives). Sensitivity is the true positive rate (True positive/ True positive + False negative). Specificity is the true negative rate (True negative/ True negative + False positive) (Table S2). Three of the most useful indicators of model correctness are sensitivity, specificity, and accuracy. Sensitivity is an indicator of how good the model is at detecting true positives. Specificity is an indicator of how good the model is at detecting true negatives (Table S2). Accuracy is a combination of the efficiency of predicting true positives and true negatives.

**Table B.** Calculations for precision, negative precision, specificity, sensitivity, and accuracy based on model predictions and observed data. Top left is the number of true negatives (TN), bottom left is the number of false negatives (FN), top right is the number of false positives (FP), and bottom right is the number of true positives (TP).

|  | | **Observed** | |  |
| --- | --- | --- | --- | --- |
|  |  | Absence | Presence |  |
| **Predicted** | Absence | **TN** | **FN** | **Neg. Precision**  = TN/(TN+FN) |
|  | Presence | **FP** | **TP** | **Precision**  = TP/(FP+TP) |
|  | | **Specificity**  = TN/(TN+FP) | **Sensitivity**  = TP/(FN+TP) | **Accuracy**  = (TN+TP)/ (TN+FN+FP+TP) |

**Model accuracy**

The *Acropora cervicornis* model had high specificity but low sensitivity. Overall the model was 87% accurate (Table S3). The accuracy of the models for non-*Acropora* species was variable, ranging from low accuracy for *Stephanocoenia intersepta* (57%) and a high accuracy for *Orbicella franksi* (88%) (Table S3). Most of the models for the other coral species also had high specificity and low sensitivity.

**Table C.** Confusion matrices and statistics, testing the niche-model predictions of each of 23 coral species’ niche space along the Florida reef tract at 985 sites from 2011–2015 against the set aside 20% testing dataset. Where TN is true negative, FN is false negative, FP is false positive, and TP is true positive.

| **Species** | **Raw Data** | | | | **Statistics** | | | | |
| --- | --- | --- | --- | --- | --- | --- | --- | --- | --- |
|  | **TN** | **FN** | **FP** | **TP** | **Neg. Precision** | **Precision** | **Specificity** | **Sensitivity** | **Accuracy** |
| ***Acropora cervicornis*** | 163 | 10 | 15 | 9 | 94.2% | 37.5% | 91.6% | 47.4% | **87.3%** |
| ***Undaria (Agaricia) agaricites*** | 83 | 36 | 21 | 57 | 69.7% | 73.1% | 79.8% | 61.3% | **71.1%** |
| ***Colpophyllia natans*** | 134 | 31 | 7 | 25 | 81.2% | 78.1% | 95.0% | 44.6% | **80.7%** |
| ***Dichocoenia stokesi*** | 56 | 68 | 7 | 66 | 45.2% | 90.4% | 88.9% | 49.3% | **61.9%** |
| ***Pseudodiploria clivosa*** | 145 | 20 | 25 | 7 | 87.9% | 21.9% | 85.3% | 25.9% | **77.2%** |
| ***Diploria labyrinthiformis*** | 126 | 26 | 28 | 17 | 82.9% | 37.8% | 81.8% | 39.5% | **72.6%** |
| ***Pseudodiploria strigosa*** | 124 | 35 | 16 | 22 | 78.0% | 57.9% | 88.6% | 38.6% | **74.1%** |
| ***Eusmilia fastigiata*** | 153 | 28 | 8 | 8 | 84.5% | 50.0% | 95.0% | 22.2% | **81.7%** |
| ***Madracis decactis*** | 160 | 12 | 17 | 8 | 93.0% | 32.0% | 90.4% | 40.0% | **85.3%** |
| ***Meandrina meandrites*** | 126 | 52 | 7 | 12 | 70.8% | 63.2% | 94.7% | 18.8% | **70.1%** |
| ***Millepora alcicornis*** | 20 | 65 | 3 | 109 | 23.5% | 97.3% | 87.0% | 62.6% | **65.5%** |
| ***Montastraea cavernosa*** | 75 | 65 | 5 | 52 | 53.6% | 91.2% | 93.8% | 44.4% | **64.5%** |
| ***Orbicella faveolata*** | 117 | 37 | 20 | 23 | 76.0% | 53.5% | 85.4% | 38.3% | **71.1%** |
| ***Orbicella franksi*** | 159 | 13 | 10 | 15 | 92.4% | 60.0% | 94.1% | 53.6% | **88.3%** |
| ***Mycetophyllia sp.*** | 156 | 6 | 16 | 19 | 96.3% | 54.3% | 90.7% | 76.0% | **88.8%** |
| ***Porites astreoides*** | 24 | 79 | 5 | 89 | 23.3% | 94.7% | 82.8% | 53.0% | **57.4%** |
| ***Porites divaricata*** | 126 | 22 | 36 | 13 | 85.1% | 26.5% | 77.8% | 37.1% | **70.6%** |
| ***Porites furcata*** | 127 | 11 | 45 | 14 | 92.0% | 23.7% | 73.8% | 56.0% | **71.6%** |
| ***Porites porites*** | 61 | 42 | 20 | 74 | 59.2% | 78.7% | 75.3% | 63.8% | **68.5%** |
| ***Siderastrea radians*** | 81 | 52 | 16 | 48 | 60.9% | 75.0% | 83.5% | 48.0% | **65.5%** |
| ***Siderastrea siderea*** | 19 | 45 | 6 | 127 | 29.7% | 95.5% | 76.0% | 73.8% | **74.1%** |
| ***Solenastrea bournoni*** | 114 | 33 | 24 | 26 | 77.6% | 52.0% | 82.6% | 44.1% | **71.1%** |
| ***Stephanocoenia intersepta*** | 34 | 71 | 14 | 78 | 32.4% | 84.8% | 70.8% | 52.3% | **56.9%** |

**Table D.** Suitable habitat area (%) for each of 23 coral species, at a 50% probability-of-occurrence threshold predicted by the niche model, at each of 10 geographical subregions along the Florida reef tract at 985 sites from 2011–2015. The buffered extent of the model did not extend into the Florida Bay subregion. The average and rank of suitable habitat area (%) for all 23 coral species was calculated for each of the 10 subregions.

| Species | Marquesas | N Palm Beach | S Palm Beach | Deerfield | Broward-Miami | Biscayne | Middle Keys | Dry Tortugas | Lower Keys | Upper Keys | Total Area |
| --- | --- | --- | --- | --- | --- | --- | --- | --- | --- | --- | --- |
| *Acropora cervicornis* | 10.03 | 0 | 10.71 | 18.60 | 44.83 | 14.04 | 5.91 | 57.78 | 37.36 | 25.87 | 24.20 |
| *Undaria (Agaricia) agaricites* | 77.21 | 0 | 0 | 0 | 0 | 45.27 | 57.26 | 82.22 | 45.02 | 47.58 | 51.36 |
| *Colpophyllia natans* | 72.28 | 0 | 0 | 0 | 0 | 13.87 | 50.40 | 83.70 | 65.82 | 25.06 | 46.33 |
| *Dichocoenia stokesi* | 57.65 | 0 | 0 | 0 | 0.50 | 58.96 | 76.28 | 0 | 79.10 | 83.99 | 55.48 |
| *Pseudodiploria clivosa* | 41.84 | 0 | 4.76 | 9.30 | 8.37 | 56.73 | 51.88 | 53.33 | 54.60 | 60.97 | 46.77 |
| *Diploria labyrinthiformis* | 62.76 | 0 | 0 | 0 | 0 | 33.81 | 53.76 | 89.26 | 56.90 | 50.81 | 49.88 |
| *Pseudodiploria strigosa* | 73.81 | 0 | 0 | 0 | 0 | 41.83 | 69.09 | 94.07 | 73.18 | 47.81 | 57.97 |
| *Eusmilia fastigiata* | 62.41 | 0 | 0 | 0 | 0 | 31.79 | 60.65 | 74.81 | 63.28 | 39.91 | 48.73 |
| *Madracis decactis* | 48.30 | 87.88 | 55.95 | 43.24 | 21.57 | 0.58 | 0.54 | 67.14 | 6.45 | 1.85 | 22.87 |
| *Meandrina meandrites* | 20.75 | 90.91 | 61.90 | 51.35 | 37.81 | 7.80 | 16.98 | 60.37 | 24.02 | 4.18 | 24.12 |
| *Millepora alcicornis* | 34.18 | 51.52 | 52.38 | 40.54 | 33.33 | 63.58 | 59.84 | 100 | 67.19 | 64.04 | 58.34 |
| *Montastraea cavernosa* | 72.62 | 93.94 | 58.33 | 45.95 | 47.76 | 30.35 | 47.98 | 93.70 | 69.53 | 18.79 | 55.45 |
| *Orbicella faveolata* | 79.25 | 0 | 0 | 0 | 0 | 40.11 | 52.69 | 76.67 | 70.69 | 59.58 | 56.45 |
| *Orbicella franksi* | 77.55 | 0 | 0 | 0 | 0 | 30.09 | 9.41 | 94.81 | 10.34 | 33.95 | 36.34 |
| *Mycetophyllia sp.* | 59.48 | 0 | 5.95 | 13.51 | 1.96 | 0.58 | 26.95 | 84.64 | 44.14 | 1.39 | 32.65 |
| *Porites astreoides* | 88.44 | 6.06 | 0 | 0 | 11.33 | 47.56 | 59.14 | 96.67 | 62.84 | 37.64 | 58.09 |
| *Porites divaricata* | 18.71 | 0 | 0 | 0 | 0 | 53.87 | 82.26 | 29.63 | 66.28 | 61.20 | 44.70 |
| *Porites furcata* | 53.06 | 0 | 0 | 0 | 0 | 60.17 | 65.86 | 75.19 | 30.65 | 48.04 | 46.17 |
| *Porites porites* | 71.60 | 0 | 0 | 0 | 0 | 33.53 | 58.49 | 100 | 55.47 | 62.18 | 54.86 |
| *Siderastrea radians* | 100.00 | 0 | 0 | 0 | 0 | 93.98 | 90.05 | 100 | 77.39 | 94.00 | 80.50 |
| *Siderastrea siderea* | 91.16 | 39.39 | 11.90 | 21.62 | 48.26 | 73.99 | 77.09 | 86.30 | 75.20 | 80.51 | 75.57 |
| *Solenastrea bournoni* | 47.11 | 3.03 | 21.43 | 29.73 | 68.66 | 69.36 | 81.13 | 0.74 | 81.84 | 83.76 | 61.54 |
| *Stephanocoenia intersepta* | 84.01 | 75.76 | 54.76 | 39.53 | 54.19 | 37.25 | 68.55 | 84.81 | 80.27 | 41.80 | 65.79 |
| Average | 61.05 | 19.50 | 14.70 | 13.63 | 16.46 | 40.83 | 53.14 | 73.30 | 56.42 | 46.73 | - |
| Rank | 2 | 7 | 9 | 10 | 8 | 6 | 4 | 1 | 3 | 5 | - |


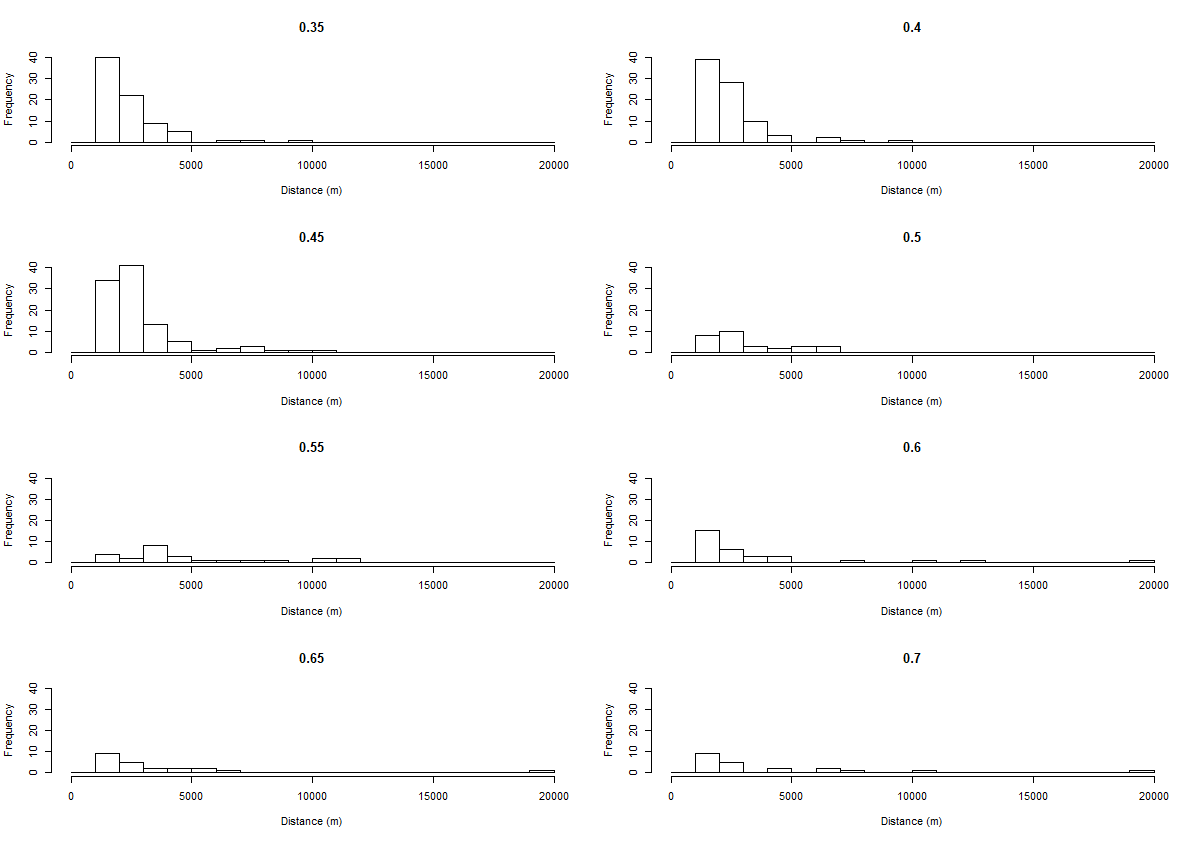


**Figure Y**. Frequency of distances between predicted viable patches *Acropora cervicornis* colonies along the Florida reef tract. We computed the Euclidean nearest-neighbor distance of predicted niche patches to examine the average distances between the patches using a series of probability-of-occurrence thresholds. We used the predicted probability of occurrence from the niche model as a continuous probability raster file, which was converted to a binary raster map using a series of ‘cutoff’ thresholds to understand the spatial attributes of the species niche. These thresholds simulate different intensities of disturbance, with increasing values simulating increasing intensities of disturbance. We found that on modern reefs along the Florida reef the distances between the patches of *Acropora cervicornis* are likely to increase with an increase in disturbance, which is troublesome considering the fragile nature of the modern metapopulation.

**R code for disturbance simulations**

library(rstudioapi)

# Set Working Directory to the directory of this file

setwd(dirname(rstudioapi::getActiveDocumentContext()$path))

library(raster)

library(rgdal)

library(stringr)

library(rworldmap)

library(gbm)

library(SDMTools)

library(stats)

library(spatialEco)

library(corrplot)

library(stats)

library(spatialEco)

source("brt.functions.R")

utm <- "+proj=utm +zone=17 +units=km +datum=WGS84"

utmMeters <- "+proj=utm +zone=17 +units=m +datum=WGS84"

acerRaster <- raster("./results/ACERpredictions.asc")

proj4string(acerRaster) <- utm

acerRasterMeters <- projectRaster(acerRaster, crs=utmMeters)

library(landscapemetrics)

par(mfrow=c(4, 2))

lim <- 20000

for (cutoff in seq(0.35, 0.7, 0.05)) {

acerRasterMetersInt <- acerRasterMeters > cutoff

enn <- lsm_p_enn(acerRasterMetersInt)

enn <- subset(enn, class == 1)

vals <- enn$value

vals <- vals[vals<=lim]

hist(vals, main=toString(cutoff), breaks=seq(0, lim, 1000))

}

**References**

1. Ekebom J, Laihonen P, Suominen T. A GIS-based step-wise procedure for assessing physical exposure in fragmented archipelagos. Estuarine, Coastal and Shelf Science. 2003; 57: 887-898. doi: 10.1016/S0272-7714(02)00419-5.
2. Chollett I, Mumby PJ. Predicting the distribution of Montastraea reefs using wave exposure. Coral Reefs. 2012; 31: 493-503.
3. R Core Team. R: A language and environment for statistical computing. Vienna, Australia: R Foundation for Statistical Computing. 2017. <http://www.R-project.org/>.
4. Hijmans RJ. raster: Geographic data analysis and modeling. R package version 2.5-8. 2016. Available from: <https://CRAN.R-project.org/package=raster>.
